# The Risk of Deterioration in GCS13–15 Patients with Traumatic Brain Injury Identified by Computed Tomography Imaging: A Systematic Review and Meta-Analysis

**DOI:** 10.1089/neu.2017.5259

**Published:** 2018-03-01

**Authors:** Carl Marincowitz, Fiona E. Lecky, William Townend, Aditya Borakati, Andrea Fabbri, Trevor A. Sheldon

**Affiliations:** ^1^ Hull York Medical School, University of Hull, Hull, United Kingdom.; ^2^ School of Health and Related Research, University of Sheffield, Sheffield, United Kingdom.; ^3^ Emergency Department, Hull and East Yorkshire NHS Trust, Hull, United Kingdom.; ^4^ Hull York Medical School, University of Hull, Hull, United Kingdom.; ^5^ Emergency Unit, Presidio Ospedaliero Morgagni-Pierantoni, AUSL della Romagna, Forlì, Italy.; ^6^ Department of Health Sciences, University of York, Alcuin Research Resource Center, Heslington, York, United Kingdom.

**Keywords:** intra-cranial hemorrhage, mild traumatic brain injury, minor head injury, prognostic modeling

## Abstract

The optimal management of mild traumatic brain injury (TBI) patients with injuries identified by computed tomography (CT) brain scan is unclear. Some guidelines recommend hospital admission for an observation period of at least 24 h. Others argue that selected lower-risk patients can be discharged from the Emergency Department (ED). The objective of our review and meta-analysis was to estimate the risk of death, neurosurgical intervention, and clinical deterioration in mild TBI patients with injuries identified by CT brain scan, and assess which patient factors affect the risk of these outcomes. A systematic review and meta-analysis adhering to PRISMA standards of protocol and reporting were conducted. Study selection was performed by two independent reviewers. Meta-analysis using a random effects model was undertaken to estimate pooled risks for: clinical deterioration, neurosurgical intervention, and death. Meta-regression was used to explore between-study variation in outcome estimates using study population characteristics. Forty-nine primary studies and five reviews were identified that met the inclusion criteria. The estimated pooled risk for the outcomes of interest were: clinical deterioration 11.7% (95% confidence interval [CI]: 11.7%–15.8%), neurosurgical intervention 3.5% (95% CI: 2.2%–4.9%), and death 1.4% (95% CI: 0.8%–2.2%). Twenty-one studies presented within-study estimates of the effect of patient factors. Meta-regression of study characteristics and pooling of within-study estimates of risk factor effect found the following factors significantly affected the risk for adverse outcomes: age, initial Glasgow Coma Scale (GCS), type of injury, and anti-coagulation. The generalizability of many studies was limited due to population selection. Mild TBI patients with injuries identified by CT brain scan have a small but clinically important risk for serious adverse outcomes. This review has identified several prognostic factors; research is needed to derive and validate a usable clinical decision rule so that low-risk patients can be safely discharged from the ED.

## Introduction

There are 1.4 million annual attendances in England and Wales to Emergency Departments (EDs) following a head injury (any trauma to the head), and in 2010, 2.5 million people were treated for traumatic brain injury (TBI; injury to the brain or alteration of brain function due to an external force) in the United States.^1^ Approximately 95% of patients have an initial Glasgow Coma Scale (GCS) of 13–15, out of a possible 15, indicating normal or mildly impaired responsiveness and orientation.^1,2^ In this large group with head injury and a high conscious level at presentation, research has focused on developing decision rules to identify patients who require computed tomography (CT) imaging due to their risk for life-threatening TBI.

In the United Kingdom (UK), National Institute for Health and Care Excellence (NICE) and Scottish Intercollegiate Guidelines Network (SIGN) guidelines are used for this risk assessment, based on the Canadian CT head Rule (CCHR).^1,3,4^ Only 1% of head-injured patients have life-threatening TBI.^1,4^ However, 7% have TBI identified by CT imaging.^5^

Most TBI patients who require neurosurgical intervention are identified soon after presentation. The optimal management of the remaining patients in this group remains controversial. A proportion will deteriorate due to the progression of their injuries, and so some studies advocate admission to higher dependency levels of care and repeat CT imaging.^6,7^

Other studies report that some low-risk patients may be safely discharged after a short period of observation in the ED.^8,9^ Perel and colleagues have previously outlined how prognostic models can aid clinical decision-making in TBI.^10^ Subsequent prognostic models, including the IMPACT, TARN, and CRASH models, have been useful in predicting adverse outcomes in patients with more severe TBI, but they are not applicable to this patient group.^11–13^ Equivalent prognostic models for GCS13–15 patients with CT-identified TBI may help safely reduce hospital admissions.

This review is the first to give an overview of the risk for adverse outcomes and prognostic factors in patients with mild TBI (a high or normal conscious level with traumatically induced brain dysfunction) and injuries identified by CT brain scan. The review specifically:

(i) Estimates the overall risk for adverse outcomes in patients who are initially GCS13–15 in the ED when TBI is identified by CT imaging.
(ii) Assesses which prognostic factors affect the risk for deterioration and other clinically important outcomes in this population.

## Methods

A systematic review was conducted using the PRISMA P protocol and is reported in accordance with PRISMA guidelines.^14^ The review is registered with the PROSPERO prospective register of systematic reviews and (protocol is available at http://www.crd.york.ac.uk/PROSPERO/display_record.asp?ID=CRD42016051585).

### Inclusion criteria

#### Participants

Criteria were patients aged ≥12 years with an initial GCS of 13–15 with TBI identified by CT imaging. TBI included any traumatic extra-dural hemorrhage, subdural hemorrhage, intra-cerebral hemorrhage, subarachnoid hemorrhage, cerebral contusion, or skull fracture. Studies had to be conducted in the context of an emergency hospital attendance including a presentation to the ED or during admission to an inpatient ward.

#### Prognostic factors

Factors potentially affecting the risk for adverse outcomes were included in the analysis if they were patient factors present at admission including: demographic characteristics, co-morbidities, medication use, symptoms, other clinical features, or factors available from initial investigations.

#### Outcome measures

Primary outcomes were: death, neurosurgical intervention, or any other measure of clinical deterioration such that admission to a hospital was warranted. Secondary outcome was: progression of TBI on repeat CT imaging.

#### Types of study design

All studies, other than case studies, were included.

### Search methods for study identification

Studies published before 1996 were excluded due to more liberal use of CT imaging to diagnose TBI after this date.^5^

The following electronic databases were searched with results restricted to English language studies:

• EMBASE (via OVID) searched 11/24/2016: 1996 to 2016, week 47;
• MEDLINE (R) (via OVID) searched 11/24/2016: 1996 to November, week 3, 2016;
• CINHAL plus (via EBSCO) searched 11/24/2016: 1983 to 2016;
• Cochrane Central Register of Controlled Trials (CENTRAL); The Cochrane Library,2016, all available dates. Accessed 11/24/2016.

The full search strategy is reported in Supplementary Table 1 (see online supplementary material at http://www.liebertpub.com).

The reference and citation searches of several national guidelines, reports, and reviews included: NICE, SIGN, and Australian New South Wales (NSW) guidelines; National Institute for Health Research (NIHR) Health Technology Assessment of management strategies for minor head injury; the results of the World Health Organization (WHO) collaboration on prognosis in mild TBI; systematic reviews assessing prognostic factors in TBI; and systematic reviews assessing the utility of repeat CT imaging in minor head injury.^1,3,10,15–20^ All included studies' references and citations were searched.

The Trauma Audit and Research Network (TARN)-listed publications were searched via the TARN website (https://www.tarn.ac.uk/Content.aspx?ca=9&c=70; accessed 3/10/2017).

### Data management and extraction

Identified studies were stored in EndNote X8 and duplicates removed.

#### Study selection

Two reviewers (CM and AB) independently completed title and abstract screening. Full reports of any studies that potentially met the inclusion were selected and assessed. These were screened, and studies that did not meet the inclusion criteria were discarded with documented reasons. Disagreements were resolved through discussion or arbitration by a third reviewer (TS).

#### Data extraction

The following data were extracted using a pre-piloted data extraction tool: study population and demographics, sample size, outcomes assessed, prognostic factors assessed, whether uni-variable or multi-variable modeling had been undertaken, and the overall results of the study. The selection criteria of studies were recorded to assess whether sub-populations with different risk profiles had been studied. The data extracted are presented in Supplementary Table 2 (see online supplementary material at http://www.liebertpub.com).

#### Assessment of the risk of bias

The Quality in Prognostic Studies (QUIPS) tool was used to assess the quality of included studies, particularly for the risk of bias.^21^ Six domains were assessed: study participation, study attrition, prognostic factor measurement, outcome measurement, study confounding, and statistical analysis and reporting.

### Statistical analysis

Three forms of analysis were undertaken: pooling of adverse outcomes reported in studies, identification of risk factors by exploration of between-study variation in outcomes by study characteristics, and a synthesis of common risk factors assessed within studies.

A pooled prevalence of the adverse outcomes of interest and confidence intervals for individual studies were estimated using the Metaprop function (STATA-SE 14).^22^ The Freeman-Tukey double arscine transformation was used to include studies with no adverse outcomes, and a random effects model was used due to study heterogeneity.^23^

Between-study heterogeneity estimates of outcomes were explored using subgroup analysis. Meta-regression of study characteristics was used to identify factors that affected the risk for the outcomes of interest. Meta-regression of multiple study characteristics' effect on the prevalence of adverse outcomes was assessed using the Metareg function (STATA-SE 14) with weighting incorporating a measure of between-study variation (tau2).^24,25^ The log odds of clinical deterioration, neurosurgical intervention, and death were assessed as dependent variables and the standard error of the log odds was used to approximate the within-study standard error. To account for studies with no outcomes, 0.5 was added to both the outcome estimates and the sample size (consequently, in graphic representations of the meta-regression the estimated risk can only tend toward zero).

Where studies had assessed the effect of risk factors on the outcomes of interest using individual data, analysis was categorized as uni-variable or multi-variable. Uni-variable meta-analysis of prognostic factor effect estimates reported in primary studies was completed using Review Manager 5.3 where possible.^26^ A random effects model was used due to the heterogeneity of study populations, prognostic factor, and outcome measures.^23^ Meta-analysis of multi-variable models was not possible due to limited numbers and variation in outcome and prognostic factor measurement.

## Results

### Search result

**FIG. 1.**
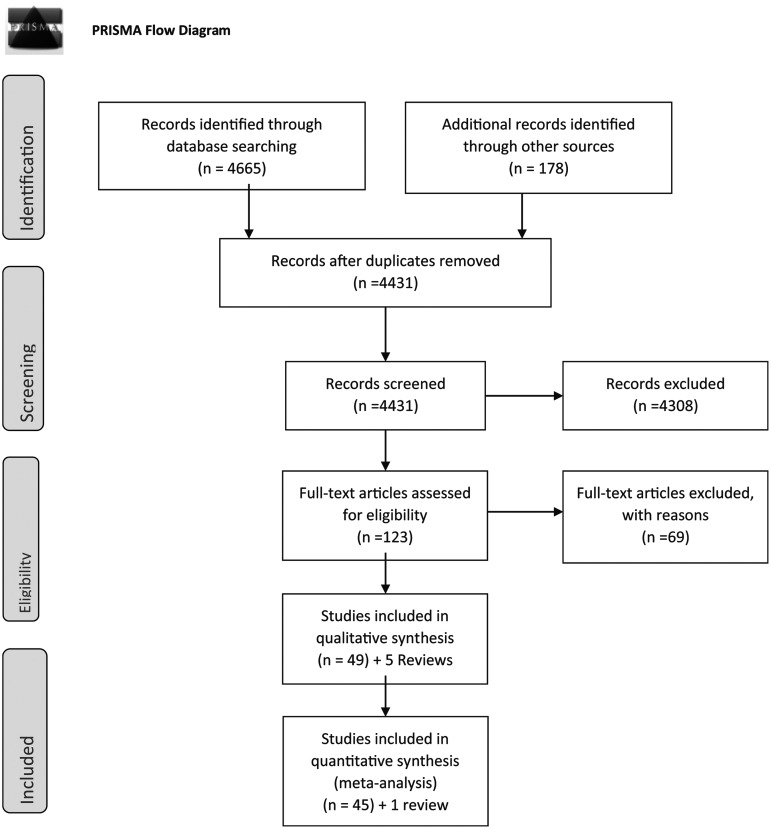
PRISMA flow-diagram showing selection of studies for inclusion in the systematic review.

The electronic search strategy was completed on November 24,2016, and identified 4665 studies. Of these, 412 were duplicates, leaving 4253 studies for title and abstract screening (Fig. 1). Following title and abstract screening, 69 studies^6,9,27–93^ and two reviews^19,20^ were retrieved. A “gray” literature search identified a further 129 studies for title and abstract screening, of which three were retrieved.^94–96^

Reference and citation searching of included studies and selected reviews and guidelines identified another 46 studies^7,8,39,97–139^ for full retrieval and three additional systematic reviews^17,18,140^ for reference and citation searches.

In total, 118 primary studies and five systematic reviews were retrieved.

#### Study selection

Forty-nine primary studies met the inclusion criteria.^6–9,27,28,30,32,37,41,42,52,54,55,57,59,60,62,63,65,66,69,71,73–78,86,87,90,93,97–104,106–109,114,125,130,139^ One review presented new study data.^18^ The four remaining reviews formed part of the narrative synthesis.^17,19,20,140^ The reasons for excluding the remaining 69 studies are presented in Supplementary Table 3 (see online supplementary material at http://www.liebertpub.com). Anonymized individual patient data were provided by the authors of a cohort study to allow outcomes for initial GCS13-15 patients to be calculated, so this study is included.^139^

#### Study characteristics

Supplementary Table 4 presents the characteristics of included studies (see online supplementary material at http://www.liebertpub.com). Seven prospective studies were identified^28,66,74,75,90,114,139^ and four studies had a sample size of over 1000.^63,87,98,108^ Forty-six studies estimated the outcomes of interest and contributed to pooled estimates of risk.^6–9,27,28,30,32,37,41,42,52,54,55,57,59,60,62,63,65,66,69,71,73–78,86,87,90,93,97–104,106–109,114,125,130,139^ Four studies presented data regarding specific injury subtypes.^32,55,71,103^ One study only contributed to the narrative synthesis due to the outcome measure it assessed.^42^ Three studies presented the Brain Injury Guidelines (BIG) risk stratification tool.^9,27,109^ As this tool was applied to all TBI patients and initial GCS forms part of risk stratification, these studies contributed to the narrative synthesis.

Twenty-one studies presented either uni-variate or multi-variable analysis assessing prognostic factors' effect on the outcomes of interest.^6,37,41,54,55,66,69,71,73–78,87,98–101,130,139^ Sixteen studies presented multi-variable models using logistic regression or recursive partitioning.^6,37,41,54,55,66,69,71,73,74,77,78,98,100,101,130^ Only two studies attempted to validate such models by splitting the study datasets.^66,98^

#### Quality assessment

QUIPS quality scores are presented in Supplementary Table 2.^21^ The following common methodological issues were identified.

Study recruitment was often not representative of all GCS 13–15 patients with TBI identified by CT imaging. Sixteen studies that contributed to the pooled estimates of adverse outcomes only included patients who had undergone repeat CT imaging, and so are likely to represent a higher-risk population.^7,18,54,74–78,86,90,102,104,106,107,125,130^ Even when re-imaging was presented as routine practice, it was often indicated that not all patients were re-imaged and included in analysis.^6^ Many other studies excluded higher-risk anti-coagulated patients or those with more severe injuries.

Prognostic factor measurement was not consistent. Continuous variables were dichotomized at different thresholds or the same risk factor was measured with different methods. For example, the severity of injury identified by CT imaging was assessed with 10 different measures. Most studies were retrospective and reliant on the accuracy of case notes and radiological reports. The small sample size of many studies prevented multi-variable modeling with all variables identified in uni-variable modeling as affecting deterioration.^37^

In 32 studies, outcomes were assessed during inpatient admission, and so patients who were discharged and deteriorated were missed. In other studies, is wasn't clear when outcome measures were assessed. Eight different measures of clinical deterioration were used in 18 studies.

Several studies included patients with extra-cranial injuries and significant co-morbidities. Extra-cranial injuries caused clinical interventions, and in studies that measured deterioration in this way this was a potential source of bias.^66^ Other studies indicated some recorded deaths were related to co-morbidities instead of TBI.^41,73^

### Risk of adverse outcomes and exploration of between-study variation

#### Death

**FIG. 2.**
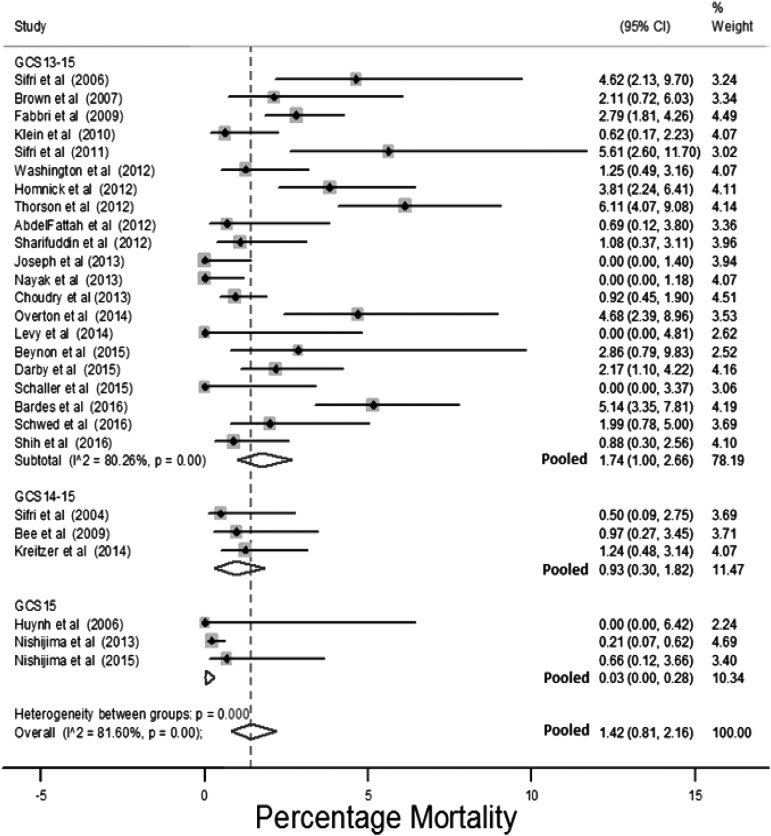
Risk for death stratified by initial Glasgow Coma Scale (GCS).

Twenty-seven studies assessed the outcome of death.^6,8,28,41,52,57,60,62,63,65,69,73–75,78,86,93,97,99–102,104,114,125,130,139^ The estimated risk of death for these studies ranged between 0–6% (median 1.1%), and with a pooled prevalence of 1.4% (95% confidence interval [CI]: 0.8%–2.2%; Fig. 2). Studies that selected only initial GCS15 patients had a pooled estimate of mortality of 0.03% (95% CI: 0%–0.28%). Studies that selected populations for non-intensive care unit (non-ICU) admission or other conservative care pathways had an estimated prevalence of death of 0.1% (95% CI: 0%–0.6%).

**FIG. 3.**
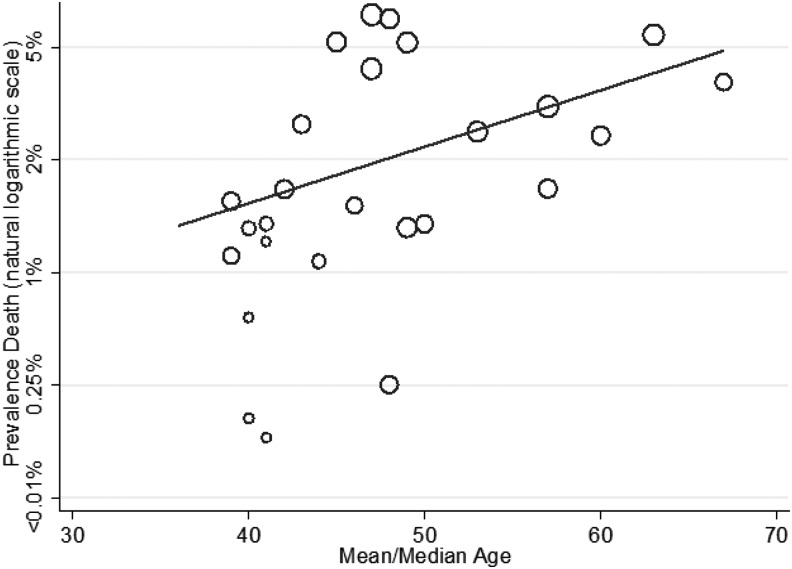
Meta-regression of risk for death by mean age study population (coefficient odds 1.05, 95% confidence interval [CI]: 1.00–1.12; *p* = 0.049).

**FIG. 4.**
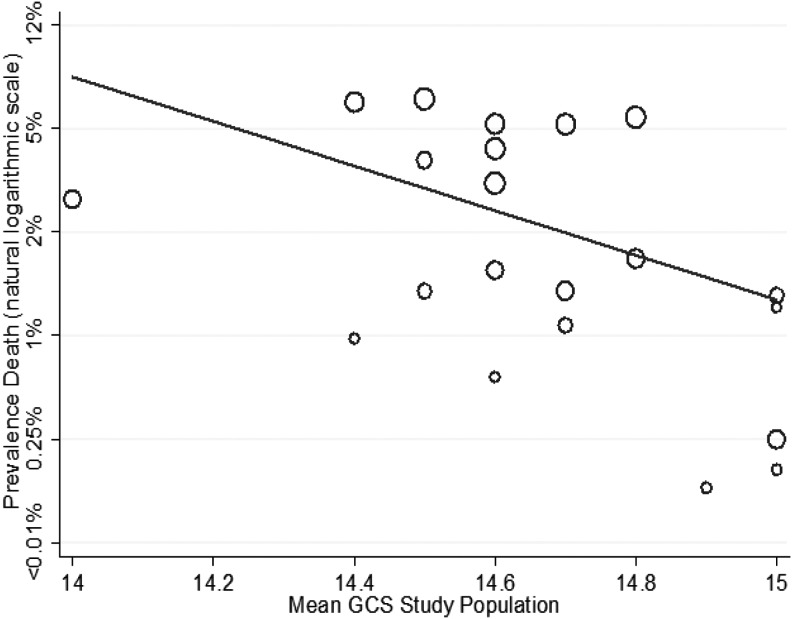
Meta-regression of risk for death by mean Glasgow Coma Scale (GCS) study population (coefficient odds 0.12, 95% confidence interval [CI]: 0.02–0.86; *p* = 0.04).

The effect on mortality of mean GCS, average age, and selection of study population for a lower level of care was explored using meta-regression. Increased age of study population was associated with a higher risk for death (1.05, 95% CI: 1.00–1.12; Fig. 3), whereas higher study population GCS was associated with a lower risk for death (0.12, 95% CI: 0.02–0.86; Fig. 4). The percentage of patients taking anti-coagulants in studies was not associated with the prevalence of death (1.05, 95% CI: 0.95–1.17), but selection for a lower level of care compared with a higher level of care was (0.27, 95C.I: 0.08–0.94). When average age of the study population and mean study GCS were assessed in a multi-variable model they remained statistically significant predictors of mortality (Table 1), with an adjusted R squared of 38%, indicating that these two factors explained over one-third of the variation in study estimates.

**Table 1.** Meta-regression of Study Factors Predictive for Death, Neurosurgery, and Clinical Deterioration

*Factor*	*Outcome*	*Unit increase affect odds uni-variable model*	*Unit increase affect odds multi-variable model*
Mean age study population	Death	1.05 (95% CI: 1.0003–1.12), *p* = 0.049	1.06 (95% CI: 1.0002–1.12), *p* = 0.049
Mean GCS study population	Death	0.12 (95% CI: 0.02–0.86), *p* = 0.04	0.09 (95% CI: 0.01–0.59), *p* = 0.02
Lower-risk study population vs. ICU population	Death	0.27 (95% CI: 0.08–0.94), *p* = 0.04	
Unselected study population vs. ICU population	Death	0.81 (95% CI: 0.22–1.97), *p* = 0.63	
Percentage population anti-coagulated	Death	1.05 (95% CI: 0.95–1.17), *p* = 0.32	
Mean age study population	Neurosurgery	1.01 (95% CI: 1.02–1.11), *p* = 0.01	1.09 (95% CI: 1.02–1.16), *p* = 0.02
Mean GCS study population	Neurosurgery	0.71 (95% CI: 0.01–0.56), *p* = 0.01	0.12 (95% CI: 0.02–0.91), *p* = 0.04
Lower-risk study population vs. ICU population	Neurosurgery	0.13 (95% CI: 0.04–0.41), *p* < 0.01	0.67 (95% CI: 0.10–4.37), *p* = 0.66
Unselected study population vs. ICU population	Neurosurgery	0.95 (95% CI: 0.43–2.12), *p* = 0.90	1.34 (95% CI: 0.45–4.02), *p* = 0.58
Percentage population anti-coagulated	Neurosurgery	1.1 (95% CI: 1.01–1.19), *p* = 0.04	
Exclusion of anti-coagulated patients in study selection	Neurosurgery	0.63 (95% CI: 0.27–1.43), *p* = 0.26	1.33 (95% CI: 0.51–3.49), *p* = 0.54
Mean age study population	Clinical deterioration	1.01 (95% CI: 0.95–1.09), *p* = 0.64	1.02 (95% CI: 0.93–1.12), *p* = 0.59
Mean GCS study population	Clinical deterioration	0.36 (95% CI: 0.04–3.20), *p* = 0.33	0.26 (95% CI: 0.02–3.76), *p* = 0.29

CI, confidence interval; ICU, intensive care unit; GCS, Glasgow Coma Scale.

#### Neurosurgical intervention

**FIG. 5.**
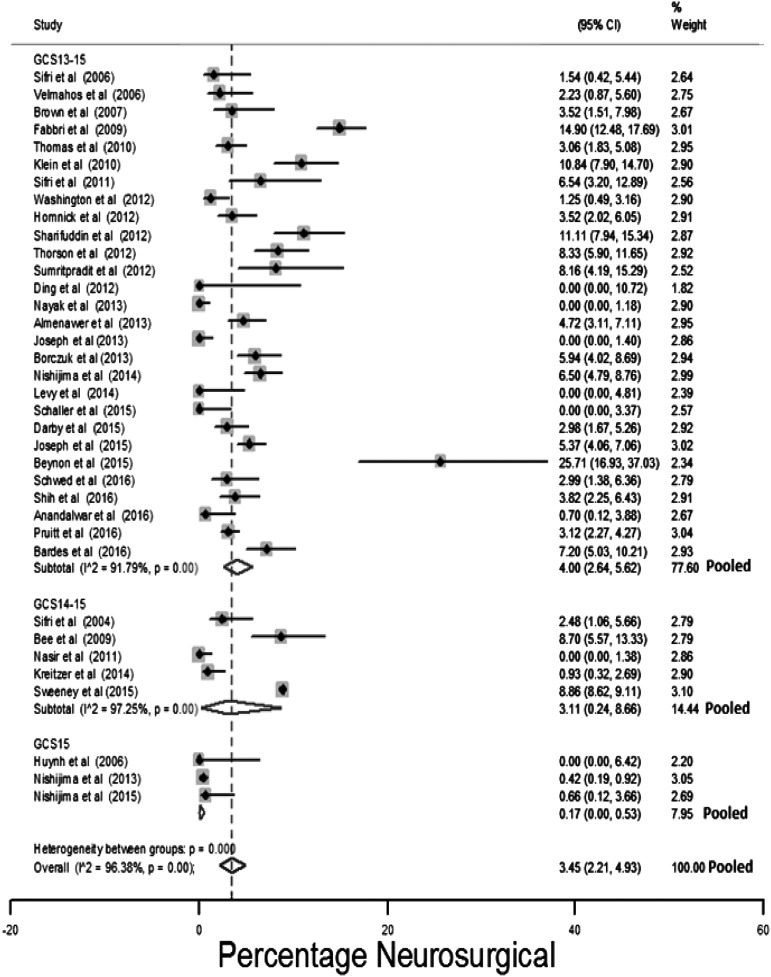
Risk for neurosurgery stratified by the initial Glasgow Coma Scale (GCS) of the study population.

Thirty-six studies reported neurosurgical outcomes.^6–9,27,30,37,52,54,57,60,62,63,65,66,73–78,86,90,93,97–102,104,106,109,114,125,130,139^ Figure 5 presents the estimates of the proportion of patients who underwent a neurosurgical procedure stratified by the GCS inclusion criteria. Reported neurosurgical intervention prevalence ranged between 0 and 26% (median 3.1%). The high proportion requiring neurosurgical intervention reported by Beynon and associates^93^ may reflect the greater use of anti-coagulants or anti-platelets (33/70 participants).

The pooled estimated neurosurgical intervention risk was 3.5% (95% CI: 2.2%–4.9%). An I^2^ of 96.4% indicated considerable heterogeneity. Studies conducted on initial GCS 15 patients had a lower prevalence of neurosurgical intervention: 0.2% (95% CI: 0%–0.5%). Sensitivity analysis of selection of the study population for reduced care, such as discharge, a non-ICU admission or non-routine repeat CT imaging found the pooled estimate of neurosurgical intervention in these studies to be 0.1% (95% CI: 0%–0.5%).

**FIG. 6.**
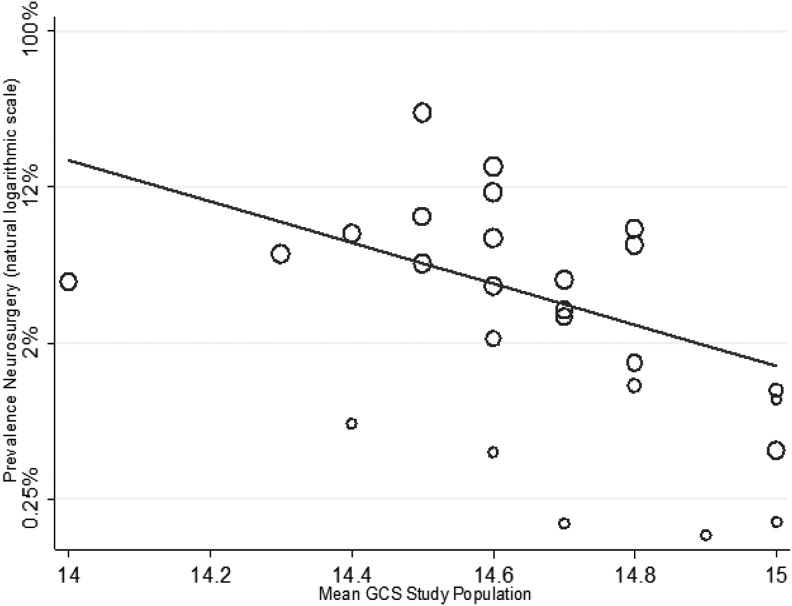
Meta-regression of risk for neurosurgery by mean Glasgow Coma Scale (GCS) study population (coefficient odds 0.71, 95% confidence interval [CI]: 0.01–0.56; *p* = 0.01).

**FIG. 7.**
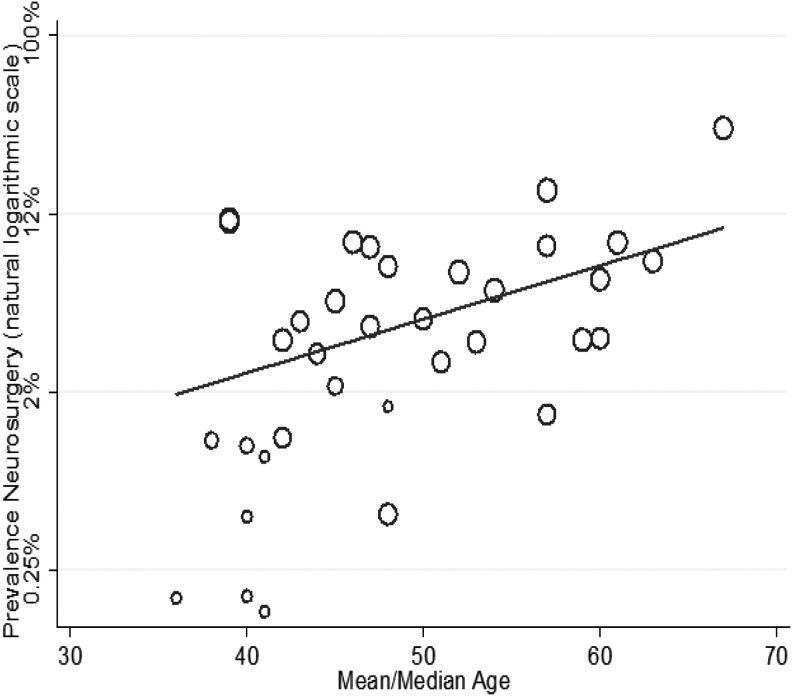
Meta-regression of risk for neurosurgery by mean age study population (coefficient odds 1.01, 95% confidence interval [CI]: 1.02–1.11; *p* = 0.01).

**FIG. 8.**
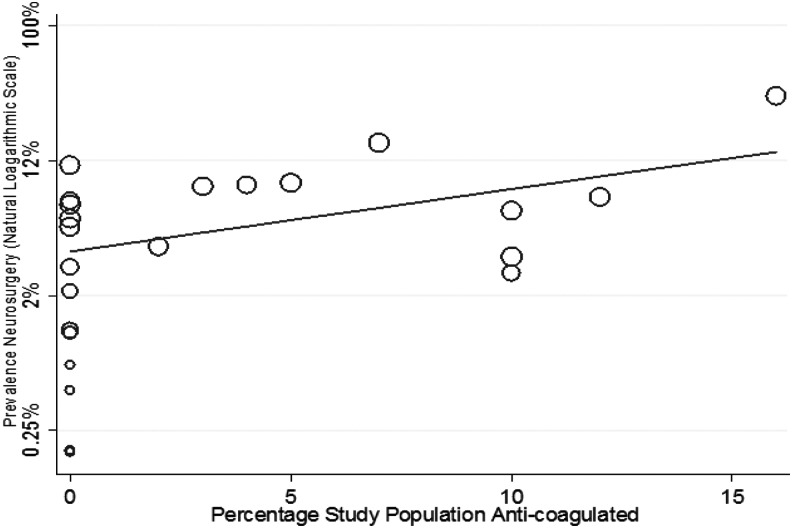
Meta-regression of risk for neurosurgery by percentage of study population taking anti-coagulants (coefficient odds 1.1, 95% confidence interval [CI]: 1.01–1.19; *p* = 0.04).

The of result of meta-regression using: mean study population GCS, mean study population age, anti-coagulation, and selection of study population for non-ICU admission or other reduced care pathways is shown in Figures 6–8 and Table 1. Increasing age (1.01, 95% CI: 1.02–1.11) and increasing percentage of study population taking anti-coagulants (1.1, 95% CI: 1.01–1.19) was associated with a higher risk, whereas an increasing GCS (0.71, 95% CI:0.01–0.56) was associated with a lower risk, of neurosurgical intervention.

Figure 7 shows a cluster of four small studies with low mean ages that appear to have a disproportionately low estimated prevalence of neurosurgical intervention.^8,52,62,106^ This is explained by: exclusion of anti-coagulated patients,^8,52,62^ selection of patients for non-ICU admission or other reduced other care pathays,^8,52,62^ and exclusion of patients with large injuries.^8^

When the effect of population selection for reduced clinical management, exclusion of anti-coagulated patients (only 23/36 studies reported percentage of anti-coagulated patients), mean age, and GCS of the study population were all included in a meta-regression, age and GCS were the only statistically significant predictors of neurosurgical intervention (Table 1). The adjusted R squared of the model was 48%, indicating that these factors accounted for almost half of between-study variation.

#### Clinical deterioration

**FIG. 9.**
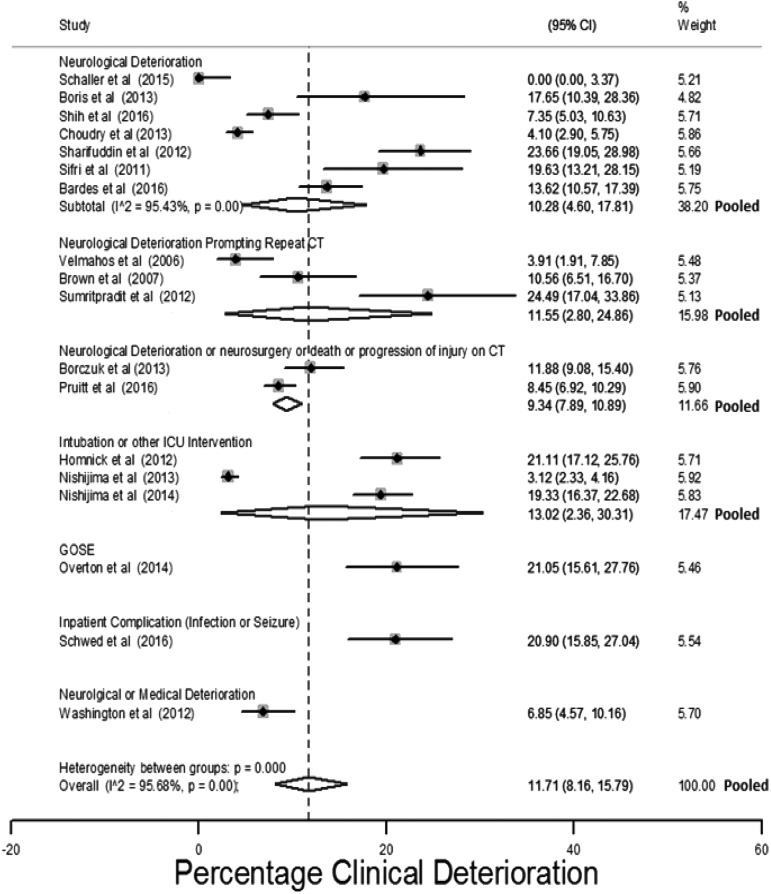
Estimates of clinical deterioration stratified by the outcome measure.

Eighteen studies measured prevalence of clinical deterioration.^8,37,41,63,66,69,73,74,76–78,100,101,104,107,108,114,125^ The estimated risk of deterioration ranged between 0 and 24.5% (median 12.8%). Figure 9 presents study estimates of the percentage of patients who deteriorated, with 95% CIs and stratified by how the outcome was assessed. A pooled prevalence of 11.7% (95% CI: 8.21%–5.8%) for some form of clinical deterioration was estimated with an I^2^ of 95.7%.

Estimates were stratified by: initial GCS of patients, whether the included population were all selected for repeat CT imaging, the inclusion of anti-coagulated patients, the follow-up period, and exclusion of patients with extra-cranial injuries. None of these factors reduced the observed between-study heterogeneity.

The effect of: mean GCS study population, mean age study population, study population selection, exclusion of patients with extra-cranial injuries, and exclusion of anti-coagulated patients was explored using meta-regression. As only 18 studies measured this outcome, the model was restricted to two variables. No factor assessed individually or in conjunction with another factor was found to statistically affect the risk of clinical deterioration. Higher age and lower GCS were non-statistically associated with a higher risk of clinical deterioration (Table 1).

#### Progression on repeat CT imaging

**FIG. 10.**
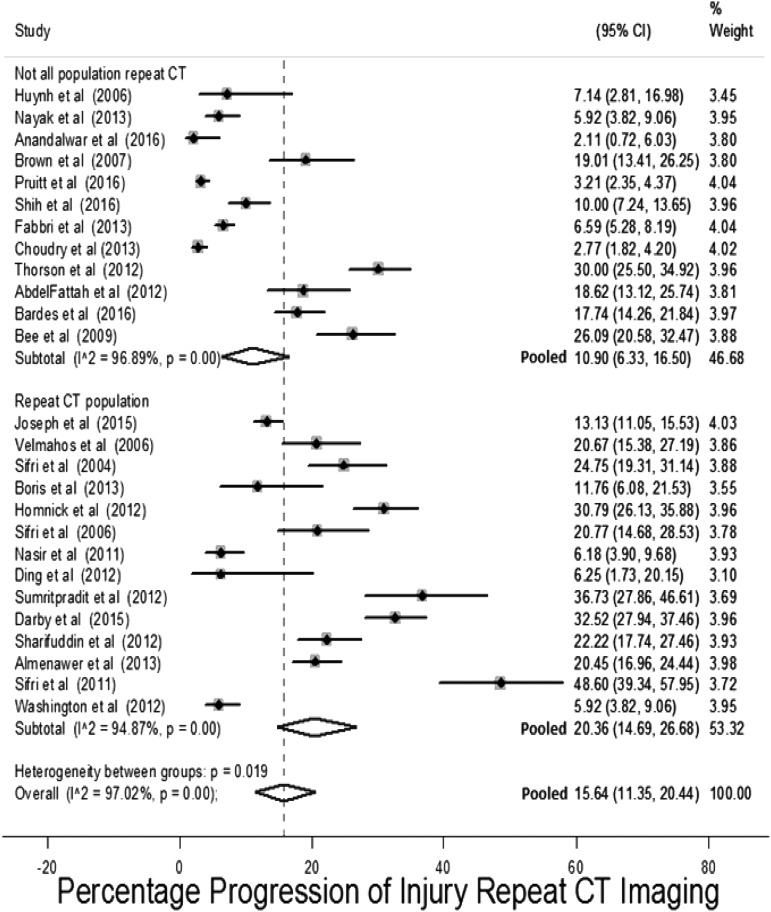
Risk on repeat computed tomography (CT) imaging for progression of injury stratified by whether entire population selected for repeat imaging.

Twenty-six studies assessed the outcome progression of the initial injury on repeat CT imaging.^6,18,27,28,30,41,62,74–78,87,90,97,99–102,104,106–108,114,125,130^ The prevalence of this outcome in these studies is presented in Figure 10, stratified by whether studies only included patients who had undergone repeat CT imaging. The pooled estimate for this outcome was 15.6% (95% CI: 11.3%–20.4%). There is a high degree of heterogeneity with a range in risk of progression between 2 and 48% (median 36.5%) and I^2^ = 97%. The non-statistically significant higher pooled risk in studies that included only patients who had undergone repeat CT imaging probably reflects selection of higher-risk patients to repeat imaging. Subgroup analysis of study characteristics did not find any factors that accounted for the heterogeneity. This is probably the result of different criteria used to triage patients to repeat CT imaging and definition of progression of injury.

### Prognostic factors assessed in primary studies

Twenty-one studies presented within-study estimates of effect of individual risk factors on the outcomes of interest (Supplementary Table 4) and the factors assessed are presented in Supplementary Table 5 (see online supplementary material at http://www.liebertpub.com).^6,37,41,54,55,66,69,71,73–78,87,98–101,130,139^ The most influential factors were: age, initial GCS, severity of CT finding, type of injury, anti-coagulation, and anti-platelet medication (Table 2). Individual forest plots are presented in Supplementary Table 6.

**Table 2.** Summary of Effect Estimates of Risk Factors Assessed within Studies

*Risk factor*	*Number of studies assessed in*	*Pooled uni-variable effect^a^*	*Effect multi-variable models^b^*	*Likely effect on risk*
Age	18^6,37,41,54,55,66,69,71,73,74,76–78,98–101,130^		+6/11	**+**
Initial GCS 15	7^37,41,66,73,74,77,101^	OR 0.35, 95% CI: 0.23–0.52	−4/4	**−**
Severity CT brain	9^6,41,54,55,66,73,76,78,100^		+7/8	**+**
Isolated SAH	5^37,73,77,98,108^	OR 0.19, 95% CI: 0.07–0.5	−1/2	**−**
Isolated EDH	5^37,73,77,98,108^	OR 2.26, 95% CI: 1.9–2.68	+1/1	**+**
Isolated SDH	5^37,73,77,98,108^	OR 1.82, 95% CI: 0.69–4.77	+2/2	
Isolated contusion	3^37,98,108^	OR 0.24, 95% CI: 0.2–0.28	0/1	
Anti-coagulation	12^6,37,41,55,74,76–78,98,100,101,139^	OR 1.45, 95% CI: 1.28–1.64	0/2	**+**
Aspirin	6^37,55,66,76,87,101^	OR 1.30, 95% CI: 0.95–1.78		
Clopidogrel	6^37,55,66,76,87,101^	OR 1.79, 95% CI: 1.17–2.72		**+**

^a^ Pooled estimate of effect on risk of neurosurgery or clinical deterioration.

^b^ Indicates number of multi-variable models where factor was found to be a significant predictor and direction of effect on risk.

CI, confidence interval; CT, computed tomography; EDH, extra-dural hemorrhage; GCS, Glasgow Coma Scale; OR, odds ratio; SAH, subarachnoid hemorrhage.

#### Age

Age was evaluated as a factor in prognostic modeling in 18 primary studies.^6,37,41,54,55,66,69,71,73,74,76–78,98–101,130^ Ten studies^37,41,54,66,73,74,76–78,101^ assessed age using four different dichotomous cutoffs and 11 studies measured age as a continuous factor.^6,55,69,71,73,76,77,98–100,130^ Multi-variable models included: logistic regression with age either a dichotomized or continuous variable, or decision tree analysis.

Of these 18 studies: six assessed the outcome of clinical deterioration, eight assessed the outcome of neurosurgical intervention, one measured death as an outcome, and eight studies evaluated progression of injury on repeat CT imaging. Despite being the most commonly assessed prognostic factor, due to the variation in measurement and the outcomes assessed, it was not possible to undertake a pooled analysis.

Increased age was associated with an adverse outcome in 9 of the 19 uni-variable models presented. Age was a significant predictor of an adverse outcome in 2 of 5 multi-variable models where it was treated as a continuous variable.^69,71,98,130^ However, in 4 of 6 multi-variable models where it was dichotomized, older age predicted the outcomes of interest.^41,54,66,73,78,101^ This may indicate a non-linear relationship with older age groups having a disproportionately higher associated risk for adverse outcomes.

#### Initial GCS

Twelve primary studies presented within-study estimates of the effect of initial GCS on the risk of the outcomes of interest.^6,37,41,55,66,69,73,74,77,98,100,101^ Uni-variable effect estimates of initial GCS of 15 were pooled for studies assessing clinical deterioration and neurosurgical intervention as an outcome with individual patient data provided by Fabbri and co-workers, and an initial GCS of 15 was protective against clinical deterioration or neurosurgical intervention (pooled odds ratio [OR] 0.35, 95% CI: 0.23–0.53; Table 2).^37,41,66,73,74,77,101^ Two articles assessed progression of injury on repeat CT imaging and both found an initial GCS of 15 to be associated with reduced risk of progression.^74,77^ Four studies estimated the effect of an initial GCS of 15 in multi-variable models.^37,66,73,101^ All four multi-variable models found initial GCS of 15 to be associated with a reduced risk of adverse outcomes.

#### Severity of injury as assessed by CT findings

Nine studies estimated whether the severity of injury identified by initial CT scan predicted adverse outcomes.^6,41,54,55,66,73,76,78,100^ This was assessed by: the presence of midline shift or mass effect in five studies,^6,55,66,76,100^ the Marshall classification in two studies,^41,73^ and measures of hemorrhage thickness or volume in four studies.^54,55,78,100^ The variability in the measures of injury severity and differences in the outcomes assessed prevented pooling.

All studies that assessed presence of midline shift/mass effect found it to be statistically predictive of adverse outcomes. This association remained in the two studies that presented multi-variable analysis.^6,66^ The Marshall classification was assessed as a continuous^73^ and dichotomized variable,^41^ and neither study found a statistically significant association with adverse outcomes.

The two studies that assessed the effect of bleed thickness >10 mm found this to be statistically predictive of either progression of injury on repeat CT imaging or neurosurgical intervention in both uni- and multi-variable analysis.^54,78^

#### Isolated subarachnoid hemorrhage

**FIG. 11.**
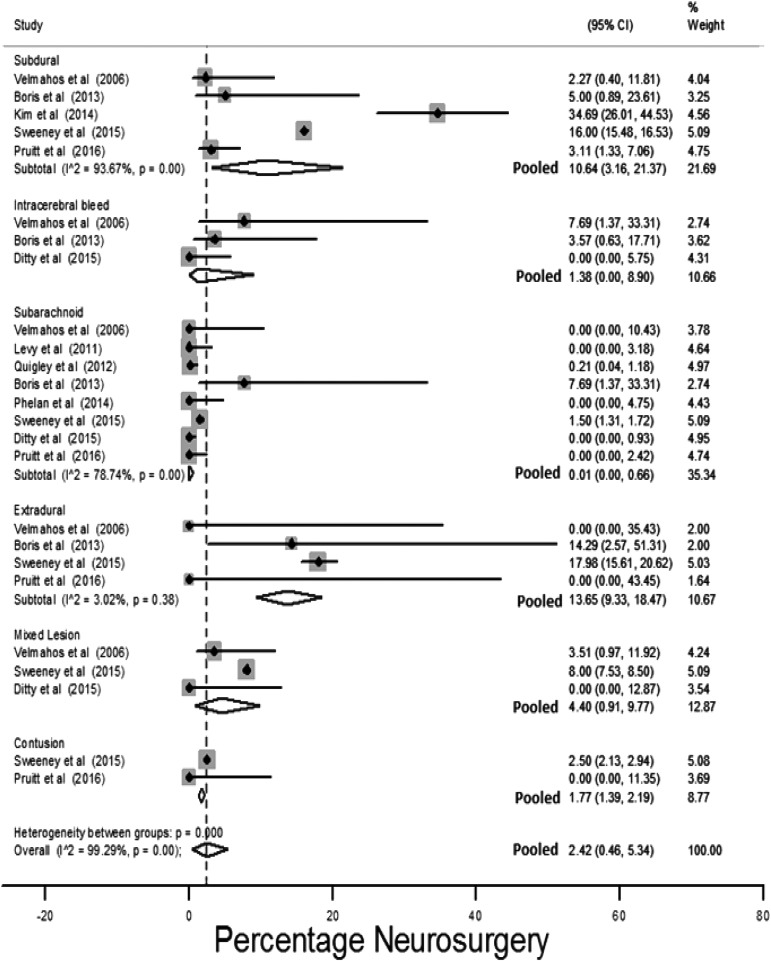
Pooled risk for neurosurgery stratified by isolated injury type identified by initial computed tomography (CT) imaging.

Twelve studies presented outcomes for populations with isolated injuries and patients with isolated subarachnoid hemorrhages (iSAH) had the lowest risk for adverse outcomes: neurosurgical intervention pooled risk 0.01% (95% CI: 0%–0.7%; Fig. 11), and 1.1% (95% CI: 0%–5.5%) pooled prevalence of clinical deterioration (Supplementary Fig. 1; (see online supplementary material at http://www.liebertpub.com).^32,37,55,59,71,74,77,98,99,103,107,108^

Uni-variable effect estimates presented in the two studies that assessed the effect of the presence of iSAH were pooled with data extracted from three additional studies.^37,73,77,98,108^ The pooled estimate indicated iSAH reduced the risk of neurosurgical intervention/clinical deterioration (Table 2).

Two multi-variable models included iSAH as a prognostic factor. One found iSAH to be associated with a lower risk for clinical deterioration.^37^ The other found iSAH to have no effect on risk.^98^

#### Isolated extra-dural hemorrhage

Patients with isolated extra-dural hemorrhage had the highest risk for neurosurgical intervention: 13.7% (95% CI: 9.3%–18.5%; Fig. 11); 18.5% is estimated from a population of all initial GCS14–15 patients with extra-dural hemorrhage, whereas the estimates in the other studies are from populations that have been selected for more conservative management.^77,98,107,108^

Three studies assessed isolated extra-dural hmorrhage as a prognostic factor.^37,73,98^ A pooled risk estimate for clinical deterioration or neurosurgical intervention using these three studies and outcome data extracted from a further two studies,^77,108^ found isolated extra-dural hemorrhage to be associated with these outcomes (OR 2.26, 95% CI: 1.9–2.68; Table 2). Isolated extra-dural hemorrhage remained statistically associated with neurosurgical outcomes in the only multi-variable model that included this factor.^98^

#### Anti-coagulation

Twelve studies estimated the prognostic effect of anti-coagulation.^6,37,41,55,74,76–78,98,100,101,139^ Measures of anti-coagulation included: any documented coagulopathy,^6,41,55,77,98,100^ pre-injury warfarin use,^37,76,101^ warfarin or anti-platelet therapy as a combined risk factor,^78,100^ and continuous laboratory measures of anti-coagulation.^6,74,101^

Uni-variable effect estimates of dichotomous measures of anti-coagulation were pooled with individual patient data from Fabbri and colleagues for the composite outcome of clinical deterioration or neurosurgical intervention (Table 2), pooled estimate: OR 1.45, 95% CI: 1.28–1.64.

Two studies presented multi-variable models that included anti-coagulation and it was not statistically associated with the outcomes of interest in either model.^78,98^

#### Anti-platelet medication

The effect of anti-platelet use was evaluated by: aspirin use,^37,76,101^ clopidogrel use,^37,76,101^ and a joint measure of anti-platelet use.^55,66,87^ No multi-variable models included anti-platelet use. Pooled uni-variable risk estimates of pre-injury aspirin and clopidogrel use are presented in Table 2. Meta-analysis indicated a statistical association between clopidogrel and clinical deterioration or neurosurgical intervention, but there was no association between aspirin use and this outcome.

## Discussion

### Summary

We have completed a thorough systematic review and meta-analysis to identify risk factors for adverse outcomes in this TBI population. This is the first review to provide pooled estimates of clinically important outcomes in this population and identify which factors affect the risk for these outcomes.

The pooled prevalence for adverse outcomes was: 11.7% (95% CI: 8.21%–5.8%) clinical deterioration, 3.5% (95% CI: 2.2%–4.9%) neurosurgical intervention, and 1.4% (95% CI: 0.8%–2.2%) death. These outcome estimates used a pooled total of 65,724 patients and are comparable to the 2.7% craniotomy rate reported for a similar population in a national UK trauma database.^94^ The variation in individual study outcomes reflects differences in populations studied and outcome definitions. For the outcomes of neurosurgical intervention and death, heterogeneity could be explained by the age of study populations and different study population GCS scores.

Risk factors for adverse outcomes were identified using both meta-regression of study characteristics and synthesis of prognostic models presented by primary studies. Age, anti-coagulation, and initial GCS were found by both methods to affect risk. An increase in mean study population age by one year was associated with increased odds of neurosurgical intervention of 1.09 in multi-variable meta-regression (Table 1), and age was a predictor for an adverse outcome in 6 of 11 multi-variable models presented in primary studies. In uni-variable meta-regression a unit increase in the percentage of the study population taking anti-coagulants was associated with a 1.1 increase in the odds of neurosurgical intervention (Table 1). Pooling of uni-variable models presented in primary studies found anti-coagulated patients to have odds 1.45 times greater than patients not anti-coagulated for neurosurgical intervention/clinical deterioration (Table 2). In multi-variable meta-regression, a unit increase in mean/median study population GCS was associated with a 0.12 reduction in the odds of neurosurgical intervention (Table 1). Pooling of uni-variable models indicated that patients with an initial GCS <15 had odds of clinical deterioration/neurosurgical intervention 2.9 times that of patients who presented with an initial GCS of 15 (Table 2). In multi-variable meta-regression models including both initial GCS and age, initial GCS had a smaller effect on the risk for either neurosurgical intervention or death than in uni-variable analysis, and this may be due to older patients presenting with higher initial GCS relative to the severity of their injury (Table 1).^141^ Patients with extra-dural hemorrhage had the highest prevalence of adverse outcomes, whereas patients with iSAH had the lowest (Fig. 11).

Meta-analysis of multi-variable models was not possible due to the small number and variability in how these models were constructed. Therefore, although this review has identified the factors that affect risk, no model that could identify low-risk patients was found or could be reliably constructed.

### Strengths

A thorough search has been conducted, identifying 50 relevant primary studies. Our review fulfills all the AMSTAR systematic review checklist quality domains apart from items 10 and 11, regarding the assessment of publication bias and conflicts of interest.^142^ However, the non-interventional nature of the included studies means these domains are less relevant. This review is low-risk for bias in the five domains assessed by the Risk of Bias in Systematic reviews (ROBIS) tool.^143^

### Limitations

Many studies identified were small and retrospective with limited follow-up of patients after discharge. Instead of attempting to identify low-risk patients through prognostic modeling, several studies selected patients on study-specific characteristics for different care pathways. This variation in study populations contributed to heterogeneity in estimates of outcome prevalence and risk-factor effect. The prognostic models that were identified were often derived in cohorts too small to construct multi-variable models with all relevant factors. The clinically useful outcome in informing discharge decisions is clinical deterioration, and most prognostic models did not assess this.

Clinical deterioration was defined by seven different composite outcomes and most commonly by neurological deterioration. This lack of consistency in definition contributed to the heterogeneity in outcome estimates. Neurological deterioration was variably defined and a clinically relevant and consistently used definition or deterioration is required.

No included studies assessed pupillary response and duration of loss of consciousness/amnesia. These factors are predictive for adverse outcomes in other TBI populations and future research should assess these factors in this population.^13,144^

### Context

When the Canadian CT Head Rule was developed, the authors presented a consensus-derived list of intra-cranial injuries that would never require neurosurgical intervention.^4^ The implication was that patients with such injuries were safe for discharge. This was rejected by the Society of British Neurological Surgeons.^1^ A U.S. group based in Arizona has produced the BIG consensus-derived statement that identifies a population with low-risk clinical characteristics and intra-cranial injuries similar to those presented by the CCHR authors.^109^ They propose such patients are safe for discharge after 6 h of ED observation.^9,27,109^

Kreitzer and associates present an alternative policy at a level 1 trauma center in Cincinnati, where the population of interest remains in the ED for observation and undergoes repeat CT imaging approximately 6 h following diagnosis.^86^ Neurologically stable patients without progression of injury are discharged. Pruitt and co-workers present a model of care in a level 1 trauma center in Chicago in which all GCS13–15 patients with intra-cranial injuries receive a neurosurgical consultation.^108^ Low-risk patients identified by the neurosurgeon are left under ED care and discharged after a period of observation. This is similar to the standard of care in the UK National Health Service (NHS).

Others advocate the admission of GCS13–15 patients with brain injuries identified by CT imaging to higher levels of care and routine re-imaging, citing evidence that deterioration in neurological examination may not identify progression of injury that warrants clinical intervention.^6,78^ Multiple reviews have found that this is too rare an occurrence to warrant routine re-imaging of all GCS13–15 patients with TBI identified by CT.^17–20^

### Implications

This review supports the view that there are subsets of GCS13–15 patients with injuries identified by CT imaging who may possibly be safely routinely discharged from the ED. However, the current available evidence is insufficient to reliably identify such low-risk patients. The risks for serious adverse outcomes are sufficiently high that, in the absence of evidence to be able to accurately pinpoint low-risk individual patients, admission for observation probably remains clinically indicated.

No validated model predicting a measure of clinical deterioration that could be used to triage hospital admission was identified. We suggest future research should assess a measure of clinical deterioration that encompasses: neurosurgical intervention, death, a fall in GCS by 2 or more points, seizure activity, intravenous medical intervention, or ICU intervention. These would warrant ongoing inpatient hospital admission.

The BIG criteria, although the best effort at risk stratifying this group in a clinically relevant way, require validation in larger prospective cohorts in different health care contexts before being more widely adopted. They were derived by consensus, and empirical prognostic modeling could possibly improve the accuracy of risk stratification.

Decision rules have been employed successfully in the ED to risk-stratify patients in a range of conditions, including ankle injuries and suspected pulmonary embolus.^145,146^ Equivalent models could be used for patients with mild TBI to identify low-risk patients. This review has identified the key factors that are likely to inform such risk stratification, but an adequately powered derivation study with a clinically relevant definition of deterioration and adequate follow-up is required.

## Conclusion

Mild TBI patients with injuries identified by CT imaging are a heterogenous group. Their overall risk for clinical deterioration and more serious adverse outcomes is small, but clinically significant. Current research gives an indication to which factors affect the risk for adverse outcomes but is of too low quality to inform clinical decision-making. High-quality prognostic modeling is needed to help inform discharge decisions.

## Supplementary Material

Supplemental data
