# Supplementary material for: The Risk of Deterioration in GCS13–15 Patients with Traumatic Brain Injury Identified by Computed Tomography Imaging: A Systematic Review and Meta-Analysis
Source: J Neurotrauma. 2018 Mar 1;35(5):703–18. doi: 10.1089/neu.2017.5259 (PMC5831640; doi:10.1089/neu.2017.5259)

Supplementary Table 1. Full Search Strategy

*EMBASE (via OVID) search 11/24/2016: 1996 to 2016, week 47*

|  |  |  |  |
| --- | --- | --- | --- |
| 12 | | 1 and 10 and 11 | 3167 |
| 11 | | 2 or 3 or 4 or 5 or 6 or 9 | 104649 |
| 10 | | 7 or 8 | 2298555 |
| 9 | | "cerebral contusion".mp. or exp brain contusion/ | 2627 |
| 8 | | exp outcome variable/ or outcome.mp. or exp critical care outcome/ or exp adverse outcome/ | 1787765 |
| 7 | | exp prognosis/ or prognos*.mp. | 704898 |
| 6 | | exp subarachnoid hemorrhage/ or "traumatic subarachnoid h#em*".mp. | 28977 |
| 5 | | "extradural h#em*".mp. | 225 |
| 4 | | exp epidural hematoma/ or "epidural h#em*".mp. | 4775 |
| 3 | | exp subdural hematoma/ or "subdural h#em*".mp. | 10281 |
| 2 | | exp Intracranial Hemorrhages/ or "intracranial h#em*".mp. | 92720 |
| 1 | | "traumatic brain injury".mp. or traumatic brain injury/ or head injury/ | 69888 |

*MEDLINE (R) (via OVID) without revisions search 11/24/2016: 1996 to November, week 3, 2016*

|  |  |  |
| --- | --- | --- |
| 9 | 1 and 7 and 8 | 1143 |
| 8 | 2 or 3 or 4 or 5 or 6 | 34984 |
| 7 | exp Risk Factors/ or risk.mp. or exp Risk/ or exp Risk Assessment/ | 1502469 |
| 6 | "traumatic subarachnoid h#emorrhage".mp. or exp Subarachnoid Hemorrhage, Traumatic/ | 231 |
| 5 | exp Cerebral Hemorrhage, Traumatic/ or exp Hematoma, Epidural, Cranial/ or "extradural haemorrhage".mp. | 1434 |
| 4 | exp Hematoma, Subdural/ or "subdural h#em*".mp. | 3712 |
| 3 | exp Intracranial Hemorrhages/ or "intracranial h#em*".mp. | 34253 |
| 2 | exp Cerebral Hemorrhage/ or "intracerebral h#em*".mp. | 14418 |
| 1 | "head injury".mp. or exp Craniocerebral Trauma/ | 75438 |
|  | |  |

*CINHAL plus (via EBSCO) search 11/24/2016: 1983 to 2016*

| *Search terms* | *Search options* |  |
| --- | --- | --- |
| S11 | ((S3 OR S4 OR S5 OR S6) AND (S3 OR S4 OR S5 OR S6 OR S7)) AND (S8 AND S9 AND S10) | [**View Results**](javascript:__doPostBack('ctl00$ctl00$FindField$FindField$historyControl$HistoryRepeater$ctl00$linkResults','')) (292) |
| S10 | (S3 OR S4 OR S5 OR S6) AND (S3 OR S4 OR S5 OR S6 OR S7) | [**View Results**](javascript:__doPostBack('ctl00$ctl00$FindField$FindField$historyControl$HistoryRepeater$ctl01$linkResults','')) (6995) |
| S9 | S1 OR S2 | [**View Results**](javascript:__doPostBack('ctl00$ctl00$FindField$FindField$historyControl$HistoryRepeater$ctl02$linkResults','')) (17,827) |
| S8 | prognosis or outcome | [**View Results**](javascript:__doPostBack('ctl00$ctl00$FindField$FindField$historyControl$HistoryRepeater$ctl03$linkResults','')) (592,464) |
| S7 | brain contusion OR cerebral contusion | [**View Results**](javascript:__doPostBack('ctl00$ctl00$FindField$FindField$historyControl$HistoryRepeater$ctl04$linkResults','')) (106) |
| S6 | extradural haematoma OR extradural hematoma OR (epidural hematoma or epidural hemorrhage) | [**View Results**](javascript:__doPostBack('ctl00$ctl00$FindField$FindField$historyControl$HistoryRepeater$ctl05$linkResults','')) (753) |
| S5 | intracerebral hemorrhage OR intracerebral haemorrhage OR intracerebral bleed | [**View Results**](javascript:__doPostBack('ctl00$ctl00$FindField$FindField$historyControl$HistoryRepeater$ctl06$linkResults','')) (2456) |
| S`4 | intracranial hemorrhage OR intracranial haemorrhage OR intracranial hematoma OR intracranial haematoma | [**View Results**](javascript:__doPostBack('ctl00$ctl00$FindField$FindField$historyControl$HistoryRepeater$ctl07$linkResults','')) (3176) |
| S3 | subdural hematoma OR subdural hemorrhage OR subdural haematoma OR subdural haemorrhage | [**View Results**](javascript:__doPostBack('ctl00$ctl00$FindField$FindField$historyControl$HistoryRepeater$ctl08$linkResults','')) (1246) |
| S2 | traumatic brain injury | [**View Results**](javascript:__doPostBack('ctl00$ctl00$FindField$FindField$historyControl$HistoryRepeater$ctl09$linkResults','')) (10,081) |
| S1 | head injury | [**View Results**](javascript:__doPostBack('ctl00$ctl00$FindField$FindField$historyControl$HistoryRepeater$ctl02$linkResults','')) (7746) |

*Cochrane CENTRAL*

*Search name Prognostic systematic review*

*Date run 11/24/16 11:33:55.251*

*ID Search Hits*

#1 Craniocerebral Trauma 417

#2 head injury 2563

#3 #1 or #2 2704

#4 Hematoma, Subdural 228

#5 Hematoma, Epidural, Cranial 20

#6 Cerebral Hemorrhage 2609

#7 Skull Fracture 130

#8 Skull Fracture, Basilar 6

#9 Skull Fracture, Depressed 13

#10 Brain Contusion 131

#11 #4 or #5 or #6 or #7 or #8 or #9 or #10 2969

#12 #3 and #11 211

All Results (211)


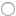
 Cochrane Reviews (138)


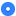
 All
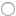
 Review
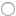
 Protocol


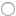
 Other Reviews (4)
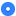
 Trials (63)
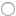
 Methods Studies (0)
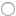
 Technology Assessments (0)
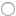
 Economic Evaluations (1)
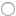
 Cochrane Groups (5)

Only trials retrieved.

| Supplementary Table 2. Data Extracted From Included Studies | | | | | | |
| --- | --- | --- | --- | --- | --- | --- |
| *Studies only included in meta-analysis of prevalence of outcomes (*n *= 26)* | | | | | | |
| *Reference* | *Population* | *Study design* | *Outcome measures* | *Prognostic factors assessed* | *Results* | *Quality appraisal* |
| Nishijima et al.  2013  Sacramento  USA  Variability of ICU use in adult patients with minor traumatic intra-cranial hemorrhages | Multi-center; 8 sites Western USA; all Level 1  Trauma registries searched for ICD-9 codes intra-cranial hemorrhage 2005-2010  Inclusion criteria:   - Age > 18 years - Traumatic ICH - Initial ED GCS 15 - ISS < 16 | Retrospective cohort study  Objective:  1) Assess the variability of ICU use in a cohort of patients with minor traumatic intra-cranial hemorrhages across multiple trauma centers.  2) Estimate the proportion of minor traumatic intra-cranial hemorrhages in patients admitted to ICU who do not receive an ICU intervention. | Initial ICU admission from ED  Proportion of patients receiving critical care intervention defined as:  Neurosurgical intervention  Mechanical ventilation  Vasopressor/ionotropic use  Transfusion blood product  Invasive monitoring | Age  Initial GCS  Initial BP  LOS hosp  ICU stay  Procedures as coded in trauma registry  AIS | 11,240 patients coded as bleeds.  771 excluded due to missing data.  1412 remaining met inclusion criteria.  888/1412 admitted to ICU, significant variation between sites  44/1412 (3.1%) had critical care intervention  6/1412 had neurosurgical intervention  847/888 patients admitted ICU, no critical care intervention  Mean/median GCS = 15  Mean/median age = 48 | **Study recruitment: Mod risk bias**  Dependent on accuracy on recording on trauma registry. Does have some quality assessment of data imputation  Note initial GCS15 - lower risk group  **Attrition: Low risk**  Follow-up only during hospital admission  **Prognostic factor measurement: Low risk**  Doesn’t really apply as testing disposition not outcomes  **Outcome measures: Low risk**  No measure of outcomes after discharge, but study primarily about disposition  Does not report deaths  **Confounding factors:**  States IIS increases ICU admission - will be related to other injuries  **Statistical techniques: Low risk**  N/A  **Overall**  Only GCS15 patients with low ISS |
| Nishijima et al.  2015  Sacramento  USA  Long-term neurological outcomes in adults with traumatic intra-cranial hemorrhage admitted to ICU versus floor | Level 1 trauma center  2008-2013  Inclusion criteria:   - Age >18 years - Identified ICH ICD-9 code trauma registry - Initial ED GCS 15 - Isolated head injury based on AIS score - Age <65 years - No evidence midline shift CT - Present on TBI database due to suspected TBI/evidence of ICH | Retrospective cohort study  Aim:  Compare long-term neurological outcomes in low-risk patients with traumatic intra-cranial hemorrhage (tICH) admitted to the ICU versus patients admitted to the floor. | Prospective long-term outcome measure at 6 months  Either GOS-E 8 fully recovered or GOS-E 1-7 not fully recovered | Age  Sex  Mechanism of injury  Initial ED GCS score  Initial (SBP)  Heart rate  Respiratory rate  Blood alcohol level  AIS score  ISS score  INR  Rotterdam CT score | 188 met inclusion criteria  151/188 complete data = cohort  106 admitted ICU (70%)  45 admitted ED (30%)  1/151 patients neurosurgical intervention as inpatient  1/151 patient died as inpatient  78 (52%) GOS-E 8 at 6 months  Does present analysis for outcome at 6 months GOS-E but no inpatient measures of deterioration  Adjusted analysis, floor admission versus ICU had an odds ratio of 0.77 (95% CI [0.36-1.64]) for a GOS-E score of 8 at six months.  Mean/median GCS = 15  Mean/median age = 40 | **Study recruitment: Mod risk bias**  Dependent on accuracy on recording on trauma registry and accuracy of case notes  Low-risk group - GCS15 and benign CT  **Attrition: Low risk**  Loss of 37 patients to follow-up  **Prognostic factor measurement: Low risk**  As recorded in case notes so dependent on accuracy  **Outcome measures: Low risk**  Prospective follow-up by trained staff using validated tool. Not clear what would happen to patients who died or deteriorated and attended a different hospital.  **Confounding factors:**  Patients who are perceived as higher risk will be put on ICU, likely to be differences in co-morbidities  **Statistical techniques: Low risk**  Well presented - not really relevant to meta-analysis  Only GCS15 patients with benign-looking CT scans |
| Schaller et al.  2015  Switzerland | Level 1 trauma center  Bern  Switzerland  Jan 2006-Dec 2007  Inclusion criteria:   - Admission GCS 13-15 - Observed for 24 h - Localized intra-cranial bleeds up to 5 mm - this is from the CCHR article   Exclusion criteria:   - Bleeds >5-mm maximum diameter - Multiple bleeds - History of bleeding tendency - Anti-coagulant or anti-platelet medication - Intoxication - Other injuries - Live alone - Live >1 h from hospital | Retrospective cohort study/case series  Aim:  To assess if a specific group of patients with small bleeds can be discharged from hospital without 24 h of observation | Deterioration in neurological status or need for neurosurgery | Prognostic factors are the inclusion/exclusion criteria  No comparison in risk of deterioration in 2 groups | 110 patients met inclusion and exclusion criteria.  None deteriorated within the period of hospital observation, required neurosurgery or re-attended.  Mean/median GCS = 14.6  Mean/median age = 40  Percent anti-coagulated = 0 | **Study recruitment: Low risk bias**  Retrospective cohort review - reliant on accuracy of written notes  **Attrition: Mod risk**  Patients may have moved out of catchment area of hospital without the researchers being aware. Loss to follow-up if re-presented different hospital.  **Prognostic factor measurement: Mod risk**  Reliability of case notes - may be incomplete  Interpretation size of the bleed was taken from written radiology report: ? reliability.  **Outcome measures: Moderate risk**  Study dependent on patients re-presenting at the same hospital following discharge if had delayed deterioration. Not clear how patients who died in the community would have been identified.  **Confounding factors: Low risk**  No obvious confounding factors  Cohort selection criteria including not living alone may select out high-risk older patients.  Statistical techniques: N/A  **General comments**  Mean age 39.9 years and 25% caused by sporting injuries. ?Age as the confounding low-risk prognostic factor. Not generalizable to older populations.  Small numbers |
| Levy et al.  2011  Colorado  USA | Level 1 trauma center  Denver  USA  Jan 1998-Dec 2008  Inclusion criteria:   - Admission ED GCS 13-15 - On trauma registry - Blunt head trauma - ICD 850-850.99 - consistent with concussion (i.e., no detected injury by CT) - Admitted to hospital - AIS score 2 before 2008 or 1/2 in 2008 - ICD-9 code for SAH   Exclusion criteria:   - Patient admitted directly to hospital - Multiple injuries; AIS score >1 head or other regions - Age <18 years - Not admitted | Retrospective cohort study  Aim:  To assess whether patients admitted with CT–VE mTBI have different outcomes to patients with mTBI and traumatic SAH  Uni-variate and multi-variate regression used to examine covariates and relationship to outcomes. | ED disposition  ICU admission  Neurosurgery  In-hospital mortality  Progression of SAH on CT | Age (18-39), (40-69), (70+)  Transfer status  Cause of injury  GCS  Blood alcohol level  Presence of skull fracture  CT report - divided into small/medium/large based on language included in report | 1144 patients admitted with mTBI but negative CT scan  117 with mTBI and traumatic SAH  1/117 - progression on repeat CT scan  0/117 required neurosurgical intervention  1/117 died (progression on CT)  4/1144 died  All patients died >70  **Logistic regression model tSAH versus concussion**  ICU admit adjusted OR 8.87 (5.62-14.02), *p* < 0.0001  ICU LOS >1D, OR 0.29 (0.11-0.74), *p* = 0.01  Hosp LOS >1D, OR 1.07 (0.67-1.69), *p* = 0.79  Mortality OR 2.46 (0.27-22.17), *p* = 0.42  Discharge to rehab  Age 18-39, OR 5.48 (0.25-121.70), *p* = 0.28  Age 40-69, 7.96 (1.91-33.11), *p* = 0.004  Age >70, 1.33 (0.50-3.53), *p* = 0.56 | **Study recruitment: Low risk bias**  Patients recruited from trauma registry depends on how good this is.  Only admitted patients - higher-acuity patients then discharged  Likely patients admitted for other reasons if CT negative TBI (although excludes other injuries).  **Attrition: Low risk**  All inpatient outcomes  **Prognostic factor measurement: Mod risk**  CT findings abstracted from CT reports - severity assigned by language not actually used in regression model  **Outcome measures: Moderate risk**  Only inpatient outcomes - possibility of discharge and deterioration  **Confounding factors: High risk**  Patients admitted with CT negative TBI likely to be frail or have other reasons for admission - this will affect outcome measures compared with SAH patients admitted due to +VE CT.  **Statistical techniques: Low risk**  Well presented  Can use for pooling for outcomes SAH - supports low-risk sub-population |
| Levy et al.  2014  USA | Level III rural non-neurosurgical unit in Rocky Mountains, USA  April 2007-Dec 2012  April 2007 patients with small bleeds selectively not transferred to neurosurgical unit.  Inclusion criteria:   - Admission GCS 13-15 - CT-positive intra-cranial injury - Not transferred to neurosurgical unit in accordance with non-transfer policy - CT findings of small SAH - Punctate or minimal contusion - Punctate or minimal intra-cranial bleed - Small SDH, no mass effect   Exclusion criteria:   - Any coagulopathy - Basilar skull fracture or evidence of CSF leak - Extra-dural bleed - Any significant contusion or SDH/intra-cerebral hemorrhage   Review and discussion of CT and patient with neurosurgeon if unsure if should be transferred | Retrospective cohort study  Aim:  Investigate outcomes after a novel non-transfer policy introduced in a small rural trauma unit without neurosurgical cover for mTBI patients with small ICH | Length of stay  Mortality  Neurological deterioration  Neurosurgery  Re-admission in 90 days of discharge  Inter-hospital transfer  Need for repeat CT | No comparison to patients who were transferred | 76/273 patients not transferred  >50% injuries due to skiing/snow boarding  71% patients <55 years of age  No patient deteriorated, died, or required neurosurgery or required delayed transfer while admitted to hospital.  2 patients re-admitted within 90 days - 1 patient 6 weeks following admission developed an acute chronic subdural - drained. 1 patient re-admitted with unrelated complaint.  Mean/median GCS = 14.7  Mean/median age = 36  Percent anti-coagulated = 0 | **Study recruitment: Low risk bias**  Retrospective cohort review - reliant on accuracy of written notes  CT inclusion criteria are subject and patients may have been transferred despite meeting non-transfer policy if clinicians were concerned.  **Attrition: Low risk**  Prognostic factor measurement: Mod risk  Reliability of case notes - may be incomplete  The definitions of bleed size are subjective.  **Prognostic factors**  N/A  **Outcome measures: Moderate risk**  Study dependent on patients re-presenting at the same hospital following discharge if they had delayed deterioration.  **Confounding factors: Low risk**  Age affect outcome and size of bleed  **Statistical techniques: N/A**  **General points**  Small numbers  No comparator group - need to compare with transferred patients outcomes  Patient not generalizable - v. young and atypical mechanism of injury (mostly winter sports related).  Likely that any patient that clinicians felt to be at risk would have been transferred even if patient did not meet transfer criteria - no way to check this. |
| Joseph et al.  2013  USA  The acute care surgery model: managing traumatic brain injury without an inpatient neurosurgical consultation | Level 1 trauma center  2009-2011 (likely subset of patients presented below)  Inclusion criteria:   - GCS13-15 - Trauma - Positive findings CT - skull fracture and/or ICH   Exclusion criteria:   - Pre-hospital anti-platelets or anti-coagulants | Retrospective cohort study  Propensity matching 1:2 ratio patients managed solely by trauma surgeons vs. patients who had neurosurgical consultation.  Hypothesis:  Trauma surgeons can manage mTBI patients with CT-detected intra-cranial hemorrhage without neurosurgical involvement. | Hospital admissions  ICU admissions  Neurosurgical interventions  ED visits after discharge  Mortality  Progression on CT imaging | Age  Sex  Initial GCS  ISS  Head-abbreviated injury score  Neurological examination  CT scan findings -type of skull fracture/type of ICH/size of bleed - reviewed by study investigator | 404 GCS13-15 patients with CT-detected injuries in study period  270/404 used for this study  90/270 had neurosurgical consultations (NC)  180 no neurosurgical consultation (no-NC)  Whether neurosurgical consultation requested as discretion of non-specialist surgeon. Propensity matching in this study between 2 groups.  0/270 neurosurgical interventions, hospital mortality, or readmissions either group  78/90 no-NC and 158/180 NC admitted hospital (*p* = 0.8)  18/90 no-NC and 80/180 NC admitted ICU (*p* = 0.001)  Routine repeat CT 18/90 no-NC 155/180 NC (*p* < 0.001)  No progression on any repeat CT  8% no-NC and 4% NC group re-attended ED. No readmissions.  Mean/median GCS = 15  Mean/median age = 30  Percent anti-coagulated = 0 | **Study recruitment: High risk bias**  Subset of patients that meet inclusion criteria selected to facilitate propensity matching. Possible selection out of higher-acuity patients as these will have all been referred to a neurosurgeon.  **Attrition: low risk**  In patient outcomes and documented ED re-attendances - low risk of patients being lost to follow up  **Prognostic factor measurement: Low risk**  All routinely collected clinical data apart from CT imaging, which was re-reviewed.  **Outcome measures: Mod risk**  Study dependent on patients re-presenting at the same hospital following discharge if they had delayed deterioration.  **Confounding factors: Mod risk**  Does not exclude patients with additional injuries  **Statistical techniques: High risk**  Does not outline how matched groups using propensity scoring  **General points**  Small numbers  Likely reporting data reported elsewhere |
| AbdelFattah et al.  2012  USA | Level 1 trauma center  Dallas  Texas  USA  Prospective recruitment 2010-2011  Inclusion criteria:   - Adult with ICH (note doesn’t explicitly state secondary to trauma - but implied)   Excluded:   - Age <16 years - GCS <13 - Undergone planned or immediate neurosurgery - Transferred patients | Prospective cohort study  Hypothesis:  Repeat CT imaging in GCS13-15 with ICH, without neurological progression, does not impact the need for neurosurgical intervention.  Patients divided into these 2 groups: patients with planned repeat CT imaging and those with CT imaging if deteriorated. Allocation by neurosurgeon - no deviation from normal practice. | Outcome measures during hospital admission:  Neurological progression  Medical intervention  Neurosurgical intervention  Repeat CT imaging - worse CT defined as worse by a blinded radiologist/neurosurgeon giving qualitative measure of bleed | Comparison between groups:  Age  Sex  Coagulation status  Anti-platelets  ISS  GCS | 145 patients met inclusion/exclusion criteria.  92/145 for routine repeat CT  53/145 for CT if deteriorated  Selective group more likely aspirin use, *p* = 0.02  Routine repeat CT worse head AIS score (*p* < 0.001)  Otherwise groups comparable  5/53 deteriorated and had a repeat CT + 1/53 had repeat scan as started on warfarin  1/145 patients died (due to other injuries)  27/145 radiological deterioration  9/145 patients intubated - states for other injuries  Mean/median GCS = 14.5  Mean/median age = 41  Percent anti-coagulated = 6 | **Study recruitment: Low risk**  Prospective recruitment - states recruited all eligible patients. Doesn’t explain how recruitment occurred.  **Attrition: Low risk**  Follow-up only for period in hospital  **Prognostic factor measurement: Low risk**  Blinded appraisal of CT scans by researcher  **Outcome measures: Mod risk**  No follow-up following discharge - missed delayed outcomes, could have looked for re-attendance.  Doesn’t report neurosurgical outcome measures.  **Confounding factors: High risk**  Not isolated head injury - other injuries have clearly affected outcome measures  **Statistical techniques: Low risk**  None  Small study with confounders regarding outcomes |
| Nayak et al.  2013  USA | University Hospital  Newark  New Jersey  USA  Level 1 trauma center  2003-2008  Inclusion criteria:   - Age ≥18 years - Blunt trauma - Intra-cranial bleed - Admitted to hospital - GCS13-15 on arrival to ED - GCS 15 24 h after attendance to ED   Excluded:   - History brain disease, e.g., dementia - Previous brain injury, e.g., CVA - Liver cirrhosis, renal disease, coronary artery disease, bleeding or clotting disorder - Unable to assess GCS due to drugs, e.g., sedation/intubation - Neurological deterioration leading to repeat CT - Age <15 years - Incomplete notes | Retrospective chart review  Aim:  To compare neurological outcomes of MHI patients with an intra-cranial bleed with a normal neurological examination managed with and without a repeat CT head scan | Neurosurgical intervention after 24 h - craniotomy, ventriculostomy, ICP bolt/measurement  Death in hospital  Discharge disposition  LOS hospital  GOS at follow-up clinic/re-attendance if applicable | Age  Sex  Mechanism of Injury  GCS on arrival  ISS  HAIS  GCS and neurological examination every 2 h - routine care on a flow sheet | 321/864 patients GCS13-15 with ICB met inclusion criteria  20% excluded because incomplete medical notes/transfers  0/321 neurosurgical intervention - all within 24 h of admission  No deaths  19/142 worse CT on repeat CT after 24 h of admission  179/321 single CT  142/321 routine repeat CT  76/321 returned to follow-up clinic - uneventful  14/321 returned to ED due to symptoms  Mean/median GCS = 14.9  Mean/median age = 41 | **Study recruitment: Low risk**  Retrospective case note review - depends on information being recorded correctly  **Attrition: Mod risk**  20% excluded because of incomplete notes  **Prognostic factor measurement: Mod risk**  Neuroradiology reports taken at face value - no verification  **Outcome measures: Mod risk**  No uniform follow-up of patients post-discharge. Some patients had follow-up clinic; others didn’t. Patients may have presented after discharge to other sites.  **Confounding factors: Low risk**  None obvious  **Statistical techniques: Low risk**  None completed  The inclusion/exclusion criteria have selected out all patients who are not GCS15 at 24 h. Different population than all GCS13-15 patients with TBI on CT - probably unable to pool these data.  Does show patients who are GCS 15 at 24 h low risk. |
| Anandalwar et al.  2016  New Jersey  USA | University Hospital  Newark  New Jersey  USA  Level 1 trauma center  2009-20012  Inclusion criteria:   - Age ≥18 years - Blunt trauma - Intra-cranial bleed/skull fracture - Admitted to hospital - GCS13-15 on arrival to ED - GCS15 24 h after attendance to ED - Did not receive a repeat CT head scan   Excluded:   - History of neurological or psychiatric disorder - Immediate neurosurgery - Previous TBI or neurosurgery - Spinal injury - Coagulopathy - Pregnancy - Transfers - Incomplete notes   Patients who did undergo a repeat CT scan despite meeting the rest of inclusion/exclusion criteria formed a comparison group | Retrospective cohort study  Aim:  Assess the outcomes following the implementation of a policy of observation only (no repeat CT imaging) for GCS 15 patients | Repeat CT after 24 h of admission due to clinical concern or deterioration  Progression on any repeat CT completed  Neurosurgical interventions  Intubation, ICU admissions, administration of mannitol  ED revisits within 1 year for TBI-related symptoms. | Age  Sex  Mechanism of Injury  ISS  AIS | 533 patients TBI and ICH  142 met the inclusion/exclusion criteria  47 underwent a routine repeat CT within 24 h (violation of policy) - 0/47 neurosurgical, 1/47 had incidental finding on CT  95 no repeat routine CT within 24 h  8/95 (non-violation group) had repeat CT >24 h after admission - due to concern  3/8 progression on CT  1 neurosurgical intervention  2/8 admitted to ICU due to deterioration - 1 intubated  3/95 patients returned within 1 year to the ED due to TBI symptoms - all underwent repeat CT. No admissions.  Mean/median GCS = 14.8  Mean/median age = 38  Percent anti-coagulated = 0 | **Study recruitment: High risk**  Patients at GCS15 at 24 h - low-risk group selected out - difficult to extrapolate to all GCS13-15 patients  Does not compare outcomes in patients who adhered to and violated non-routine repeat CT head imaging. Potentially, clinicians ordered routine repeat CT imaging on riskier patients.  **Attrition: Low risk**  Potential for patients to have re-attended at other EDs and be missed  **Prognostic factor measurement: Low risk**  No risk model developed  Factors abstracted from case notes  **Outcome measures: Low risk**  Re-attendance at other EDs makes re-attendance a potentially biased outcome measure  **Confounding Factors: Mod risk**  Cohort includes patients with multiple injuries  **Statistical techniques: Low risk**  None presented  Is a lower risk population due to selection for repeat CT imaging and return to GCS15 at 24 h - possibly unable to include in any meta-analysis |
| Ditty et al.  2015  Alabama  USA | University of Alabama  Level 1 trauma center  2003-20013  Inclusion criteria:   - 500 consecutive patients present on trauma registry - GCS13-15 - ICD-9 diagnosis SAH and/or intra-parenchymal contusion - confirmed with radiology report and neurosurgical consult note - if disagreement scan re-reviewed; if not clear patient excluded   Excluded:   - Diagnosis extra or subdural hematoma - Penetrating injuries - Fatal extra-cranial injuries - CSF leak - Aneurysmal SAH - Delayed presentation | Retrospective cohort study  Aim:  Assess the clinical implications of SAH or intra-parenchymal hemorrhage in mTBI | Neurological decline - altered mental state or focal neurological deficit  Inpatient seizure  Delayed neurosurgical evacuation as inpatient  Inpatient mortality | Admission GCS  Anti-coagulation  Anti-platelets  Transfer distances  Sex  Age  Hemorrhage type | 500 patients met inclusion criteria  411/500 isolated SAH  63/500 isolated ICH  26/500 both  463 GCS15  30 GCS14  8 GCS13  469/500 patients pre-hospital medication available (71/469 taking either anti-coagulants or anti-platelets)  156/500 transfers  No patients had seizures.  No patients had neurological decline.  No patients underwent delayed neurosurgical intervention.  No inpatient mortality | **Study recruitment: Mod risk**  High proportion of transferred patients may represent higher or lower acuity patients than general population.  Higher as being transferred to specialist center, lower as survived /fit to transfer  No details about inclusion or completeness of trauma registry  **Attrition: Low risk**  Only inpatient measures  **Prognostic factor measurement: Mod risk**  Incomplete information regarding medications  May be other inaccurate recording of factors  **Outcome measures: Mod risk**  Only inpatient-related outcome measures. Patients may have been discharged and deteriorated and presented to other hospitals.  **Confounding factors: Mod risk**  Cohort includes patients with multiple injuries - only excluded if died from other injuries.  **Statistical techniques: N\|A**  None presented  Narrative synthesis - further evidence SAH low risk |
| Pruitt et al.  2016  Chicago  USA | Level 1 trauma center  Chicago  2009-2013  Inclusion criteria:   - Initial GCS13-15 - Age ≥16 years - Traumatic intra-cranial bleed or skull fracture - Identified on electronic ED system using ICD-9 classification system - Admitted to ED observation unit   All patients received a neurosurgical consultation. | Retrospective cohort study  Aim:  Assess if mTBI patients with intra-cranial hemorrhage can be managed to an ED observation unit. | Clinical deterioration (defined as decrease in mental status, worsening neurological exam or death)  Neurosurgery during admission  Progression on CT | Age  Gender  Method of arrival  Whether transfer  Comorbidities  Anti-coagulant use  Mechanism of injury  Initial GCS  Neurological examination  Alcohol intoxication  Initial platelet count INR  Initial CT results  Follow-up CT results  Neurosurgical recommendations  Cranial CT data were collected from attending radiologist reports - type and size of detected injury | 1185 GCS13-15 with CT detected injuries  814 admitted directly to hospital - poly-trauma, social reasons, or as neurosurgeons felt high risk  371 left under care of ED. Of these, 239/371 transferred to ED observation unit. 132/371 discharged directly from the ED after a period of observation.  **Admitted patients**  Clinical deterioration  15/814 - worsening CT  27/814  Neurosurgery  33/814  Composite outcome 75/814  **ED observation unit**  Clinical deterioration   0/239  Worsening CT   11/239  Neurosurgery   3/239  Composite outcome 14/239  Medical admission 4/239  Trauma/neurosurgery admit 8/239  **Follow-up 190/239**  Delayed neurosurgery   0/239  Post-traumatic seizure 3/239  Concussive symptoms 16/239  **Discharged ED**  **Follow-up 111/132**  Delayed neurosurgery   1/132  Post-traumatic seizure 2/132  Concussive symptoms 8/132  Figures from table - author has confirmed this is correct:  *155 isolated SAH - 0 no clinical or radiological deterioration or cases of neurosurgery*  *161 SDH - 6 CT deterioration*  *3 planned neurosurgical outcomes*  *0 deteriorated clinically*  *1 neurosurgery >3 weeks later following outpatient assessment*  *30 contusion, 5 worsening CT scans. Nil clinical deterioration or emergency neurosurgery*  *5 extra-dural - nil deterioration or neurosurgery*  Of sample 1053 mean/median age = 59, 11% anti-coagulated  Of sample 1185 mean median age = 59, 10% anti-coagulated | **Study recruitment: High risk**  Neurosurgeons have admitted higher=risk patients - we can combine outcomes from both admitted and ED observed patients to give an unbiased estimate.  **Attrition: Med risk**  Only a proportion of patients are followed up - does not describe the mechanism for this or how consistent follow-up is, e.g., did they all get repeat CT scans  Prognostic factor measurement: medium risk  Dependent on CT scan reports and written documentation  **Outcome measures: Mod risk**  Clinical deterioration not well defined and very broad  **Confounding factors: Low risk**  Included patients with polytauma and significant comorbidities  **Statistical techniques: High risk**  None presented, but data presented in table and text do not match up.  Article shows patients admitted to hospital by neurosurgeons have worse outcomes/more likely to require neurosurgery.  Does show that in America some of this patient population discharged directly from ED. Consistent with the model used locally in Hull. |
| Deepika et al.  2013  Bangalore  India | Patients admitted to tertiary neurosurgical center during 3 months, Jan-March 2010  Patients identified on a TBI registry  Inclusion criteria:   - GCS13-15 head injury - Underwent CT scan - Either negative CT or isolated traumatic subarachnoid - Matched comparison between patients -VE CT and SAH   Excluded:   - Does not state adults only but age range 15-67 | Retrospective cohort study  Aim:  To assess whether GCS13-15 patients with traumatic subarachnoid hemorrhage have the same outcomes as mTBI patients with -VE CT scans | Prospective 1-year telephone assessment of :  GOSE  Rivermead post-concussion questionnaire  Rivermead head injury follow-up questionnaire | Age  Sex  Mechanism of injury  RTC  Fall  LOC  Seizure  Location of SAH  Whether multiple bleeds  Thickness > or < than 5 mm | 34/1628 mTBI patients isolated traumatic subarachnoid hemorrhage  18/34 patients available for follow-up at 1 year  Good GOS-E  Rivermead scores comparable to 16 normal CT controls | **Study recruitment: Low risk**  Cohort identified in TBI registry, which is part of normal practice.  Is retrospective so limited by accuracy of medical notes  **Attrition: High risk**  Small sample - with large proportion lost to follow-up  **Prognostic factor measurement: Medium** **risk**  Dependent on CT scan reports and written documentation  **Outcome measures: High risk**  1 year too long  **Confounding factors: Medium risk**  No control for other injuries or comorbidities  **Statistical techniques: N/A**  Too poor quality to include |
| Kreitzer et al.  2014  Cincinnati  USA | Level trauma center  2001-2010  Identified from cohort of patients who had undergone 2 CT within the ED within 24 h  Inclusion criteria:   - GCS14-15 and blunt head injury - Presented within 24 h of injury - Intra-cranial bleed first CT defined extra-dural, subdural, SAH, intra-cerebral, and cerebral contusion - 2nd CT within 24 h   Excluded:   - Incomplete notes - Pregnant - Intubated prior to ED evaluation - Abnormal observations - Penetrating injury - CT scans interpreted at different hospital - Coagulopathy either inherited or acquired - INR >1.4 (even if taking warfarin) - Platelets <50 - Any non-head injury mandating admission - Age <18 years | Retrospective cohort study  Standard practice repeat CT at least 6 h after 1st CT if mTBI with ICH. If CT and patient stable, discharge from ED.  Aim:  Assess outcomes for patients with mTBI and ICH | Death within 30 days  Neurosurgical intervention within 2 weeks  Return to the Ed within 7 days of discharge | CT head findings  Age  Race  Sex  Medical background | 323/1011 patients who under-went 2 CT head scans within 24 h in ED met the inclusion criteria.  After second CT:  92/323 admitted  25/323 observed in ED and subsequently discharged  206/323 discharged  4 patients died (3 admitted, 1 discharged). States death in discharged patient unlikely to be related to head injury; had further fall. Also 1 other patient died of septic shock.  3 neurosurgical interventions (all admitted)  28/206 discharged patients returned to ED within 1 week. None re-admitted and some planned - removal of sutures.  Mean/median age = 42  Percent anti-coagulated = 0 | **Study recruitment: Mod risk**  Identified through repeat CT imaging in ED - relies on all of cohort having repeat scans, and patients deteriorate and not undergoing second scan being missed  **Attrition: Low risk**  Followed up through social security system for deaths, and the rest are inpatient outcome. Possibility of patients re-attending at other EDs.  **Prognostic factor measurement: Medium** risk  States that some CT are reported by radiology trainees overnight and then corrected by attending radiologists the next day - unable to quantify how much inaccuracy there is.  Does state 32% of repeat scans normal.  **Outcome measures: Low risk**  Reasonable outcome measures  **Confounding factors: Low risk**  Controls for comorbidities and other injuries  **Statistical techniques: N/A** |
| Ding et al.  2012  Neurosurgical center  China | Neurosurgical center  China  2009-2010  Inclusion criteria:   - All patients with TBI with evidence of intra-cranial hemorrhage - some data for GCS13-15   Excluded:   - Immediate neurosurgery - Died within 3 days - Severe multiple injuries - Failed to undergo a repeat CT head | Appears to be a random control trial comparing outcomes in patients with traumatic intra-cranial hemorrhage assigned either to a routine repeat CT or CT only if deteriorates | GCS at discharge  Surgical and medical interventions secondary to CT | CT scan results  Initial GCS  Mechanism of Injury  Coagulation INR and platelets | 32/89 patients in routine CT group GCS13-15  2/32 worse CT scans  No patients had neurosurgery or altered medical management.  Mean/median age = 48 | **Study recruitment: High risk**  Allocation to intervention and non-intervention arm not clearly explained - states via random number generator  **Attrition: Low risk**  Low risk - inpatient outcomes  **Prognostic factor measurement: Medium** **risk**  No re-reporting of CTS  **Outcome measures: Medium Risk**  No outcome measures after discharge  **Confounding factors: Low Risk**  Controls for other injuries  **Statistical techniques: N/A** |
| Huynh et al.  2006  USA | Level 1 trauma center  2004-2005  Identified case note review  Inclusion criteria:   - mTBI - Blunt trauma to head - GCS15 - Abnormal CT head   Excluded:   - Normal initial CT head - Length of admission less than 48 h - Age < 18 years | Retrospective cohort study  Aim:  To assess whether neurosurgical review is necessary in GCS15 patients with intra-cranial injuries | Changes on follow-up CT - all patients had routine repeat CT  Neurosurgical intervention | Demographics  Mechanism of Injury  ISS  LOC  Amnesia  Associated injuries | 56 patients met inclusion criteria  4/56 patients worse repeat CT  Of these 4:  2/56 patients had fall in GCS to 14 from 15  1/56 given mannitol due to worse CT  1/56 loaded with phenytoin for seizures  No consistent measure of deterioration  0/56 neurosurgical interventions  0/56 deaths  Mean/median GCS = 15  Mean/median age = 41 | **Study recruitment: Medium risk**  Weaknesses of a retrospective case note review  Higher-risk group as admitted for at least 48 h  **Attrition: Low risk**  Low-risk - inpatient outcomes  **Prognostic factor measurement: Medium** **risk**  No re-reporting of CTS  **Outcome measures: Medium risk**  No outcome measures after discharge  **Confounding factors: Low risk**  No controls for other injuries  **Statistical techniques: N/A** |
| Almenawer et al.  2013  Ontario  Canada | Neurosurgical center  Ontario  Canada  2006-2011  Identified from trauma database  Inclusion criteria:   - GCS13-15 - Blunt traumatic head injury - Age >17 years - Intra-cranial injury CT head - Repeat CT scan   Excluded:   - No repeat CT scan - Previous craniotomy - Cranial pathology - Coagulopathy - Immediate neurosurgery   Patients divided into those who underwent intervention due to clinical deterioration or due to repeat CT findings | Retrospective cohort study + meta-analysis to assess whether repeat CT imaging necessary in mTBI with intra-cranial hemorrhage | Intervention including:  Mannitol or hypertonic saline  Surgical intervention including ICP bolt or craniotomy  Neurological changes: decrease GCS, cranial nerve change, vomiting, and headache | Demographics  GCS  ISS | 1121 patients with mTBI and ICH  445 met inclusion criteria  91/445 worse CT  21/445 patients neurosurgical outcomes (all preceded by clinical deterioration prior to repeat CT)  4/445 patients medical intervention  2/4 medical outcomes = treated with mannitol due solely to worse CT; other 2 treated due to clinical deterioration  Mean/median GCS = 14.5  Mean/median age = 45  Percent anti-coagulated = 0 | **Study recruitment: High risk**  Dependent on accuracy of trauma database  Large proportion of mTBI patients with ICH did not meet inclusion criteria - selection out of higher-risk patients who did not undergo repeat imaging  **Attrition: Low risk**  Low risk - inpatient outcomes  **Prognostic factor measurement: Medium** **risk**  No re-reporting of CTS  **Outcome measures: Medium risk**  No outcome measures after discharge  **Confounding factors: Low risk**  No control for polytrauma  **Statistical techniques: N/A** |
| Sifri et al.  2004  USA | Level trauma center  New Jersey  USA  1999-2001  Inclusion criteria:   - GCS 14-15 - Blunt traumatic head injury - Age >15 years - Intra-cranial injury, CT head - Repeat CT   Excluded:   - History of brain injury - Coagulopathy including known bleeding disorder or taking warfarin - Immediate neurosurgical intervention including transfer to ICU | Retrospective cohort study  Aim:  To assess the value of routine repeat CT imaging in mTBI patients with intra-cranial hemorrhage | Worse CT  Inpatient neurological deterioration - abnormal neurology - confusion, disorientation or drowsiness  Inpatient neurosurgical interventions | CT results as abstracted from radiologist and neurosurgeons reports  Best ED GCS  Demographics | 243 patients with mTBI and ICH  18/243 excluded as no repeat CT - neurosurgeon ruled insignificant lesion  202/243 included as met the rest of inclusion criteria  At 24 h:  151/202 persistently normal or improving neurology  51/202 persistently abnormal or worsening neurological examination  50/202 worse CT  5/202 required neurosurgery - all had persistent or worsening neurology  1/202 died; in the persistently abnormal/worsening neurology group  No clear measure of deterioration  Mean/median GCS = 14.7  Mean/median age = 44  Percent anti-coagulated = 0 | **Study recruitment: Medium risk**  Selection out of patients not undergoing repeat CT head imaging  **Attrition: Low Risk**  Low risk - inpatient outcomes  **Prognostic factor measurement: Medium** **risk**  The definition of abnormal neurology is loose and not clear when it developed - not an admission criteria factor  **Outcome measures: Medium risk**  No outcome measures after discharge  **Confounding factors: Low risk**  No control for polytrauma and comorbidites  **Statistical techniques: N/A** |
| Phelan et al. 2014  Dallas  USA | Level 1 trauma center Dallas  Texas  USA  2010-2012  Patients identified on TBI database  Inclusion criteria:   - Intra-cranial hemorrhage - TBI - Patients divided into SAH and non SAH bleed - All GCS but data for GCS13-15 patients presented   Excluded:   - Ages < 18 years - Pregnant - Prisoners | Retrospective cohort study  Assess whether outcomes for mTBI with isolated traumatic subarachnoid differ for other kinds of intra-cranial bleeds | Worse repeat CT imaging if any  Death  Craniotomy | CT findings as re-read by a study team member  Age  ISS  HAS  Emergency department GCS | 77 patients GCS13-15 and traumatic SAH  27/77 scheduled repeat CT  3/27 worse CT  50/77 - no routine repeat CT  4/50 - unscheduled repeat CT  1/50 - clinical deterioration and worse CT  4/77 worse CT  0 neurosurgical intervention | **Study recruitment: Low risk**  Dependent on accuracy of trauma registry  **Attrition: Low risk**  Low risk- inpatient outcomes  **Prognostic factor measurement: Low** **risk**  Does not really assess prognostic value of factors measured  **Outcome measures: Medium risk**  No outcome measures after discharge  **Confounding factors: Low risk**  No control for polytrauma and comorbidites  **Statistical techniques: N/A** |
| Homnick et al.  2012  New Jersey  USA | New Jersey Medical School  Level 1 trauma center  2002-2005  Inclusion criteria:   - Age >17 years - GCS >12 - TBI with positive initial CT-intra-cerebral bleed, contusion, subdural, extra-dural or SAH   Excluded:   - Penetrating trauma - Injury >24 h previously - Previous neurosurgery - Non-traumatic mass on CT - Immediate neurosurgery | Retrospective cohort study  Establish how long intra-cranial bleeds in mTBI continue to expand | Neurosurgical intervention  Progression on CT - repeat CTs at discretion of neurosurgeon | Age  Sex  Pre-injury anti-coagulation  Mechanism  ISS  Initial GCS | 341 patients in study (85 mTBI patients with bleeds excluded as no follow-up scan)  72/341 intubated in ED  105/341 progression on CT  13/341 death - 9 due to TBI, 4 other causes  12/341 neurosurgical intervention  Mean/median GCS = 14.6  Mean/median age = 47  Percent anti-coagulated = 2 | **Study recruitment: Medium risk**  Selection out of lower-risk patients who did not have repeat CT imaging  **Attrition: Low risk**  Low risk- inpatient outcomes  **Prognostic factor measurement: Low** **risk**  Does not really assess prognostic value of factors measured  **Outcome measures: Medium risk**  No outcome measures after discharge  **Confounding factors: Medium risk**  No control for polytrauma and comorbidites  **Statistical techniques: N/A** |
| Nasir et al.  2011  Karachi  Pakistan | Specialist center  Karachi  Non-probability consecutive sampling  Inclusion criteria:   - GCS14-15 - All ages - 15% sample children; mean age 36, 2 SD 18 - TBI with positive initial CT intra-cranial injury   Excluded:   - Clinical deterioration - Immediate neurosurgery - Isolated pneumocephalus   All patients had a repeat CT within 72 h | Retrospective cross-sectional study  Aim:  To assess the utility of repeat CT scanning in mTBI patients with intra-cranial injuries without clinical or neurological deterioration | Worse CT | Age  Gender  Initial GCS  Mechanism of injury  CT findings | 275 patients met inclusion criteria (note states 255 contusion hematoma)  17/275 worse CT  No patients required neurosurgery.  Mean/median GCS = 14.7  Mean/median age = 36  Percent anti-coagulated = 0 | **Study recruitment: Medium risk**  Does not adequately define deterioration or over what period  **Attrition: Low risk**  Low risk- inpatient outcomes  **Prognostic factor measurement: Low risk**  Does not really assess prognostic value of factors measured  **Outcome measures: Medium risk**  No outcome measures after discharge  **Confounding factors: Medium risk**  No control for polytrauma and comorbidites  **Statistical techniques: N/A**  **Overall**  Includes children and quite a different population than North America and Europe |
| Boris et al.  2013  Israel | Israel  Level 2 trauma center  Sates 2007-2011  Inclusion criteria:   - GCS14-15 - TBI with positive initial CT intra-cranial injury including subdural, extra-dural, subarachnoid, and intra-cerebral bleeds - Only data for adults presented   Excluded:   - Patients with incomplete data - Transferred to neurosurgery immediately - No repeat CT   All patients had a repeat CT within 12 h | Retrospective cohort study  Assess whether repeat CT imaging in GCS14-15 mTBI with intra-cranial injury justified | Increased size of bleed second CT  Clinical deterioration - decrease in GCS  New motor or sensory symptoms  Severe headache or vomiting | Age  Sex  Initial and follow-up GCS  CT findings | 68 patients  4 patients transferred to neurosurgery (2 routine)  8/68 patients worse CT  12/68 mild deterioration  28 patients intra-parenchymal bleed  1/28 worse CT  3/28 neurological deterioration  1/28 transferred to neurosurgery (not patient with worse CT)  7 patients extra-dural  1/7 worse CT  0/7 neurological change  1/7 transferred to neurosurgery  20 patients subdurals  3/20 worse CT  4/20 neurological deterioration  1/20 neurosurgery  13 patients SAH  3/13 increase in size bleed  5/13 neurological deterioration  1/13 transferred to neurosurgery  Mean/median GCS = 14.8  Mean/median age = 56 | **Study recruitment: Medium risk**  Identified on trauma database with patients with incomplete data excluded. Does not present number of these patients. Also excludes patients transferred immediately. Likely to be lower-risk sample than population of interest.  **Attrition: Low risk**  Low risk - inpatient outcomes  **Prognostic factor measurement: Low** **risk**  Does not really assess prognostic value of factors measured  **Outcome measures: Medium risk**  No outcome measures after discharge  **Confounding factors: Medium risk**  No control for polytrauma and comorbidites  **Statistical techniques: N/A** |
| Brown et al.  2007  Los Angeles  USA | Los Angeles  USA  Level 1 trauma center  2003-2004  Inclusion criteria:   - All patients with blunt head trauma and intra-cranial bleed on initial CT. Presents data for GCS13-15.   Excluded:   - Immediate neurosurgery - Died within 24 h - Does not state just adults but seems only for adults (mean age 44 ± 19) | Prospective cohort study  Aim:  To identify patients with head injuries who benefit from routine repeat CT imaging | Need for neurological intervention - either medical or surgical (medical = sedatives, mannitol, or hyperventilation and surgical = ICP monitor and craniotomy)  Mortality | Age  Gender  Mechanism of injury  ISS  Admission GCS  Results of CT - interpreted by attending radiologist | 354 patients, all GCS scores with intra-cranial bleed  37 direct to craniotomy  43 died within 24 h  274 = study population  142/274 = mTBI GCS13-15  15/142 had clinical deterioration  27/142 had worse CT scans (only 72/142 had repeat imaging)  5/142 had medical or neurosurgical intervention  3/142 died  Mean/median GCS = 14  Mean/median age = 43 | **Study recruitment: Mod risk**  Removal of patients who died within 24 h may lead to this sample being a lower-risk group than population of interest  **Attrition: Low risk**  Low risk - inpatient outcomes  **Prognostic factor measurement: Low** **risk**  Does not really assess prognostic value of factors measured  **Outcome measures: Medium risk**  No outcome measures after discharge  **Confounding factors: Medium risk**  No control for polytrauma and comorbidities-  **Statistical techniques: N/A** |
| Thomas et al.  2010  Tennessee  USA | Tennessee  USA  Level 1 trauma center  50 months from Jan 2001  Inclusion criteria:   - All patients with blunt head trauma and evidence of TBI on initial CT. Presents data for GCS13-15 - Age 18+ years   Excluded:   - Penetrating mechanism - Immediate neurosurgery - Interventions for unclear indications - Died before second CT   All patients had repeat CT at 6-8 h after admission | Retrospective cohort study  To assess whether scheduled repeat CT head imaging is indicated in TBI | Neurosurgical interventions - craniotomy or ICP monitor  Medical interventions - mannitol/hypertonic saline  Neurological change - reduced GCS, pupillary change, increased ICP, or loss of brainstem reflexes | Initial GCS  ISS  Race  Age  Gender  Mechanism of injury  History of vascular disease  Anti-coagulant use  Anti-platelet use  PT, aPPT, INR  CT findings | 457/836 in included sample population GCS13-15  14/457 = neurosurgical intervention (craniotomy or ICP bolt)  3/457 medical management  5/14 neurosurgical interventions - based on repeat CT  3/14 medical interventions - based on repeat CT  Mean/median age = 42 | **Study recruitment: Mod risk**  Dependent on case note review. Patient with “unclear” indications for interventions removed.  **Attrition: Low risk**  Only inpatient outcome measures  **Prognostic factor measurement: Mod risk**  Does not explain how CT scans reported  **Outcome measures: Mod risk**  No F/U after discharge  **Confounding factors: Medium risk**  No control for polytrauma  **Statistical techniques: N/A**  None done |
| Klein et al.  2010  Israel | 3 regional trauma center in Israel. None had access to neurosurgery onsite.  Identified ICD-9 codes on national trauma registry.  Inclusion criteria:   - GCS13-15 - ICD-9 code for intra-cranial bleed.   One hospital transferred all patients to neurosurgical center.  Other 2 hospitals transferred selected patients. | Retrospective cohort study  Aim:  Assess the outcome of low-risk patients with ICB managed in district hospitals without neurosurgical services | Mortality  Neurosurgical intervention  Neurological status at discharge | Age  AIS  ISS | 323 patients, all 3 hospitals, intra-cranial bleed and GCS13-15  27/323 required neuro-rehab  2/323 died  35/323 neurosurgery  77/323 not transferred-  0/77 died  0/77 neurosurgery  2/77 delayed transfer  Non-transfer on basis of:  Single bleed ≤ 5mm or contusion <1cm and no coagulopathy  Mean/median age = 39 | **Study recruitment: Low risk**  Dependent on completeness of trauma registry  **Attrition: Low risk**  Only inpatient outcome measures  **Prognostic factor measurement: Mod risk**  Does not explain how CT scans reported  **Outcome measures: Mod risk**  No F/U after discharge  **Confounding factors: Medium risk**  No control for polytrauma or comorbidities  **Statistical techniques: N/A**  None done |
| Sifri et al.  2011  USA | Level 1 trauma center  New Jersey  USA  2002-2006  Inclusion criteria:   - Initial GCS13-15 - Blunt traumatic head injury - Age 18+ years - Intra-cranial injury CT head - ICB or skull fracture - Repeat CT - Abnormal neurological examination at time of repeat CT   Excluded:   - Immediate or planned neurosurgical intervention - Normal neurology at time of repeat CT - normal neurology defined as GCS15, orientation to place, person, or time, normal neurological exam, no symptoms from head injury: headache, vomiting, dizziness, lethargy - Coagulopathy including known bleeding disorder or taking warfarin - Pregnancy - Spinal cord Injury - Prior brain surgery - Acquired or congenital cerebral pathology or existing neurological or psychiatric disorder | Retrospective cohort study  Aim:  To assess proportion of patients who have worse CT scans and neurosurgical interventions who have abnormal neurology when they have a repeat CT | Progression of lesion on CT  Surgical intervention - includes intubation  Medical intervention  GOS-E at discharge | Demographics  Acute deterioration in neurological exam  Persistently abnormal neurological exam  Unknown whether change as intubated | 107 patients met inclusion criteria  63/107 worse CT = 59%  7/107 neurosurgical group  21/107 deterioration  18/107 unable to assess neurology as intubated  6 died  Mean/median GCS = 14.4  Mean/median age = 48  Percent anti-coagulated = 0 | **Study recruitment: High risk**  High-risk subgroup that have abnormal neurology at time of repeat CT imaging  **Attrition: Low risk**  Only inpatient outcome measures  **Prognostic factor measurement: Mod risk**  Difficult to assess deterioration in a retrospective study  **Outcome measures: Mod risk**  No follow-up after discharge  **Confounding factors: Low risk**  Some control for comorbidities  **Statistical techniques: N/A**  None done |
| Beynon et al.  2015  Germany | Heidelberg University Hospital  Germany  2013-2014  Inclusion criteria:   - Initial GCS13-15 - Traumatic Intra-cranial bleed CT head | Retrospective cohort study  Aim:  Compare outcomes in patients on different types of anti-coagulants | Repeat CT imaging  Progression on CT  Neurosurgery  Death  Mean GCS at discharge | Patients divided into those on no anti-coagulants, aspirin, warfarin, and DOACs.  Gender  Trauma mechanism  Comorbidities,  CT findings  Repeated CT imaging  Age  GCS scores  Laboratory values | 70 patients met inclusion criteria  37 no anti-coagulation  27 anti-platelets  5 warfarin  6 DOACs (rivaroxaban)  1 patient dabigatran  25% neurosurgery (18 patients)  43/70 repeat CT imaging  2 deaths, both on rivaroxaban  Mean/median GCS = 14.5  Mean/median age = 67  Percent anti-coagulated = 16 | **Study recruitment: Low risk**  Although high rates of anti-coagulation  **Attrition: Low risk**  Only inpatient outcome measures  **Prognostic factor measurement: Low risk**  May be mis-classified in medical notes  **Outcome measures: Mod risk**  No follow-up after discharge  **Confounding factors: Low risk**  No control for comorbidities  **Statistical techniques: N/A**  None done |

|  | | | | | | |
| --- | --- | --- | --- | --- | --- | --- |
| *Studies only included in meta-analysis of prevalence of outcomes (continued;* n *= 26)* | | | | | | |
| *Reference* | *Population* | *Study design* | *Outcome measures* | *Prognostic factors assessed* | *Results* | *Quality appraisal* |
| Nishijima et al.  2013  Sacramento  USA  Variability of ICU Use in adult patients with minor traumatic intra-cranial haemorrhages | Multicenter; 8 sites Western USA; all Level 1  Trauma registries searched for ICD-9 codes intra-cranial hemorrhage 2005-2010  Inclusion criteria:   - Age >18 years - Traumatic ICH - Initial ED GCS15 - ISS < 16 | Retrospective cohort Study  Objective:  1) Assess the variability of ICU use in a cohort of patients with minor traumatic intra-cranial hemorrhages across multiple trauma centers  2) Estimate the proportion of minor traumatic intra-cranial hemorrhage patients admitted to ICU who do not receive an ICU intervention | Initial ICU admission from ED  Proportion of patients receiving critical care intervention defined as:  Neurosurgical intervention  Mechanical ventilation  Vasopressor/ionotropic use  Transfusion blood product  Invasive monitoring | Age  Initial GCS  Initial BP  LOS hospital  ICU stay  Procedures as coded in trauma registry  AIS | 11,240 patients coded as bleeds  771 excluded due to missing data  1412 remaining met inclusion criteria.  888/1412 admitted ICU, significant variation between sites  44/1412 (3.1%) had critical care intervention  6/1412 neurosurgical intervention  847/888 patients admitted ICU no critical care intervention  Mean/median GCS = 15  Mean/median age = 48 | **Study recruitment: Mod risk bias**  Dependent on accuracy of recording on trauma registry. Does have some quality assessment of data imputation  Note initial GCS15 - lower risk group  **Attrition: Low risk**  Follow-up only during hospital admission  **Prognostic factor measurement: Low risk**  Doesn’t really apply as testing disposition not outcomes  **Outcome measures: Low risk**  No measure of outcomes after discharge, but study primarily about disposition.  Does not report deaths.  **Confounding factors:**  States IIS increases ICU admission - will be related to other injuries  **Statistical techniques: Low risk**  N/A  **Overall**  Only GCS15 patients with low ISS |
| Nishijima et al. 2015  Sacramento  USA  Long-term Neurological Outcomes in Adults with Traumatic Intracranial Hemorrhage Admitted to ICU versus Floor | Level1 trauma center  2008-2013  Inclusion criteria:   - Age >18 years - Identified ICH ICD-9 code trauma registry - Initial ED GCS15 - Isolated head injury based on AIS score - Age <65 years - No evidence midline shift CT - Present on TBI database due to suspected TBI/evidence of ICH | Retrospective cohort Study  Aim:  Compare long-term neurological outcomes in low-risk patients with traumatic intra-cranial hemorrhage (tICH) admitted to the ICU vs. patients admitted to the floor. | Prospective long-term outcome measure at 6 months  Either GOS-E8 fully recovered or GOS-E1-7 not fully recovered | Age  Sex  Mechanism of injury  Initial ED GCS score  Initial (SBP)  Heart rate, respiratory rate, blood alcohol level  AIS score  ISS score  INR  Rotterdam CT score | 188 met inclusion criteria  151/188 complete data = cohort  106 admitted ICU (70%)  45 admitted ED (30%)  1/151 patients neurosurgical intervention as inpatient  1/151 patient died as inpatient  78 (52%) GOS-E8 at 6 months  Does present analysis for outcome at 6 months GOS-E but no inpatient measures of deterioration  Adjusted analysis, floor admission vs. ICU had an odds ratio of 0.77 (95% CI [0.36-1.64]) for a GOS-E score of 8 at 6 months.  Mean/median GCS = 15  Mean/median age = 40 | **Study recruitment: Mod risk bias**  Dependent on accuracy on recording on trauma registry and accuracy of case notes  Low-risk group - GCS15 and benign CT  **Attrition: Low risk**  Loss of 37 patients to follow-up  **Prognostic factor measurement: Low risk**  As recorded in case notes so dependent on accuracy  **Outcome measures: Low risk**  Prospective follow-up by trained staff using validated tool. Not clear what would happen to patients who died or deteriorated and attended a different hospital.  **Confounding factors:**  Patients who are perceived as higher risk will be put on ICU, likely to be differences in comorbidities.  **Statistical techniques: Low risk**  Well presented - not really relevant to meta-analysis  Only GCS15 patients with benign-looking CT scans |
| Schaller et al.  2015  Switzerland | Level 1 trauma center  Bern  Switzerland  Jan 2006-Dec 2007  Inclusion criteria:   - Admission GCS13-15 - Observed for 24 h - Localized intra-cranial bleeds up to 5 mm - this is from the CCHR article.   Exclusion criteria:   - Bleeds > 5 mm maximum diameter - Multiple bleeds - History of bleeding tendency - Anti-coagulant or anti-platelet medication - Intoxication - Other injuries - Live alone - Live >1 h from hospital | Retrospective cohort study/case series  Aim:  To assess if a specific group of patients with small bleeds can be discharged from hospital without 24 h of observation | Deterioration in neurological status or need for neurosurgery | Prognostic factors are the inclusion/exclusion criteria.  No comparison in risk of deterioration in 2 groups. | 110 patients met inclusion and exclusion criteria.  None deteriorated within the period of hospital observation, required neurosurgery, or re-attended.  Mean/median GCS = 14.6  Mean/median age = 40  Percent anti-coagulated = 0 | **Study recruitment: Low risk bias**  Retrospective cohort review - reliant on accuracy of written notes  **Attrition: Mod risk**  Patients may have moved out of catchment area of hospital without the researchers being aware. Loss to follow-up if re-presented at different hospital.  **Prognostic factor measurement: Mod risk**  Reliability of case notes - may be incomplete.  Interpretation size of the bleed was taken from written radiology report - ?reliability  **Outcome measures: Moderate risk**  Study dependent on patients re-presenting at the same hospital following discharge if had delayed deterioration. Not clear how patients who died in the community would have been identified.  **Confounding factors: Low risk**  No obvious confounding factors  Cohort selection criteria including not living alone may select out high-risk older patients.  **Statistical techniques:**  N/A  **General comments:**  Mean age 39.9 years and 25% caused by sporting injuries. ?Age as the confounding low-risk prognostic factor. Not generalizable to older populations  Small numbers |
| Levy et al.  2011  Colorado  USA | Level 1 trauma center Denver  USA  Jan 1998-Dec 2008  Inclusion criteria:   - Admission ED GCS13-15 - On trauma registry - Blunt head trauma - ICD 850-850.99 - consistent with concussion (i.e., no detected injury by CT) - Admitted to hospital - AIS score 2 before 2008 or 1/2 in 2008 - ICD-9 code for SAH   Exclusion criteria:   - Patient admitted directly to hospital - Multiple injuries AIS score >1 head or other regions - Age < 18 years - Not admitted | Retrospective cohort study  Aim:  To assess whether patients admitted with CT–VE mTBI have different outcomes to patients with mTBI and traumatic SAH  Uni-variate and multi-variate regression used to examine covariates and relationship to outcomes | ED disposition  ICU admission  Neurosurgery  In-hospital mortality  Progression of SAH on CT | Age (18-39),(40-69), (70+)  Transfer status  Cause of injury  GCS  Blood alcohol level  Presence of skull fracture  CT report - divided into small/medium/large based on language included in report | 1144 patients admitted with mTBI but negative CT scan  117 with mTBI and traumatic SAH  1/117 - progression on repeat CT scan  0/117 required neurosurgical intervention  1/117 died (progression on CT)  4/1144 died  All patients died >70  **Logistic regression model tSAH versus concussion**  ICU admit adjusted OR 8.87 (5.62-14.02), *p* < 0.0001  ICU LOS>1D OR0.29 (0.11-0.74), *p* = 0.01  Hosp LOS>1D OR1.07 (0.67-1.69), *p* = 0.79  Mortality OR2.46 (0.27-22.17), *p* = 0.42  Discharge to rehab  Age18-39 OR 5.48 (0.25-121.70), *p* = 0.28  Age 40-69 7.96 (1.91-33.11), *p* = 0.004  Age >70 1.33 (0.50-3.53), *p* = 0.56 | **Study recruitment: Low risk bias**  Patients recruited from trauma registry, depends on how good this is  Only admitted patients - higher-acuity patients then discharged  Likely patients admitted for other reasons if CT negative TBI (although excludes other injuries).  **Attrition: Low risk**  All inpatient outcomes  **Prognostic factor measurement: Mod risk**  CT findings abstracted from CT reports - severity assigned by language - not actually used in regression model  **Outcome measures: Moderate risk**  Only inpatient outcomes - possibility of discharge and deterioration  **Confounding factors: High risk**  Patients admitted with CT negative TBI likely to be frail or have other reasons for admission - this will affect outcome measures compared with SAH patients admitted due to +ve CT.  **Statistical techniques: Low risk**  Well presented  Can use for pooling for outcomes; SAH-supports low-risk sub-population |
| Levy et al.  2014  USA | Level 3 rural non-neurosurgical unit in Rocky Mountains  April 2007-Dec 2012  April 2007 patients with small bleeds selectively not transferred to neurosurgical unit  Inclusion criteria:   - Admission GCS13-15 - CT positive intra-cranial injury - Not transferred to neurosurgery unit in accordance with non-transfer policy - CT findings of small SAH - Punctate or minimal contusion - Punctate or minimal intra-cranial bleed - Small SDH, no mass effect   Exclusion criteria:   - Any coagulopathy - Basilar skull fracture or evidence of CSF leak - Extra-dural bleed - Any significant contusion or SDH/intra-cerebral hemorrhage   Review and discussion of CT and patient with neurosurgeon if unsure if should be transferred | Retrospective cohort study  Aim:  Investigate outcomes after a novel non-transfer policy introduced in a small rural trauma unit without neurosurgical cover for mTBI patients with small ICH | Length of stay  Mortality  Neurological deterioration  Neurosurgery  Re-admission in 90 days of discharge  Inter-hospital transfer  Need for repeat CT | No comparison with patients that were transferred | 76/273 patients not transferred  >50% injuries due to skiing/snow boarding  71% patients <55  No patient deteriorated, died, or required neurosurgery or required delayed transfer while admitted to hospital.  2 patients re-admitted within 90 days - 1 patient 6 weeks following admission developed an acute chronic subdural - drained. 1 patient re-admitted with unrelated complaint.  Mean/median GCS = 14.7  Mean/median age = 36  Percent anti-coagulated = 0 | **Study recruitment: Low risk bias**  Retrospective cohort review - reliant on accuracy of written notes  CT inclusion criteria are subjective and patients may have been transferred despite meeting non-transfer policy if clinicians were concerned.  **Attrition: low risk**  **Prognostic factor measurement: Mod risk**  Reliability of case notes - may be incomplete  The definitions of bleed size are subjective.  **Prognostic factors**  N/A  **Outcome measures: Mod risk**  Study dependent on patients re-presenting at the same hospital following discharge if had delayed deterioration.  **Confounding factors: Low risk**  Age affects outcome and size of bleed  **Statistical techniques: N/A**  **General points:**  Small numbers.  No comparator group - need to compare with transferred patients outcomes  Patients not generalizable - v. young and atypical mechanism of injury (mostly winter sports related).  Likely that any patient clinicians felt at risk would have been transferred even if did not meet transfer criteria - no way to check this. |
| Joseph et al. 2013  USA  The acute care surgery model: managing traumatic brain injury without an inpatient neurosurgical consultation | Level 1 trauma center  2009-2011 (likely subset of patients presented below)  Inclusion criteria:   - GCS13-15 - Trauma - Positive findings CT - skull fracture and/or ICH   Exclusion criteria:   - Pre-hospital anti-platelets or anti-coagulants | Retrospective cohort study  Propensity matching 1:2 ratio patients managed solely by trauma surgeons vs. patients who had neurosurgical consultation  Hypothesis:  Trauma surgeons can manage mTBI patients with CT detected intra-cranial hemorrhage without neurosurgical involvement. | Hospital admissions  ICU admissions  Neurosurgical interventions  ED visits after discharge  Mortality  Progression on CT imaging | Age  Sex  Initial GCS  ISS  Head-abbreviated injury score  Neurological examination  CT scan findings -type of skull fracture/type of ICH/size of bleed - reviewed by study investigator | 404 GCS13-15 patients with CT-detected injuries in study period  270/404 used for this study  90/270 - had neurosurgical consultations (NC)  180 - no neurosurgical consultation (no-NC)  Whether neurosurgical consultation requested as discretion of non-specialist surgeon. Propensity matching in this study between 2 groups.  0/270 neurosurgical interventions, hospital mortality, or re-admissions either group  78/90 no-NC and 158/180 NC admitted hospital (*p* = 0.8)  18/90 no-NC and 80/180 NC admitted ICU (*p* = 0.001)  Routine repeat CT 18/90 no-NC, 155/180 NC (*p* < 0.001)  No progression on any repeat CT  8% no-NC and 4% NC group re-attended ED. No re-admissions.  Mean/median GCS = 15  Mean/median age = 30  Percent anti-coagulated = 0 | **Study recruitment: High risk bias**  Subset of patients who meet inclusion criteria selected to facilitate propensity matching. Possible selection out of higher acuity patients as these will have all been referred to a neurosurgeon.  **Attrition: Low risk**  In patient outcomes and documented ED re-attendances - low risk of patients being lost to follow up  **Prognostic factor measurement: Low risk**  All routinely collected clinical data apart from CT imaging, which re-reviewed  **Outcome measures: Mod risk**  Study dependent on patients re-presenting at the same hospital following discharge if had delayed deterioration.  **Confounding Factors: Mod risk**  Does not exclude patients with additional injuries  **Statistical techniques: High risk**  Does not outline how matched groups using propensity scoring  **General points:**  Small numbers  Likely reporting data reported elsewhere |
| AbdelFattah et al.  2012  USA | Level 1 trauma center  Dallas  Texas  Prospective recruitment 2010-2011  Inclusion criteria:   - Adult with ICH (note doesn’t explicitly state secondary to trauma - but implied)   Excluded:   - Age <16 years - GCS <13 - Undergone planned or immediate neurosurgery - Transferred patients | Prospective cohort study  Hypothesis:  Repeat CT imaging in GCS13-15 with ICH, without neurological progression, does not impact the need for neurosurgical intervention.  Patients divided into those 2 groups. Patients with planned repeat CT imaging and those with CT imaging if deteriorated. Allocation by neurosurgeon - no deviation from normal practice. | Outcome measures during hospital admission:  Neurological progression  Medical intervention  Neurosurgical intervention  Repeat CT imaging - worse CT defined as worse by a blinded radiologist/neurosurgeon giving qualitative measure of bleed. | Comparison between groups:  Age  Sex  Coagulation status  Anti-platelets  ISS  GCS | 145 patients met inclusion/exclusion criteria.  92/145 for routine repeat CT  53/145 for CT if deteriorated  Selective group more likely aspirin use, *p* = 0.02  Routine repeat CT worse Head AIS score (*p* < 0.001)  Otherwise groups comparable  5/53 deteriorated and had a repeat CT + 1/53 had repeat scan as started on warfarin  1/145 patients died (due to other injuries)  27/145 radiological deterioration  9/145 patients intubated - states for other injuries  Mean/median GCS = 14.5  Mean/median age = 41  Percent anti-coagulated = 6 | **Study recruitment: Low risk**  Prospective recruitment - states recruited all eligible patients. Doesn’t explain how recruitment occurred.  **Attrition: Low risk**  Follow-up only for period in hospital  **Prognostic factor measurement: Low risk**  Blinded appraisal of CT scans by researcher  **Outcome measures: Mod risk**  No follow-up following discharge - missed delayed outcomes, could have looked for re-attendance  Doesn’t report neurosurgical outcome measures.  **Confounding factors: High risk**  Not isolated head injury - other injuries have clearly affected outcome measures  **Statistical techniques: Low risk**  None  Small study with confounders regarding outcomes |
| Nayak et al.  2013  USA | University Hospital Newark  New Jersey  USA  Level 1 trauma center  2003-2008  Inclusion criteria:   - Age ≥18 years - Blunt trauma - Intra-cranial bleed - Admitted to hospital - GCS13-15 on arrival to ED - GCS 15 24 h after attendance to ED   Excluded:   - History brain disease, e.g., dementia - Previous brain injury, e.g., CVA - Liver cirrhosis, renal disease, coronary artery disease, bleeding or clotting disorder - Unable to assess GCS due to drugs, e.g., sedation/intubation - Neurological deterioration leading to repeat CT - Age <15 years - Incomplete notes | Retrospective chart review  Aim:  To compare neurological outcomes of MHI patients with an intra-cranial bleed with a normal neurological examination managed with and without a repeat CT head scan | Neurosurgical intervention after 24 h - craniotomy, ventriculostomy, ICP bolt/measurement  Death in hospital  Discharge disposition  LOS hospital  GOS at follow-up clinic/re-attendance if applicable | Age  Sex  Mechanism of Injury  GCS on arrival  ISS  HAIS  GCS and neurological examination every 2 h - routine care on a flow sheet | 321/864 patients GCS13-15 with ICB met inclusion criteria  20% excluded because incomplete medical notes/transfers  0/321 neurosurgical intervention - all within 24 h of admission  No deaths  19/142 worse CT on repeat CT after 24 h of admission  179/321 single CT  142/321 routine repeat CT  76/321 returned to follow-up clinic - uneventful  14/321 returned to ED due to symptoms.  Mean/median GCS = 14.9  Mean/median age = 41 | **Study Recruitment: Low risk**  Retrospective case note review - depends on information being recorded correctly  **Attrition: Mod risk**  20% excluded because of incomplete notes  **Prognostic factor measurement: Mod risk**  Neuroradiology reports taken at face value - no verification  **Outcome measures: Mod risk**  No uniform follow-up of patients post-discharge. Some patients had follow-up clinic, others didn’t. Patients may have presented after discharge to other sites.  **Confounding factors: Low risk**  None obvious  **Statistical techniques: Low risk**  None completed  The inclusion/exclusion criteria have selected out all patients that are not GCS15 at 24 h. Different population than all GCS13-15 patients with TBI on CT - probably unable to pool these data.  Does show patients who are GCS15 at 24 h are low risk. |
| Anandalwar et al.  2016  New Jersey  USA | University Hospital  Newark  New Jersey  USA  Level 1 trauma center  2009-20012  Inclusion criteria:   - Age ≥18 years - Blunt trauma - Intra-cranial bleed/skull fracture - Admitted to hospital - GCS13-15 on arrival to ED - GCS15 24 h after attendance to ED - Did not receive a repeat CT head scan   Excluded:   - History of neurological or psychiatric disorder - Immediate neurosurgery - Previous TBI or neurosurgery - Spinal injury - Coagulopathy - Pregnancy - Transfers - Incomplete notes   Patients who did undergo a repeat CT scan despite meeting the rest of inclusion/exclusion criteria formed a comparison group. | Retrospective cohort study  Aim:  Assess the outcomes following the implementation of a policy of observation only (no repeat CT imaging) for GCS15 patients | Repeat CT after 24 h of admission due to clinical concern or deterioration  Progression on any repeat CT completed  Neurosurgical interventions  Intubation, ICU admissions, administration of mannitol  ED revisits within 1 year for TBI-related symptoms | Age  Sex  Mechanism of Injury  ISS  AIS | 533 patients TBI and ICH  142 met the inclusion/exclusion criteria  47 underwent a routine repeat CT within 24 h (violation of policy) - 0/47 neurosurgical, 1/47 had incidental finding on CT  95 no repeat routine CT within 24 h  8/95 (non-violation group) had repeat CT >24 h after admission - due to concern.  3/8 progression on CT  1 neurosurgical intervention  2/8 admitted to ICU due to deterioration - 1 intubated  3/95 patients returned with 1 year to the ED due to TBI symptoms - all underwent repeat CT. No admissions.  Mean/median GCS = 14.8  Mean/median age = 38  Percent anti-coagulated = 0 | **Study recruitment: High risk**  Patients at GCS15 at 24 h - low-risk group selected out - difficult to extrapolated to all GCS13-15 patients  Does not compare outcomes in patients who adhered to and violated non-routine repeat CT head imaging. Potentially clinicians ordered routine repeat CT imaging on riskier patients.  **Attrition: Low Risk**  Potential for patients to have re-attended at other EDs and be missed  **Prognostic factor measurement: Low risk**  No risk model developed  Factors abstracted from case notes  **Outcome measures: Low risk**  Re-attendance at other EDs makes re-attendance a potentially biased outcome measure.  **Confounding Factors: Mod risk**  Cohort includes patients with multiple injuries  **Statistical techniques: Low risk**  None presented  Is a lower-risk population due to selection for repeat CT imaging and return to GCS15 at 24 h - possibly unable to include in any meta-analysis |
| Ditty et al.  2015  Alabama  USA | University of Alabama  Level 1 trauma center  2003-20013  Inclusion criteria:   - 500 consecutive patients present on trauma registry - GCS13-15 - ICD-9 diagnosis SAH and/or intra-parenchymal contusion - confirmed with radiology report and neurosurgical consult note - if disagreement scan re-reviewed, if not clear patient excluded   Excluded:   - Diagnosis extra or subdural hematoma - Penetrating injuries - Fatal extra-cranial injuries - CSF leak - Aneurysmal SAH - Delayed presentation | Retrospective cohort study  Aim:  Assess the clinical implications of SAH or intra-parenchymal hemorrhage in mTBI | Neurological decline - altered mental state or focal neurological deficit  Inpatient seizure  Delayed neurosurgical evacuation as inpatient  Inpatient mortality | Admission GCS  Anti-coagulation  Anti-platelets  Transfer distances  Sex  Age  Hemorrhage type | 500 patients met inclusion criteria  411/500 isolated SAH  63/500 isolated ICH  26/500 both  463 GCS15  30 GCS14  8 GCS13  469/500 patients pre-hospital medication available (71/469 taking either anti-coagulants or anti-platelets)  156/500 transfers  No patients had seizures.  No patients had neurological decline.  No patients underwent delayed neurosurgical intervention.  No inpatient mortality | **Study recruitment: Mod risk**  High proportion of transferred patients may represent higher or lower acuity patients than general population.  Higher as being transferred to specialist center, lower as survived/fit to transfer.  No details about inclusion or completeness of trauma registry  **Attrition: Low risk**  Only inpatient measures  **Prognostic factor measurement: Mod risk**  Incomplete information regarding medications  May be other inaccurate recording of factors  **Outcome measures: Mod risk**  Only inpatient-related outcome measures. Patients may have been discharged and deteriorated and presented to other hospitals.  **Confounding Factors: Mod risk**  Cohort includes patients with multiple injuries - only excluded if died from other injuries.  **Statistical techniques: N\|A**  None presented  Narrative synthesis - further evidence SAH low risk |
| Pruitt et al.  2016  Chicago  USA | Level 1 trauma center  Chicago  2009-2013  Inclusion criteria:   - Initial GCS13-15 - Age ≥16 years - Traumatic intra-cranial bleed or skull fracture - Identified on electronic ED system using ICD-9 classification system - Admitted to ED observation unit   All patients received a neurosurgical consultation. | Retrospective cohort study  Aim:  Assess if mTBI patients with intra-cranial hemorrhage can be managed to an ED observation unit | Clinical deterioration (defined as decrease in mental status, worsening neurological exam or death)  Neurosurgery during admission  Progression on CT | Age  Gender  Method of arrival  Whether transfer  Comorbidities  Anti-coagulant use  Mechanism of injury  Initial GCS  Neurological examination  Alcohol intoxication  Initial platelet count INR  Initial CT results  Follow-up CT results, Neurosurgical recommendations  Cranial CT data were collected from attending radiologist reports - type and size of detected injury | 1185 GCS13-15 with CT-detected injuries  814 admitted directly to hospital - polytrauma, social reasons, or as neurosurgeons felt high risk.  371 left under care of ED. Of these, 239/371 transferred ED observation unit. 132/371 discharged directly from the ED after a period of observation.  **Admitted patients**  Clinical deterioration  15/814  Worsening CT  27/814  Neurosurgery  33/814  Composite outcome 75/814  **ED observation unit**  Clinical deterioration  0/239  Worsening CT  11/239  Neurosurgery  3/239  Composite outcome 14/239  Medical admission 4/239  Trauma/neurosurgery admit 8/239  **Follow-up 190/239**  Delayed Neurosurgery  0/239  Post-traumatic seizure 3/239  Concussive symptoms 16/239  **Discharged ED**  **Follow-up 111/132**  Delayed Neurosurgery  1/132  Post-traumatic seizure 2/132  Concussive symptoms 8/132  Figures from table - author has confirmed this is correct:  *155 isolate SAH - 0 no clinical or radiological deterioration or cases of neurosurgery.*  *161 SDH - 6 CT deterioration*  *3 planned neurosurgical outcomes*  *0 deteriorated clinically*  *1 neurosurgery >3 weeks later following outpatient assessment*  *30 contusion, 5 worsening CT scans. Nil clinical deterioration or emergency neurosurgery*  *5 extradural - nil deterioration or neurosurgery*  Of sample 1053 mean/median age = 59; 11% anti-coagulated  Of sample 1185 mean/median age = 59; 10% anti-coagulated | **Study recruitment: High risk**  Neurosurgeons have admitted higher-risk patients; we can combine outcomes from both admitted and ED observed patients to give an unbiased estimate.  **Attrition: Med risk**  Only a proportion of patients are followed up - does not describe the mechanism for this or how consistent follow-up is, e.g., did they all get repeat CT scans.  **Prognostic factor measurement: Medium risk**  Dependent on CT scan reports and written documentation  **Outcome measures: Mod risk**  Clinical deterioration not well defined and very broad  **Confounding factors: Low risk**  Included patients with polytauma and significant comorbidities  **Statistical techniques: High risk**  None presented but data presented in table and text do not match up.  Article shows patients admitted to hospital by neurosurgeons have worse outcomes/more likely to require neurosurgery.  Does show that in America some of this patient population discharged directly from ED. Consistent with the model used locally in Hull. |
| Deepika et al. 2013  Bangalore  India | Patients admitted to tertiary neurosurgical center 3 months, Jan-March 2010  Patients identified on a TBI registry  Inclusion criteria:   - GCS13-15 head injury - Underwent CT scan - Either negative CT or Isolated traumatic subarachnoid - Matched comparison between patients - VE CT and SAH   Excluded:   - Does not state adults only but age range 15-67 | Retrospective cohort study  Aim:  To assess whether GCS13-15 patients with traumatic subarachnoid hemorrhage have the same outcomes as mTBI patients with - VE CT scans | Prospective 1-year telephone assessment of :  GOS-E  Rivermead post-concussion questionnaire  Rivermead head injury follow-up questionnaire | Age  Sex  Mechanism of injury-  RTC  Fall  LOC  Seizure  Location of SAH  Whether multiple bleeds  Thickness > or < 5 mm | 34/1628 mTBI patients isolated traumatic subarachnoid hemorrhage  18/34 patients available for follow-up at 1 year  Good GOS-E  Rivermead scores comparable to 16 normal CT controls | **Study recruitment: Low risk**  Cohort identified in TBI registry, which is part of normal practice.  Is retrospective so limited by accuracy of medical notes.  **Attrition: High risk**  Small sample - with large proportion lost to follow-up  **Prognostic factor measurement: Medium** **risk**  Dependent on CT scan reports and written documentation  **Outcome measures: High risk**  1 year too long  **Confounding factors: Medium risk**  No control for other injuries or comorbidities  **Statistical techniques: N/A**  Too poor quality to include |
| Kreitzer et al.  2014  Cincinnati  USA | Level trauma center  2001-2010  Identified from cohort of patients who underwent 2 CT within the ED within 24 h  Inclusion criteria:   - GCS14-15 and blunt head injury - Presented within 24 h of injury - Intra-cranial bleed, first CT defined extradural, sundural, SAH, intra-cerebral, and cerebral contusion - 2nd CT within 24 h   Excluded:   - Incomplete notes - Pregnant - Intubated prior to ED evaluation - Abnormal observations - Penetrating injury - CT scans interpreted at different hospital - Coagulopathy either inherited or acquired - INR >1.4 (even if taking warfarin) - Platelets < 50 - Any non-head injury mandating admission - Age < 18 years | Retrospective cohort study  Standard practice repeat CT at least 6 h after 1st CT if mTBI with ICH. If CT and patient stable discharge from ED.  Aim:  Assess outcomes for patients with mTBI and ICH | Death within 30 days  Neurosurgical intervention within 2 weeks  Return to the Ed within 7 days of discharge | CT head findings  Age  Race  Sex  Medical background | 323/1011 patients who underwent 2 CT head scans within 24 h in ED met the inclusion criteria.  After second CT:  92/323 admitted  25/323 observed in ED and subsequently discharged  206/323 discharged  4 patients died (3 admitted, 1 discharged). States death in discharged patient unlikely to be related to head injury, had further fall. Also 1 other patient died of septic shock.  3 neurosurgical interventions (all admitted)  28/206 discharged patients returned to ED within 1 week. None re-admitted and some planned - removal of sutures.  Mean/median age = 42  Percent anti-coagulated = 0 | **Study recruitment: Mod risk**  Identified through repeat CT imaging in ED - relies on all of cohort having repeat scans and patients deteriorated and not undergoing second scan being missed.  **Attrition: Low risk**  Followed-up through social security system for deaths and the rest are inpatient outcome. Possibility of patients re-attending at other ED.  **Prognostic factor measurement: Medium** **risk**  States that some CT are reported by radiology trainees overnight and then corrected by attending radiologists the next day - unable to quantify how much inaccuracy there is.  Does state 32% of repeat scan normal.  **Outcome measures: Low risk**  Reasonable outcome measures  **Confounding Factors: Low risk**  Controls for comorbidities and other injuries  **Statistical techniques: N/A** |
| Ding et al.  2012  Neurosurgical center  China | Neurosurgical center  China  2009-2010  Inclusion criteria:   - All patients with TBI with evidence of intra-cranial hemorrhage - some data for GCS13-15   Excluded:   - Immediate neurosurgery - Died within 3 days - Severe multiple injuries - Failed to undergo a repeat CT head | Appears to be a random control trial comparing outcomes in patients with traumatic intra-cranial hemorrhage assigned either to a routine repeat CT or CT only if deteriorates | GCS at discharge  Surgical and medical interventions secondary to CT | CT scan results  Initial GCS  Mechanism of Injury  Coagulation INR and platelets | 32/89 patients in routine CT group GCS13-15  2/32 worse CT scans  No patients had neurosurgery or altered medical management  Mean/median age = 48 | **Study recruitment: High risk**  Allocation to intervention and non-intervention arm not clearly explained - states via random number generator  **Attrition: Low risk**  Low risk - inpatient outcomes  **Prognostic factor measurement: Medium** **risk**  No re-reporting of CTS  **Outcome measures: Medium risk**  No outcome measures after discharge  **Confounding factors: Low risk**  Controls for other injuries  **Statistical techniques: N/A** |
| Huynh et al.  2006  USA | Level 1 trauma center  2004-2005  Identified case note review  Inclusion criteria:   - mTBI - Blunt trauma to head - GCS15 - Abnormal CT head   Excluded:   - Normal initial CT head - Length of admission < 48 h - Age < 18 years | Retrospective cohort study  Aim:  To assess whether neurosurgical review is necessary in GCS15 patients with intra-cranial injuries | Changes on follow-up CT - all patients had routine repeat CT  Neurosurgical intervention | Demographics  Mechanism of Injury  ISS  LOC  Amnesia  Associated injuries | 56 patients met inclusion criteria  4/56 patients worse repeat CT  Of these 4:  2/56 patients had fall in GCS to 14 from 15  1/56 given mannitol due to worse CT  1/56 loaded with phenytoin for seizures  No consistent measure of deterioration  0/56 neurosurgical interventions  0/56 deaths  Mean/median GCS = 15  Mean/median age = 41 | **Study recruitment: Medium risk**  Weaknesses of a retrospective case note review  Higher risk group as admitted for at least 48 h  **Attrition: Low risk**  Low risk - inpatient outcomes  **Prognostic factor measurement: Medium** **risk**  No re-reporting of CTS  **Outcome measures: Medium risk**  No outcome measures after discharge  **Confounding factors: Low risk**  No controls for other injuries  **Statistical techniques: N/A** |
| Almenawer et al.  2013  Ontario  Canada | Neurosurgical center  Ontario  Canada  2006-2011  Identified from trauma database  Inclusion criteria:   - GCS13-15 - Blunt traumatic head injury - Age >17 years - Intra-cranial injury CT head - Repeat CT scan   Excluded:   - No repeat CT scan - Previous craniotomy - Cranial pathology - Coagulopathy - Immediate neurosurgery   Patients divided into those who underwent intervention due to clinical deterioration or due to repeat CT findings | Retrospective cohort study + meta-analysis to assess whether repeat CT imaging necessary in mTBI with intra-cranial hemorrhage | Intervention including:  Mannitol or hypertonic saline  Surgical intervention including ICP bolt or craniotomy  Neurological changes: decrease GCS, cranial nerve change, vomiting and headache | Demographics  GCS  ISS | 1121 patients with mTBI and ICH  445 met inclusion criteria  91/445 worse CT  21/445 patients neurosurgical outcomes (all preceded by clinical deterioration prior to repeat CT)  4/445 patients medical intervention  2/4 medical outcomes = treated with mannitol due solely worse CT, other 2 treated due to clinical deterioration  Mean/median GCS = 14.5  Mean/median age = 45  Percent anti-coagulated = 0 | **Study recruitment: High risk**  Dependent on accuracy of trauma database  Large proportion of mTBI patients with ICH did not meet inclusion criteria - selection out of higher risk patients who did not undergo repeat imaging  **Attrition: Low risk**  Low risk - inpatient outcomes  **Prognostic factor measurement: Medium** **risk**  No re-reporting of CTS  **Outcome measures: Medium risk**  No outcome measures after discharge  **Confounding factors: Low risk**  No control for poly trauma  **Statistical techniques: N/A** |
| Sifri et al.  2004  USA | Level trauma center  New Jersey  USA  1999-2001  Inclusion criteria:   - GCS14-15 - Blunt traumatic head injury - Age >15 years - Intra-cranial injury CT head - Repeat CT   Excluded:   - History of brain injury - Coagulopathy including known bleeding disorder or taking warfarin - Immediate neurosurgical intervention including transfer to ICU | Retrospective cohort study:  To assess the value of routine repeat CT imaging in mTBI patients with intra-cranial hemorrhage | Worse CT  Inpatient neurological deterioration - abnormal neurology - confusion, disorientation or drowsiness  Inpatient neurosurgical interventions | CT results as abstracted from radiologist and neurosurgeons reports  Best ED GCS  Demographics | 243 patients with mTBI and ICH  18/243 excluded as no repeat CT - neurosurgeon ruled insignificant lesion  202/243 included as met the rest of inclusion criteria  At 24 h:  151/202 persistently normal or improving neurology  51/202 persistently abnormal or worsening neurological examination  50/202 worse CT  5/202 required neurosurgery - all had persistent or worsening neurology  1/202 died all in the persistently abnormal/worsening neurology group  No clear measure of deterioration  Mean/median GCS = 14.7  Mean/median age = 44  Percent anti-coagulated = 0 | **Study recruitment: Medium risk**  Selection out of patients not undergoing repeat CT head imaging  **Attrition: Low risk**  Low risk - inpatient outcomes  **Prognostic factor measurement: Medium** **risk**  The definition of abnormal neurology is loose and not clear when it developed - not an admission criteria factor  **Outcome measures: Medium risk**  No outcome measures after discharge  **Confounding factors: Low risk**  No control for polytrauma and comorbidites  **Statistical techniques: N/A** |
| Phelan et al.  2014  Dallas  USA | Level 1 trauma center  Dallas  Texas  USA  2010-2012  Patients identified on TBI database  Inclusion criteria:   - Intra-cranial hemorrhage - TBI - Patients divided into SAH and non SAH bleed - All GCS but data for GCS13-15 patients presented   Excluded:   - Ages < 18 years - Pregnant - Prisoners | Retrospective cohort study  Assess whether outcomes for mTBI with isolated traumatic subarachnoid differ for other kinds of intra-cranial bleeds | Worse repeat CT imaging if any  Death  Craniotomy | CT findings as re-read by a study team member  Age  ISS  HAS  ED GCS | 77 patients GCS13-15 and traumatic SAH  27/77 scheduled repeat CT  3/27 worse CT  50/77 - no routine repeat CT  4/50 - unscheduled repeat CT  1/50 - clinical deterioration and worse CT  4/77 - worse CT  0 neurosurgical intervention | **Study recruitment: Low risk**  Dependent on accuracy of trauma registry  **Attrition: Low risk**  Low risk - inpatient outcomes  **Prognostic factor measurement: Low risk**  Does not really assess prognostic value of factors measured  **Outcome measures: Medium risk**  No outcome measures after discharge  **Confounding factors: Low risk**  No control for polytrauma and comorbidites  **Statistical techniques: N/A** |
| Homnick et al.  2012  New Jersey  USA | New Jersey Medical School  Level 1 trauma center  USA  2002-2005  Inclusion criteria:   - Age >17 years - GCS >12 - TBI with positive initial CT-intra-cerebral bleed, contusion, subdural, extra-dural or SAH   Excluded:   - Penetrating trauma - Injury >24 h previously - Previous neurosurgery - Non-traumatic mass on CT - Immediate neurosurgery | Retrospective cohort study  Establish how long intra-cranial bleeds in mTBI continue to expand | Neurosurgical intervention  Progression on CT - repeat CTs as discretion of neurosurgeon | Age  Sex  Pre-injury anti-coagulation  Mechanism  ISS  Initial GCS | 341 patients in study (85 mTBI patients with bleeds excluded as no follow-up scan)  72/341 intubated in ED  105/341 progression on CT  13/341 death - 9 due to TBI, 4 other causes  12/341 neurosurgical intervention  Mean/median GCS = 14.6  Mean/median age = 47  Percent anti-coagulated = 2 | **Study recruitment: Medium risk**  Selection out of lower-risk patients eho did not have repeat CT imaging  **Attrition: Low risk**  Low risk - inpatient outcomes  **Prognostic factor measurement: Low risk**  Does not really assess prognostic value of factors measured  **Outcome measures: Medium risk**  No outcome measures after discharge  **Confounding factors: Medium risk**  No control for polytrauma and comorbidites  **Statistical techniques: N/A** |
| Nasir et al.  2011  Karachi  Pakistan | Specialist center  Karachi  Non-probability consecutive sampling  Inclusion criteria:   - GCS14-15 - All ages,-15% sample children, mean age 36, 2 SD 18 - TBI with positive initial CT intra-cranial injury   Excluded:   - Clinical deterioration - Immediate neurosurgery - Isolated pneumocephalus   All patients had a repeat CT within 72 h | Retrospective cross-sectional study  Aim:  Assess the utility of repeat CT scanning in mTBI patients with intra-cranial injuries without clinical or neurological deterioration | Worse CT | Age  Gender  Initial GCS  Mechanism of injury  CT findings | 275 patients met inclusion criteria (note states 255 contusion hematoma)  17/275 worse CT  No patients required neurosurgery  Mean/median GCS = 14.7  Mean/median age = 36  Percent anti-coagulated = 0 | **Study recruitment: Medium risk**  Does not adequately define deterioration or over what period  **Attrition: Low risk**  Low risk - inpatient outcomes  **Prognostic factor measurement: Low risk**  Does not really assess prognostic value of factors measured  **Outcome measures: Medium risk**  No outcome measures after discharge  **Confounding factors: Medium risk**  No control for polytrauma and comorbidites  **Statistical techniques: N/A**  **Overall**  Includes children and quite a different population than North America and Europe |
| Boris et al.  2013  Israel | Israel  Level 2 trauma center  Sates 2007-2011  Inclusion criteria:   - GCS14-15 - TBI with positive initial CT intra-cranial injury including subdural, extra-dural, subarachnoid, and intra-cerebral bleeds - Only data for adults presented   Excluded:   - Patients with incomplete data - Transferred to neurosurgery immediately - No repeat CT   All patients had a repeat CT within 12 h | Retrospective cohort study  Assess whether repeat CT imaging in GCS14-15 mTBI with intra-cranial injury justified | Increased size of bleed 2nd CT  Clinical deterioration - decrease in GCS  New motor or sensory symptoms  Severe headache or vomiting | Age  Sex  Initial and follow-up GCS  CT findings | 68 patients  4 patients transferred to neurosurgery (2 routine)  8/68 patients worse CT  12/68 mild deterioration  28 patients intra-parenchymal bleed  1/28 worse CT  3/28 neurological deterioration  1/28 transferred to neurosurgery (not patient with worse CT)  7 patients extra-dural  1/7 worse CT  0/7 neurological change  1/7 transferred to neurosurgery  20 patients subdurals  3/20 worse CT  4/20 neurological deterioration  1/20 neurosurgery  13 patients SAH  3/13 increase in size bleed  5/13 neurological deterioration  1/13 transferred to neurosurgery  Mean/median GCS = 14.8  Mean/median age = 56 | **Study recruitment: Medium risk**  Identified on trauma database with patients with incomplete data excluded. Does not present number of these patients. Also excludes patients transferred immediately. Likely to be lower-risk sample than population of interest.  **Attrition: Low Risk**  Low risk - inpatient outcomes  **Prognostic factor measurement: Low** **risk**  Does not really assess prognostic value of factors measured  **Outcome measures: Medium risk**  No outcome measures after discharge  **Confounding factors: Medium risk**  No control for polytrauma and comorbidites  **Statistical techniques: N/A** |
| Brown et al.  2007  Los Angeles  USA | Los Angeles  Level 1 trauma center  2003-2004  Inclusion criteria:   - All patients with blunt head trauma and intra-cranial bleed initial CT. Presents data for GCS13-15   Excluded:   - Immediate neurosurgery - Died within 24 h - Does not state just adults but seems only for adults (mean age 44 ± 19) | Prospective cohort study  Aim:  To identify patients with head injuries that benefit from routine repeat CT imaging | Need for neurological intervention - either medical or surgical (medical = sedatives, mannitol, or hyperventilation and surgical = ICP monitor and craniotomy)  Mortality | Age  Gender  Mechanism of injury  ISS  Admission GCS  Results of CT - interpreted by attending radiologist | 354 patients all GCS scores with intra-cranial bleed  37 direct to craniotomy  43 died within 24 h  274 = study population  142/274 = mTBI GCS13-15  15/142 had clinical deterioration  27/142 had worse CT scans (only 72/142 had repeat imaging)  5/142 had medical or neurosurgical intervention  3/142 died  Mean/median GCS = 14  Mean/median age = 43 | **Study recruitment: Mod risk**  Removal of patients who died within 24 h may lead to this sample being a lower-risk group than population of interest  **Attrition: Low risk**  Low risk - inpatient outcomes  **Prognostic factor measurement: Low risk**  Does not really assess prognostic value of factors measured  **Outcome measures: Medium risk**  No outcome measures after discharge  **Confounding factors: Medium risk**  No control for polytrauma and comorbidities-  **Statistical techniques: N/A** |
| Thomas et al.  2010  Tennessee  USA | Level 1 trauma center  Tennessee  USA  50 months from Jan 2001  Inclusion criteria:   - All patients with blunt head trauma and evidence TBI on initial CT. Presents data for GCS13-15 - Age 18+ years   Excluded:   - Penetrating mechanism - Immediate neurosurgery - Interventions for unclear indications - Died before 2nd CT   All patients repeat CT at 6-8 h after admission | Retrospective cohort study  To assess whether scheduled repeat CT head imaging is indicated in TBI | Neurosurgical interventions - craniotomy or ICP monitor  Medical interventions - mannitol/hypertonic saline  Neurological change - reduced GCS, pupillary change, increased ICP or loss of brainstem reflexes | Initial GCS  ISS  Race  Age  Gender  Mechanism of injury  History of vascular disease  Anti-coagulant use  Anti-platelet use  PT, aPPT, INR  CT findings | 457/836 in included sample population GCS13-15  14/457 = neurosurgical intervention (craniotomy or ICP bolt)  3/457 medical management  5/14 neurosurgical interventions - based on repeat CT  3/14 medical interventions based on repeat CT  Mean/median age = 42 | **Study recruitment: Mod risk**  Dependent on case note review. Patient with “unclear” indications for interventions removed.  **Attrition: Low risk**  Only inpatient outcome measures  **Prognostic factor measurement: Mod risk**  Does not explain how CT scans reported  **Outcome measures: Mod risk**  No follow-up after discharge  **Confounding factors: Medium risk**  No control for polytrauma  **Statistical techniques: N/A**  None done |
| Klein et al.  2010  Israel | 3 regional trauma centers in Israel. None had access to neurosurgery onsite.  Identified ICD-9 codes on national trauma registry.  Inclusion criteria:   - GCS13-15 - ICD-9 code for intra-cranial bleed.   One hospital transferred all patients to neurosurgical center.  Other 2 hospitals transferred selected patients. | Retrospective cohort study  Aim:  Assess the outcome of low-risk patients with ICB managed in district hospitals without neurosurgical services | Mortality  Neurosurgical intervention  Neurological status at discharge | Age  AIS  ISS | 323 patients all 3 hospital intra-cranial bleed and GCS13-15  27/323 required neuro-rehab  2/323 died  35/323 neurosurgery  77/323 not transferred  0/77 died  0/77 neurosurgery  2/77 delayed transfer  Non-transfer on basis of:  Single bleed ≤5 mm or contusion <1 cm and no-coagulopathy  Mean/median age = 39 | **Study recruitment: Low risk**  Dependent on completeness of trauma registry  **Attrition: Low risk**  Only inpatient outcome measures  **Prognostic factor measurement: Mod risk**  Does not explain how CT scans reported  **Outcome measures: Mod risk**  No follow-up after discharge  **Confounding factors: Medium risk**  No control for polytrauma or comorbidities  **Statistical techniques: N/A**  None done |
| Sifri et al.  2011  USA | Level 1 trauma center New Jersey  USA  2002-2006  Inclusion criteria:   - Initial GCS13-15 - Blunt traumatic head injury - Age 18+ years - Intra-cranial injury CT head - ICB or skull fracture - Repeat CT - Abnormal neurological examination at time of repeat CT   Excluded:   - Immediate or planned neurosurgical intervention - Normal neurology at time of repeat CT - normal neurology defined as GCS15, orientation to place, person or time, normal neurological exam, no symptoms from head injury - headache, vomiting, dizziness, lethargy - Coagulopathy including known bleeding disorder or taking warfarin - Pregnancy - Spinal cord Injury - Prior brain surgery - Acquired or congenital cerebral pathology or existing neurological or psychiatric disorder | Retrospective cohort study  Aim:  To assess proportion of patients who have worse CT scans and neurosurgical interventions who have abnormal neurology when they have a repeat CT | Progression of lesion on CT  Surgical intervention - includes intubation  Medical intervention  GOS-E at discharge | Demographics  Acute deterioration in neurological exam  Persistently abnormal neurological exam  Unknown whether change as intubated | 107 patients met inclusion criteria  63/107 worse CT = 59%  7/107 neurosurgical group  21/107 deterioration  18/107 unable to assess neurology as intubated  6 died  Mean/median GCS = 14.4  Mean/median age = 48  Percent anti-coagulated = 0 | **Study recruitment: High risk**  High-risk subgroup that have abnormal neurology at time of repeat CT imaging  **Attrition: Low risk**  Only inpatient outcome measures  **Prognostic factor measurement: Mod risk**  Difficult to assess deterioration in a retrospective study  **Outcome measures: Mod risk**  No follow-up after discharge  **Confounding factors: Low risk**  Some control for comorbidities  **Statistical techniques: N/A**  None done |
| Beynon et al.  2015  Germany | Heidelberg University Hospital  Germany  2013-2014  Inclusion criteria:   - Initial GCS13-15 - Traumatic Intra-cranial bleed CT head | Retrospective cohort study  Aim:  Compare outcomes in patients on different types of anti-coagulants | Repeat CT imaging  Progression on CT  Neurosurgery  Death  Mean GCS at discharge | Patients divided into those on no anti-coagulants, aspirin, warfarin, and DOACs  Gender  Trauma mechanism  Comorbidities  CT findings  Repeated CT imaging  Age  GCS scores  Laboratory values | 70 patients met inclusion criteria  37 no anti-coagulation  27 anti-platelets  5 warfarin  6 DOACs (rivaroxaban)  1 patient dabigatran  25% neurosurgery (18 patients)  43/70 repeat CT imaging  2 deaths, both on rivaroxaban  Mean/median GCS = 14.5  Mean/median age = 67  Percent anti-coagulated = 16 | **Study recruitment: Low risk**  Although high rates of anti-coagulation  **Attrition: Low risk**  Only inpatient outcome measures  **Prognostic factor measurement: Low risk**  May be miss-classified in medical notes  **Outcome measures: Mod risk**  No follow-up after discharge  **Confounding factors: Low risk**  No control for comorbidities  **Statistical techniques: N/A**  None done |

|  | | | | | | |
| --- | --- | --- | --- | --- | --- | --- |
| *Studies with uni-variate or mult-ivariate risk factors (n = 21)*  *(also included in pooled estimates outcome prevalence)* | | | | | | |
| *Reference* | *Population* | *Study design* | *Outcome measures* | *Prognostic factors assessed* | *Results* | *Quality appraisal* |
| Nishijima et al.  2014  Sacramento  USA | Single-site: Level 1 trauma center  2009-2013  Inclusion criteria:   - Age >18 years - Consecutive patients - Initial ED GCS13-15 - CT +VE ICH - SAH, SDH, EDH, intra-ventricular, intra-parachymal bleed/contusion, diffuse axonal injury   Exclusions:   - Patients with DNACPR - Patients pre-injury anti-coagulant use | Prospective cohort study  Aim:  Derive a clinical decision instrument for patients with mild ICH, low risk requiring critical care intervention.  Statistical method:  Derived clinical decision instrument with binary recursive partitioning (misclassification cost 20:1).  Performance of instrument compared with clinical impression | Critical care invention within 48 h of arrival ED:   - Intubation - Neurosurgery including ICP monitoring/giving mannitol/hypertonic saline - Transfusion RBC/FFP - Vasopressor/ionotrope use - Cardiac arrest/arrhythmia (HR <40, HR >120) - Interventional angiography | Age >65years  Sex  Dangerous mechanism (any non-fall from standing mechanism)  Pre-injury anti-platelet use (aspirin or clopidogrel)  High-risk co-morbidity  ED vital signs  GCS <15 at admission  BP <90 at any point ED  Sats <95% at any point ED  Lab results:  Platelet count  INR  Hematocrit  Initial CT:  Midline shift/absence cisterns  Depressed skull fracture  Non-isolated head injury AIS score ≥3 additional injury | 600 patients  71% male  0.5% died + 6.5% neurosurgery + 8.3% intubated  68% GCS15  93% admitted ICU  19.3% had critical care intervention  9.2% transfusion  8.3% intubation  6.5% neurosurgical  4 predictors need for critical care intervention: (recursive partitioning)  GCS <15 (RR 2.95; 95% CI 2.21-4.12)  >65 years (RR 1.46; 95% CI 1.05-2.03)  CT midline shift/absence cisterns (RR 4.11; 95% CI 3.08-5.48)  Non-isolated head injury (RR 2.74; 95% CI 1.99-3.78)  Sensitivity of decision rule to predict intubation/neurosurgery within 48 h of admission ED  98.6% specificity 36.6%  To any critical care intervention  Sensitivity 98.3%, 95% C.I. (93.9-99.5%)  Specificity 39.7%, 95% C.I. (35.4-44.1%)  Positive predictive value 28.1%, 95% C.I. (23.9-32.6%)  Negative predictive value 99%, 95% C.I. (96.3-99.7%)  Clinician impression:  Do you think patient needs ICU?  Sensitivity 90.1%, 95% C.I. (83.1-94.4%)  Specificity 49.2%, 95% C.I. (44.7-53.8%)  Clinical impression deterioration in 48 h?  Sensitivity 91%, 95% C.I. (84.2-95.0%)  Specificity 39.5%, 95% C.I. (35.1-44.1%)  Presence of swelling or shift on initial cranial CT RR (95% CI) 4.11 (3.08-5.48)  Admission GCS score < 15 RR (95% CI) 2.95 (2.12-4.12)  Non-isolated head injury RR (95% CI) 2.74 (1.99-3.78)  Hypotension prior to admission RR (95% CI) 2.70 (1.61-4.54)  Presence of depressed skull fracture RR (95% CI) 2.44 (1.46-4.08)  Presence of any high-risk comorbidity  1.58 (1.07-2.33) RR (95% CI) pre-injury anti-platelet use  1.54 (1.04-2.30) RR (95% CI) hypoxia prior to admission  1.52 (1.03-2.24)  Age ≥65 years RR (95% CI) 1.46 (1.05-2.03)  Non-fall from standing mechanism of injury RR (95% CI) 1.12 (0.80-1.57)  Mean/median GCS = 14.6  Mean/median age = 52  Percent anti-coagulated = 0 | **Study recruitment: Mod risk bias**  Missed 20% eligible patients - not completely clear individuals in cohort identified. Otherwise clear inclusion and exclusion criteria.  **Attrition: Low risk**  Follow-up only 48 h so low risk of attrition bias  **Prognostic factor measurement: Low risk**  Standardized and objective prognostic factor measurement. Collected all patients.  **Outcome measures: Low risk**  Recorded in uniform way for all patients. Only 48 h.  **Confounding factors: Mod risk**  Additional severe injury may be related to prognostic factors and outcome measures. Not accounted for in analysis.  **Statistical techniques: Low risk**  Good presentation of methods  **Overall summary**  Risk factors identified by case note review/d/w treating physicans where not clear. Radiology attending written report used for CT findings. No independent quality verification - could introduce bias. CT end point also missed spectrum of possible findings.  Outcomes out 48 h too short, also critical care intervention definition very broadly - e.g., transfusion. No blinding to exposure/outcomes.  Overall good internal validity of study.  But issues with generalizing results:  Exclusion of anti-coagulated patients.  Short outcome measurement 48 h.  Outcome measures of critical care intervention quite soft - including transfusion of blood products.  No external validation of results. |
| Sweeney et al.  2015  USA | Identified on national trauma database 2007-2012  Inclusion criteria:   - Age >18 years - ED inital GCS14-15 - ICD-9 code intra-cranial injury = cerebral contusion, SAH, SDH, EDH, multiple TBI - Admitted to hospital   Exclusions:   - ICD-9 diagnoses skull fractures - Penetrating mechanism of injury - AIS score >1 any other body region - Data missing ED vital signs | Retrospective cohort study  Hypothesis that injury type associated with deterioration in isolated TBI  Multiple logistic regression used to assess risk of outcomes.  Mixed effects model to explore potential differences between hospitals | Neurosurgical Intervention:  Defined as operative procedure, or placement of an ICP monitor. Identified by ICD-9 coding. | ISS (measure of head injury severity due to exclusion criteria)  Coagulopathy (pooled measure of Vit K deficiency, hemophilia, thrombocytopenia, chronic anti-coagulant therapy) Chronic aspirin use not included.  Type of intra-cranial injury as per ICD-9 code.  ED vital signs  Age | 50,496 patients met criteria  4474/50,496 neurosurg  58% admitted to ICU  EDH, *n* = 901; 18% neurosurg  SDH, *n* = 18,784; 16% neurosurg  Mixed, *n* = 11,984; 8% neurosurg  SAH, *n* = 13,191; 1.5% neurosurg  Contusion, *n* = 5636  Dataset split into 2/3 training set and 1/3 test set.  Adjusted odds ratios for neurosurgical procedures. Multiple logistic regression run on 2/3 training set (*n*  =  33,327)  Age (years) OR = 1.002 (95% CI 0.999-1.01) *p* = 0.18  Anti-coagulation disorder OR = 0.853  (95% CI 0.66-1.09) *p* = 0.21  ED GCS OR = 0.894 (95% CI 0.781-1.03) *p* = 0.11  ED systolic blood pressure OR = 1.004 (95% CI 1.002-1.01) *p* < 0.001  ED Pulse OR = 0.99 (95% CI 0.986-0.993) *p* < 0.0001  ED respiratory rate OR = 0.962  (95% CI 0.944-0.98)  *P* < 0.0001  ISS 7-11 OR = 2.35 (95% CI 1.44-4.09) *p* < 0.01  ISS 12-18 OR = 3.37 (95% CI 2.06-5.86) *p* < 0.0001  ISS 19-27 OR = 18.9 (95% CI 11.6-33) *p* < 0.0001  ISS >27 OR = 7.01 (95% CI 3.79-13.4) *p* < 0.0001  Injury category (vs. contusion)  Isolated SAH OR = 0.95 (95% CI 0.64-1.41) *p* = 0.79  Isolated SDH OR = 4.9 (95% CI 3.61-6.84) *p* < 0.0001  Isolated EDH OR = 6.42  (95% CI 4.15-9.97) *p* < 0.0001  Multiple injury types OR = 2.34  (95% CI 1.7-3.29) *p* < 0.0001  After adjustment injury severity, age, coagulopathy and ED vital signs: injury pattern significantly associated need for neurosurgery:  OR EDH vs. contusion 6.4 (95% CI 4.1-9.9)  Age no association  ED vital signs also predictive  In test AUC ROC curve = 0.81 in test set  Hosmer-Lemeshow *p*  =  0.8 in test set  38% expected and observed rate of neurosurgery highest risk decile. O.5 % in lowest risk decile.  Mean/median age = 61  Percent anti-coagulated = 5 | **Study Recruitment: High risk bias**  Eligible patients recruited through a relatively new national trauma data base by ICD9 coding. Potential selection bias as to which hospitals upload data. Also uncertain how accurate coding is.  Excluded patients with incomplete data, they may be systemically different.  **Attrition: Low risk**  As a trauma registry represents routine information that should be consistent on all eligible patients.  **Prognostic factor measurement: Mod risk**  Grouping of coagulopathy problematic, different likely risk of warfarin vs. ITP for example. CT findings watered down to code for injury, misses important information.  **Outcome measures: Mod risk**  Need for neurosurgery only as recorded on trauma data bank, possibly unreliable. Misses other important adverse outcomes, e.g., death and intubation. Does not include time scale from presentation or what happens to patients who are discharged and re-attend with adverse outcome. Follow-up not clear.  **Confounding factors: Low risk**  Excluded other injuries and made adjustments in logistic regression model. No attempt to control for comorbidities.  **Statistical techniques: Low risk**  Good presentation of methods  Finds that injury type significantly associated with need for neurosurgery -provides candidate factors. There are methodological problems with article. |
| Joseph et al.  2015  USA  Is MTBI defined by GCS: is it really mild? | Level 1 trauma center  Arizona  USA  Retrospective case note review 2009-2012  Inclusion criteria:   - Initial GCS13-15 - Aged 18+ years - Initial scan +VE ICH/skull fracture and routine repeat scan still showed injury - Isolated TBI as defined head AIS ≥3 and AIS <3 other body regions   Excluded:   - On anti-platelets - On anti-coagulants - Transfers - Needed immediate neurosurgery | Retrospective chart review  Aim:  Identify factors that predict progression on CT imaging and neurosurgical intervention in GCS13-15 patients  Method:  All patients underwent routine repeat CT imaging within 6 h of initial CT imaging.  Uni-variate analysis to identify risk factors for progression on CT or neurosurgery  *P* ≤ 0.2 included multi-variate analysis. | Progression on repeat CT  Neurosurgical intervention = craniotomy or craniectomy as inpatient | Age  Gender  Race  Ethnicity  Mechanism of injury  GCS  BP  HR  FBC  Serum lactate  Base deficit  AIS  ISS  CT findings - reviewed by an investigator who was part of the team - classified size of lesion and whether progression on CT | 876 patients met inclusion criteria  115 (13.1%) = progression on CT  Univariate predictors:  Age 65+, *p* = 0.07, OR 1.5 (0.9-2.5)  Male, *p* = 0.8, OR 1.1 (0.6-1.7)  Intoxication, *p* = 0.9, OR 1.3 (0.3-4.7)  Mechanism of injury, *p* = 0.5, OR 1.1 (0.3-2.8)  HR >100, *p* = 0.7, OR1.1 (0.6-1.8)  BP <90, *p* = 0.35, OR 1.3 (0.45-1.9)  LOC, *p* = 0.2, OR 1.2 (0.6-2)  Displaced skull fractue, *p* = 0.02, OR 1.9 (1.1-3.3)  SDH >10 mm, *p* = 0.004, OR 3.4 (1.5-8)  EDH 10 mm, *p* = 0.01, OR3.8 (1.2-7.6)  Hgb <10, *p* = 0.4, OR 1.5 (0.76-3.1)  Platelets < 100,000, *p* = 0.04, OR 1.5 (1.1-3.9)  Lactate ≤2.5, *p* = 0.18, OR2.6 (1.2-5.5) (?!)  Base deficit >4, *p* = 0.02, OR 3.1 (1.2-7.6)  Multi-variate analysis:  Age 65+, *p* = 1.4, OR 1.4 (0.7-2.7)  LOC, *p* = 0.8, OR1.1 (0.5-2)  Displaced skull fracture, *p* = 0.08, OR 2.3 (0.9-3.5)  SDH >10 mm, *p* = 0.007, OR 4.8 (1.9-9.6)  EDH >10 mm, *p* = 0.001, *p* = 7.9 (2.4-12.6)  Platelets < 100,000, *p* = 0.1, OR 1.3 (0.9-3.6)  Lactate ≤2.5, *p* = 0.2, OR 2.1 (0.89-2.5)  Base deficit >4, *p* = 0.01, OR 2.8 (1.6-4.1)  47 (5.4%) = neurosurgery  Uni-variate predictors:  Age 65+, *p* = 0.3, OR 1.08 (0.8-1.3)  Male, *p* = 0.19, OR 1.2 (0.8-1.3)  Intoxication, *p* = 0.3, OR 1.8 (0.9-3.4)  BP <90, *p* = 0.35, OR 1.3 (0.45-1.9)  Mechanism, *p* = 0.34, OR 1.2 (0.4-1.8)  LOC, p = 0.19, OR 1.4 (0.7-3.2)  HR >100, *p* = 0.26, OR 1.5 (0.9-2.8)  Displaced skull fractue, *p* = 0.01, OR 16 (7.6-19.6)  SDH >10 mm, *p* = 0.001, OR 3.9 (2.4-5.1)  EDH >10 mm, *p* = 0.03, OR 4.8 (2.9-5.6)  Hgb <10, *p* = 0.51, OR 1.2 (0.6-2.5)  Platelets <100,000, *p* = 0.31, OR 2.5 (1.15-5.1)  Lactate ≤2.5, *p* = 0.12, OR 3.6 (0.7-6.5)  Base deficit >4, *p* = 0.01, OR 23 (1.6-31)  Multi-variate analysis:  Male, *p* = 0.1, OR 1.6 (0.8-2.1)  LOC, *p* = 0.3, OR 1.2 (0.5-1.9)  Displaced skull fracture, *p* < 0.001, OR 10 (6.7-12)  SDH >10 mm, *p* < 0.001, OR 3.4 (2.1-4.46)  EDH >10 mm, *p* = 0.006, OR =3.5 (1.4-5.5)  Platelets <100,000, *p* = 0.09, OR 1.3 (0.98-4.8)  Lactate ≤2.5, *p* = 0.21, OR 1.9 (0.62-3.1)  Base deficit >4, *p* = 0.001, OR 21 (1.6-27)  Mean/median GCS = 14.3  Mean/median age = 54  Percent anti-coagulated = 0 | **Study Recruitment: Mod risk**  Retrospective identification of case notes- depends on accuracy of case notes  Excludes patients on anti-coagulatants and anti-platelts  **Attrition: low risk**  Outcomes only as inpatients  **Prognostic factor measurement: Low risk**  Relies on accuracy of medical notes.  Re-examines CT images  **Outcome measures: Mod risk**  Only measures as inpatient. Potential for discharge and deterioration.  **Confounding Factors: low risk**  Possibility of confounding due to other comorbidities- does not adjust for this,  **Statistical techniques: Mod risk**  Some of the results appear to be reported wrong. E.g. Lactate  **Overall**  Presents useable data for analysis  **Note base deficit found to be highly prognostic- only study to assess this.** |
| Borczuk et al.  2013  USA | Level 1 trauma center  Boston  USA  Case note review 2009-2010, patients identified through ED electronic coding, ICD-9 coding for intra-cranial hemorrhage.  Inclusion criteria   - GCS13-15 - Age 15+ years - CT positive traumatic intra-cranial hemorrhage   Excluded:   - Isolated skull fractures | Described as a cross-sectional study  Seems more like a retrospective cohort study  Aims:  Develop a set of criteria to identify patients who are at low risk for deterioration and thus may not require neurosurgical evaluation  Method:  Uni-variate analysis to predict composite outcome of deterioration  3 factor multi-variate model derived from uni-variate analysis | Deterioration while in hospital including:  Decrease in GCS  Worsening neurological examination  Worsening CT result on repeat CT  Neurosurgery  Death  Composite outcome  All outcomes while in hospital - no discharge outcomes | Data extracted from case notes by 2 ED researchers. Not blinded to the hypothesis  Age  Method of arrival  History of HTN  Anti-coagulation  Mechanism  Initial GCS  Neurological examination  Alcohol Intoxication  Initial platelet count  INR  Initial CT result  Follow-up CT result  CT categorized by attending radiologist - type, location, and size of bleed/contusion. Presence of midline shift | 404/863 TBI patients met inclusion criteria (46.8% patients with traumatic bleeds).  11.8%(48) deteriorated  5.9% neurosurgical  Deterioration stratified by injury:  24/136 isolated SDH  0/1 isolated EDH  1/75 isolated SAH  2/31 contusions  22/161 mixed lesions  Uni-variate predictors of deterioration:  Age 65+, OR 0.93, 95%CI 0.5-1.69  Sex, OR 0.77, 95%CI 0.41-1.41  Fall, OR 0.57, 95%CI 0.29-1.09  Assault, OR 1.07, 95% CI 0.45-2.51  RTC, OR 0.51, 95%CI 0.12-2.21  Pedestrian struck, OR 1.12, 95% CI 0.32-3.92  Bicycle struck, OR 1.51, 95%CI 0.42-5.44  HTN, OR 0.94, 95% C.I. 0.51-1.73  Aspirin, OR 0.79, 95% CI0.41-1.51  Warfarin, OR 0.87, 95% CI 0.33-2.32  Clopidogrel, OR 1.25, 95% CI 0.27-5.75  GCS <15, OR 2.12, 95% CI 1.01-4.43  CT findings  Any lesions  SDH, OR 2.64, 95% CI 1.20-5.83  EDH, OR 2.4, 95% CI 0.91-6.31  SAH, OR 0.42, 95% CI 0.22-0.81  Contusion, OR 0.79, 95% 0.39-1.62  Isolated lesions  SDH, OR 1.62, 95% CI 0.88-2.96  EDH, OR only 1 patient  SAH, OR 0.078, 95% CI 0.01-0.59  Contusion, OR 0.46, 95% 0.11-1.96  Multiple logistic regression with 3 variables GCS = 15, presence SDH and presence isolated SAH:  All remained significant predictors of deterioration. Sensitivity 97.9% and specificity 20.8%  Negative predictive value 99.6%  Positive predictive value 38.8%  Mean/median GCS = 14.8  Mean/median age = 60  Percent anti-coagulated = 10 | **Study Recruitment: low risk**  Dependent on how good electronic coding is and case note review was.  **Attrition: Low risk**  Follow up only for period in hospital  **Prognostic factor measurement: Low risk**  Written CT reports from attending radiologist used for data extraction. No verification of accuracy or consistency.  **Outcome measures: Mod risk**  No F/U following discharge- missed delayed outcomes, could have looked for re-attendance.  GCS and neurological examination also potentially subjective.  **Confounding Factors: Mod risk**  No attempt to control or exclude polytrauma patients or patients with multiple comorbidities  **Statistical techniques: Mod risk**  Good univariate analysis  Small number prevented large enough multi-variate model |
| Washington et al.  2012  USA | Level 1 trauma center  Washington  USA  Retrospective case note 2-year period (January 2007-December 2008)  Inclusion criteria:   - Admission GCS score ≥13 - Isolated head injury with no other injury requiring ICU admission - Initial head CT scan positive for any type of ICH - Initial non-operative. management plan   Excluded:   - Patients requiring immediate neurosurgery surgery | Retrospective cohort study  Aim:  To determine if there exists a sub-population of mild TBI patients with an abnormal head CT scan who require neither repeat brain imaging nor admission to an ICU  Standard of care is to admit these patients to ICU and routinely re-CT  Methods:  Uni-variate and multi-variate analysis for outcomes of interest | Neurological or medical decline  The need for neurosurgical intervention  GOS score  Neurological decline was defined as remaining in the ICU or transfer back to an ICU or intervention as a result of a decline in mental status or the development of a neurological deficit.  Medical decline was defined as an increase in monitoring or intervention due to cardiac, pulmonary, or renal decline.  Outcome measures during admission and at discharge | Age  Sex  Injury mechanism  Initial GCS score  Duration of hospital stay  Aspirin/clopidogrel/warfarin use  Ttransfusion of blood products  Intubation  CT scans classified into Marshall and Rotterdam criteria - blinded assessment by author | 321 patients met the inclusion criteria.  Neurological decline 1%, 4  Surgical intervention 1%  Medical decline 6%, 18  Cardiac event 7%  Respiratory event 4%  Seizure event 2%  CT progression  6%  GOS score at discharge:  1: 1%  2: 0%  3: 4%  4: 10%  5: 85%  Age+transfusion predictors of a medical decline (*p* < 0.01).  Odds ratio of having a medical decline after undergoing a blood product transfusion was 12.55 (95% CI 4.3-36.7).  Cardiac and respiratory events: the odds ratios were 5.6 (95% CI 2.4-13.1) and 8.8 (95% CI 2.6-30.4).  Significantly higher mortality transfused group as compared with the non-transfused group (6% vs. 0%, respectively, *p* < 0.0001, Fisher exact test).  Higher rate of brain injury progression in the transfused patients (13% vs. 5%, *p* = 0.04).  Predictors of bleed progression uni-variate analysis:  ICH vol >10 mL, OR 20.13, 95% CI (5.67-71.44)  subfrontal/temporal contusion  OR 5.73, 95% C.I.(2.20-14.89)  age ≥65 years,  OR 4.00, CI (1.40-11.42)  anti-platelet &/or coumadin therapy  OR 2.94, CI (1.12-7.71)  Unclear which other factors assessed.  States: “multivariate analysis was performed, only an ICH volume >10 mL was independently associated with the risk of hemorrhagic progression. Patients with a hemorrhage volume >10 mL were 20.13 times more likely to have progression on head CT."  Mean/median GCS = 14.8  Mean/median age = 57 | **Study Recruitment: low risk**  Through case note review- potential for patients without notes to be missed  **Attrition: low risk**  Follow up only for period in hospital  **Prognostic factor measurement: Low risk**  Case note extraction- potentially incomplete  CT scans re-reported. Uses Marshall classification  **Outcome measures: Mod risk**  Outcome measures only during hospital admission. No measure of re-attendance or community outcome F/U  The outcome measures of neurological and medical decline are subjective.  **Confounding Factors: Medium risk**  No control for other injuries and comorbidities  **Statistical techniques: High risk**  Selective reporting of significant risk factors and does not present full analysis. No analysis to predict neurosurgical outcomes.  Potentially can re-analyse the data from what is presented |
| Choudhry et al.  2013  USA  Identified search strategy | Level 1 trauma center  New Jersey  USA  Retrospective cohort patients in trauma database 2002-2006  Inclusion criteria:   - GCS >12 - Initial scan +VE ICH   Excluded:   - Discharged - Pregnancy - Needed immediate neurosurgery - Spinal cord injury - Brain surgery or existing cerebral pathology - Chronic neurological/psychiatric disorder, e.g., dementia - Incomplete medical records - Use of sedating drugs   Age range 18-90 in results | Retrospective cohort study using trauma database  Objective:  To identify the cause, temporal course, and outcomes of patients who deteriorate neurologically after presenting with MHI and ICH  Methods:  Presents uni-variate and multi-variate risk of death | Outcome measures:  Delayed neurological deterioration defined as:  GCS drop 2 or more points for more than 1 h  New focal neurological deficit  Death  Neurosurgical intervention  Worse CT if performed - worsening in Marshall criteria or significant expansion in volume - neuroradiologist  GOS outcome at 6 months | Collected data:  Age,  Sex  Ethnicity  Mechanism of injury  GCS  AIS  Coagulopathy | 908 patients MHI and ICH  151 not included due to incomplete notes or meeting exclusion criteria.  757 = final cohort  31/757 = delayed deterioration at inpatient. 4.1% (21 due to progression ICH, 10 due to medical causes)  7/757 deaths  21/757 patients worse CT scans  Uni-variate analysis outcome death  Age ≥60, *p* = 0.001  Coagulopathy, *p* = 0.02  Increase Marshall classification repeat CT, *p* = 0.001  Decline in consecutive GCS scores more than 6, *p* = 0.02  Deterioration within 9 h, *p* = 0.04  H-AIS >3, *p* = 0.32  ISS >20, *p* = 0.38  Initial GCS <15, *p* = 0.40  Initial Marshall classification >II, *p* = 0.41  Age >60 predicted deterioration due to expansion of bleed and death in stepwise logistic regression (*p* < 0.01)  Mean/median age = 49 | **Study recruitment: Mod risk**  Retrospective identification of patients on trauma database. Relies on patients being correctly recorded on this. Patients with incomplete notes excluded - may be systematically different.  **Attrition: Low risk**  Reports no loss to follow-up at 6 months routine clinic - may form part of group of patients excluded due to incomplete notes  **Prognostic factor measurement: Low risk**  Relies on accuracy of medical notes  **Outcome measures: Mod risk**  Outcome measure of delayed deterioration - relies on adequate checks on patients and neurological examinations in a consistent way. Assumes this is baseline level of care - likely to vary dependent on where the patients were admitted (e.g., ICU versus normal hospital bed).  **Confounding factors: Low risk**  Doesn’t explicitly say for patients with only a head injury, if does include other injuries high risk for confounding.  Also no adjust for comorbidities  **Statistical techniques: High risk**  Uni-variate outcomes for mortality presented only as *P* values.  Performed multi-variate stepwise regression - for mortality reports only one result without confidence intervals.  **Overall**  Compares patients with medical and neurosurgical deterioration and who died and didn’t die with worsening CT scans. Much more pertinent to compare patients who deteriorated and didn’t deteriorate. |
| Kim et al.  2014  South Korea | University hospital  Seoul  South Korea  Case note review from Jan 2002-Dec 2012  Inclusion criteria:   - All patients with acute traumatic subdural bleeds   Excluded:   - Neurosurgery within 24 h of admission - GCS <13 on admission - Patients with vascular abnormalities - Subdural localized to the falx/tentorium cerebelli - Bilateral subdurals - Aged <15 years - Other significant injuries - Patients refusing surgery | Retrospective chart review  Aim:  To determine risk factors with delayed subdural enlargement leading to surgery in patients with acute subdurals | Delayed surgical evacuation of subdural hematoma | Age  Gender  Cause of trauma  Presence of other CT findings  GCS  Neurological deficit  Comorbidities  History of anti-platelets  Anti-coagulation therapy  INR  Platelet count | 98 patients included  51/98 progression on CT either at 1 week , 2 weeks, or 3-10 weeks  34/98 delayed surgical evacuation up to 10 weeks following trauma  Uni-variate comparison between conservative and delayed neurosurgical group:  Mean age, *p* = 0.375  Male, *p* = 0.950  Glasgow Coma Scale, *p* = 0.647  Hypertension, *p* = 0.883  Diabetes, *p* = 0.785  Smoking, *p* = 0.107  Alcohol abuse, *p* = 0.840  Use of anti-coagulant, *p* = 1.000  Use of anti-platelet agent, *p* = 0.546  Thrombocytopenia (<50,000), *p* = 1.000  Prolonged prothrombin time (INR >1.4), *p* = 0.656  Cause of head trauma, *p* 0.651:  Fall from standing  Motor vehicle accident  Fall from a height  Assault  Bicycle accident  Mean SDH maximal thickness (mm, range), *p* < 0.001*  Mean SDH volume (mL, range), *p* < 0.001*  Mean midline shift (mm), *p* < 0.001*  Presence of cerebral contusion, *p* = 0.003*  Presence of SAH, *p* = 0.003*  Diffuse cortical atrophy  Mean bifrontal ratio (range), *p* = 0.345  Mean Sylvian fissure ratio (range), *p* = 0.602  Multi-variate analysis of prediction of delayed hematoma evacuation.  Maximal thickness  *P* = 0.527, OR 2.5 (0.5-41.1)  Volume hematoma, *p* = 0.01, OR = 1.1 (1.02 -1.17)  Midline shift, *p* = 0.01, OR = 1.43 (1.09-1.89)  Cerebral contusion, *p* = 0.92 OR 0.85 (0.18-3.97)  SAH, *p* = 0.43, OR 0.53 (0.11-2.56) | **Study Recruitment: Low risk**  Retrospective case note review- depends on information being recorded correctly.  **Attrition: low risk**  All patients appeared to have been followed up appropriately  **Prognostic factor measurement: Low risk**  Appears CTs have been reviewed and volume measurements conducted by member of study team  **Outcome measures: Low risk**  All patients followed up until clinic. No reports of deaths.  **Confounding Factors: Low risk**  None obvious-exclude patients with other injuries  **Statistical techniques: Low risk**  Well presented  **Overall**  Only patients with subdural- have been shown to high risk in other studies.  The neurosurgical rate for these injuries appears v. high ?length of follow up. These patients have been discharged and then undergone reimaging as outpatients. Doesn’t preclude early discharge of some of these patients but they will need to be followed up. |
| Overton et al.  2014  USA  Can trauma surgeons manage mild traumatic brain injuries?  Journal: American Journal of Surgery | Level 1 trauma center  USA  2006-2012  Inclusion criteria:   - Intra-cranial bleed <1 cm - to hospital - GCS13-15 on arrival to ED   Excluded:   - Multiple injuries on CT - Transferred to other care facility - Left against advice   Doesn’t state only adults but results presented only for adults. | Retrospective cohort study  Aim:  Reports initial experience with the management of MTBI by trauma surgeons alone. Hypothesizes that patients with MTBI managed by trauma surgeons will be the same as outcomes for patients managed by neurosurgeons. | Outcome measured GOS score at discharge  1 = death  2 = severe disability  3 = mod disability  4 = full recovery  Method:  Multi-variate regression analysis to assess whether admission under trauma surgeons affected likelihood of GOS >3 (good recovery) | Trauma versus neurosurgical management  Age  Sex  Race/ethnicity  Injury severity  Insurance status  GCS | 171 patients  8 deaths  4 severe disability  24 moderate disability  Neurosurgeons managed 120  Trauma surgeon 51  Multi-variate regression analysis to predict GOS >3 (full recovery)  Admission trauma surgeon, *p* = 0.3, OR 1.74 (0.61-4.92)  Age, *p* < 0.001,  OR 0.94 (0.91-0.96)  ISS, *p* < 0.001, OR 0.87 (0.81-0.94)  GCS, *p* = 0.005, OR 13.96 (2.23–87.3)  Other factors in model but no results reported: sex, ethnicity, ISS, insurance status  Mean/median GCS = 14.7  Mean/median age = 49 | **Study recruitment: Mod risk**  Retrospective case note review - depends on information being recorded correctly.  Only patients with bleed <1 cm  **Attrition: Mod risk**  Not clear when outcomes measured - if at discharge low risk  **Prognostic factor measurement: Low risk**  Doesn’t explain how CT reports interpreted and how 1-cm cut off decided.  **Outcome measures: Mod risk**  States GOS - but not when or who determined score ?self-reported  **Confounding factors: Mod risk**  None obvious  **Statistical techniques: Mod risk**  States backward step binary logistic regression analysis performed to assess trauma surgeon vs. neurosurgical admissions - controlled for age, sex, race, ISS, insurance status, and GCS motor scores - presents the analysis for only some of these.  **Overall**  Limited by inclusion criteria of <1 cm and even though no difference in outcomes with who patients were admitted under, potentially the patient groups received different care. |
| Schwed et al.  2016  California  USA | UCLA California  Level 1 trauma center  USA  2012-2015  Inclusion criteria:   - Patients identified on trauma registry and case note review - Initial GCS13-15 - Intra-cranial bleed any variety identified by CT imaging   Excluded:   - Transfers - Not admitted to ICU - Required emergent neurosurgery - Patients <18 years - In police custody - Pregnant | Retrospective cohort study  Aim:  Identify admission variables associated with favorable outcomes with mTBI and intra-cranial hemorrhage  Method:  Uni-variate and multi-variate regression analysis prediction of “favorable outcome composite measure” | Favorable outcome **-** composite outcome of following:  Alive at discharge  ICU admission for <24 h  No in hospital complications  Did not require neurosurgery  Failed to achieve this if required ventilation or ionotropic support at any point | Vital signs  AIS  ISS  CT findings - Marshall and Rotterdam scores | 380 TBI patients in study period  19 missing records  201 remaining cohort met inclusion/exclusion criteria  4/201 deaths (2 attributable to bleed progression)  129/201 GCS15  6/201 neurosurgical outcomes  21% (42) in hospital complication  78/201 = met conditions favorable outcome  0/1 EDH favorable outcome  1/4 ICH favorable outcome  18/36 SDH favorable outcome  30/57 SAH favorable outcomes  22/83 mixed lesions favorable outcome  123/201 = unfavorable outcome  Uni-variate comparison between patients with favorable and unfavorable outcomes:  Age, *p* = 0.01  ISS, *p* = 0.001  Head AIS, *p* = 0.026  Time to first head CT (hours) non-significant  ED systolic blood pressure, *p* = 0.01  ED heart rate, *p* = 0.48  Marshall score, *p* = 0.11  GCS at time of admission ICU, *p* < 0.0001  GCS15 at admission, *p* = 0.0001  Type of hemorrhage  Epidural, *p* = 0.42  IVH, *p* = 0.55  SDH, *p* = 0.1  SAH, *p* = 0.02  Combination, *p* = 0.002  All factors statistically significant in uni-variate analysis were assessed in multi-variate analysis.  Multi-variate model predicting favorable outcome: including ED BP, Marshall score, Isolated SAH, Head AIS, ISS <25, GCS15 at ICU admission and age <55  GCS15 at ICU admission, OR 5.5, 95% CI (1.6-18.8), *p* = 0.006  Isolated SAH, OR 5.1, 95% CI (1.5-17.6), *p* = 0.01  Age <55, OR 3.5, 95% CI (1.1-11.2), *p* = 0.03  Mean/median age = 60 | **Study recruitment: Mod risk**  Only admitted to ICU - higher risk group than total population  **Attrition: Low risk**  Only inpatient measures  **Prognostic factor measurement: Mod risk**  Does not assess pupillary response or anti-coagulation/anti-platelets  **Outcome measures: Mod risk**  Only inpatient-related outcome measures.  **Confounding factors: Mod risk**  Cohort includes patients with multiple injuries - 2 deaths appear due to factors unrelated to head injury  **Statistical techniques: Mod risk**  Selective reporting of significant results.  Does present statistical comparison between the groups with favorable and unfavorable outcomes. |
| Thorson et al.  2012  Miami  USA | Level 1 trauma center  Miami  USA  1996-2010  Inclusion criteria:   - Initial GCS13-15 - Present on trauma registry - Head abbreviated AIS 1 or greater - No other injuries (AIS = 0 other body regions) - Repeat CT head scan if intra-cranial injury detected. (4-6 h after initial CT). Note neurosurgeons decided whether a lesion was too insignificant to warrant a repeat CT   Excluded:   - Penetrating trauma - Pregnant - Age <18 years - Incarcerated - Transfers | Retrospective cohort study  Aim:  To test whether routine CT imaging in mTBI with detected intra-cranial injuries provides useful information in the absence of neurological deterioration  Methods:  Step-wise multi-variate regression for factors *p* < 0.2 associated with progression on CT and craniotomy | Progression of initial lesion or new lesion identified  Neurosurgical intervention  Death | CT findings - including type of injury, presence of edema, mass effect or herniation  Age  Sex  ISS  GCS  Abnormal neurological examination - change in GCS >1, GCS <13, neurological deficit, or significant symptoms including headache, lethargy, visual disturbance | 1510 patients with GCS13-15 and head injury  537/1510 +VE initial CT scans  62 proceeded immediately to surgery and 115 no repeat CT in 24 h - (mostly as the neurosurgeon deemed injury insignificant).  360/537 had repeat CT imaging.  11% of repeat CT scans - recalled (i.e., no actual injury).  108/360 - progression on CT imaging  Mean/median GCS = 14.5  Mean/median age = 47  Percent anti-coagulated = 3  Age: no change 46, SD 20, progression 50, D 23, *p* = 0.13  Sex: no Change, male 178, progression 79, *p* 0.11  Intubated: no change 22, progression 17, *p* = 0.05  ISS: no change 12, SD 5, progression 15, SD 6, *p* < 0.01  GCS 15 arrival: no change 158, progression 37  GCS 14: no change 65, progression 43  GCS 13: No change 31, progression 28  Anti-coagulant use: no change 17, progression 11, 0.29  Aspirin: no change 7, progression 3  Plavix: no change 1, progression 2  Coumadin: no change, 2 progression 4  LMWH: no change 2, progression 0  Multiple: no change 5, progression 2  PT: no change 12.2, progression 12.6, *p* = 0.443  PTT: no change 25.2, progression 24.8, *p* = 0.85  30/360 neurosurgical outcomes  Age: no neuro surg 47, SD 21, neuro surg 51, D 23, *p* = 0.97  Sex: no neuro surg male 241, neuro surg 22, *p* 0.11  ISS: no neuro surg 13, SD 5, neuro surg 17, SD 6, *p* < 0.01  GCS15: arrival: neuro surg 180, neuro surg 13  GCS14: no neuro surg 100, neuro surg 8  GCS13: no neuro surg 50, neuro surg 9  Anti-coagulant use: no neuro surg 22, neuro surg 6, 0.024  Aspirin: no neuro surg 9, neuro surg 3  Plavix: no neuro surg 2, neuro surg 2  Coumadin: no neuro surg 2, neuro surg 4  LMWH: no neuro surg 2, neuro surg 0  Multiple: no neuro surg 4, neuro surg 2  PT: no change 12.1, progression 12.0, *p* = 0.35  PTT: no change 25, progression 27.5, *p* = 0.45  7/30 operated patients solely on basis of worse CT (no prior neurological decline)  22/360 deaths  Logistic regression analysis: unclear which factors were tested in the model  Predictors of worse 2nd CT, AU ROC curve 0.703  GCS = 13, OR 4, 95% CI 2.02-7.93, *p* < 0.001  GCS = 14, OR 3.11, 95% CI 1.77-5.48, *p* < 0.001  ISS, OR 1.07, 95% CI 1.02-1.11, *p* < 0.001  Mass effect, OR 2.02, 2.02-3.78, *p*< 0.001  Predictors of craniotomy: AUC ROC 0.849  Initial mass effect, OR 5.24, 95%CI (1.96-14.1), *p* = 0.001  New/worse EDH 2nd CT, OR 23.3, 3.67-148.3, *p* = 0.001  New/worse mass effect 2nd CT, 5.73, 95% 1.64-20  New/worse herniation, 32.1, 95% CI 7.83-131.6, *p* = 0.001 | **Study recruitment: High risk**  Neurosurgeons have selected out patients with “trivial” injuries - makes this a higher-risk group than population of interest  **Attrition: Low risk**  Only inpatient measures  **Prognostic factor measurement: Low risk**  Loose definition for abnormal neurology  **Outcome measures: Mod risk**  Only inpatient-related outcome measures  **Confounding factors: Low risk**  None obvious  **Statistical techniques: Mod risk**  Selective reporting of outcomes in regression model  Article concludes all patients should have a repeat CT as 7/360 patients had neurosurgery based solely on repeat CT head findings.  Possibly include but is a higher-risk population given selection out of patients with “non-significant” findings.  Note also 11% of 360 repeat CTs recalled - i.e., initial finding not present (4/6 h after injury). |
| Quigley et al.  2012  Pennsylvania  USA | Pennsylvania  Level 1 trauma center  2004-2011  All patients admitted ICU for at least overnight observation  Inclusion criteria:   - Present on trauma registry - Initial GCS13-15 - Isolated subarachnoid hemorrhage - Does not state adult only but mean age 65.7 years | Retrospective cohort study  Aim:  To assess if traumatic subarachnoid hemorrhage more benign form of mTBI  Multi-variable analysis computed with step-down logistic regression - discharge home primary outcome | Discharge home  Clinical deterioration  CT progression  Neurosurgery | Demographics  Mechanism of injury  Number and results of follow-up CT  Length of hospital and ICU admission  ISS  CTs re-reviewed by study radiologist | 547 patients identified as subarachnoid  478/547 isolated subarachnoid  470/478 repeat CT imaging  15/470 worse CT (1 is new stroke)  342/478 discharged home  51/478 discharged rehab or nursing home  4/478 self discharge  4/479 long-term care facility  1/479 other facility  1/479 to hospice  6 week follow-up 1/478 bilsteral subdural - drained  States surgical intervention 0.2%  Step-down multi-variate regression with outcome discharge home  Age, *p* < 0.0001  Admission GCS, *p* = 0.0018  ISS, *p* = 0.0088  No progression of bleed on CT | **Study recruitment: Low risk**  Identified from prospective trauma registry - dependent on how accurate this is  **Attrition: Mod risk**  Not clear whether and when all patients followed up, but presents outcomes from outpatient clinic  **Prognostic factor measurement: Low risk**  Ct scans reviewed  **Outcome measures: Mod risk**  Not clear if uniform outpatient follow-up  **Confounding factors: High risk**  Clearly an old patient population - discharge to rehab/nursing home like related comorbidities or other injuries  **Statistical techniques: High risk**  Selective reporting of outcomes in regression model  No confidence intervals or odds ratios  No explanation of how the model was derived  **General comments:**  Discharge outcomes contradict low level of intervention.  Unable to pool risk factors as are  Can pool to confirm subarachnoids are low risk |
| Velmahos et al.  2006  Massachusetts  USA | Level 1 trauma center  Massachusetts  USA  2003-2004  All patients with intra-cranial injuries identified reviewed by a neurosurgeon and repeat CT scheduled within 24 h.  Inclusion criteria:   - Present on trauma registry - Initial GCS13-15 - Blunt head injury - Repeat CT for intra-cranial injury - Presumably adults, age presented as mean 48 and SD 25 | Retrospective cohort study  Comparison uni-variate characteristic patients with worse CT scans compared with the same or improved  Where *p* value ≤0.2 included in stepwise logistic regression model | Surgical or medical intervention following repeat CT (caniotomy, ICP monitoring, intubation or mannitol, increased ventilation, CSF drain, sedation, transfer to ICU)  Worse repeat CT | Demographics  ISS  Admission observations  Time interval between admission and 1st CT and subsequent CT scans | 692 patients had CT for head injury  179/692 for scheduled repeat CT  154/692 repeat CT due to intra-cranial injury  25 no lesion - repeat CT due to anti-coagulation  37/154 worse CT  7/154 medical or surgical intervention due to deterioration  4/154 neursourgical  8/179 deaths  1/44 subdurals neurosurg  0/33 SAH neurosurg  1/13 intra-parenchymal neurosurg  0/7 extra-durals  2/57 multiple neurosurgical  Male, *p* = 0.44  Age (years), *p* 0.01  ≤65, *p* < 0.01  Mechanism of blunt trauma, *p* = 0.31  Fall  Road traffic accident  Other 0.31  Injury Severity Score, *p* = 0.01  ISS >16, 0.09  Glasgow Coma Scale score on arrival, *p* = 0.02  Systolic blood pressure on arrival (mm Hg), *p* = 0.63  Anti-coagulation therapy, *p* = 0.25  Time from arrival to CT, *p* < 0.01  First head CT findings solitary or multiple findings, *p* < 0.01  Time between first and second CT, *p* = 0.10  Stepwise logistic regression model to predict worse CT  Time from injury to CT <90 min, OR 6.37, 95% CI 2.29-17.76, *p* < 0.1  Age>65, OR 3.33, 95% CI 1.29-8.60, *p* = 0.01  GCS<15, OR 3.13, 95% 1.23-8.01, *p* = 0.02  Multiple lesions, OR 11.03, 95% CI 1.32-92.06, *p* = 0.03  AUC ROC curve 0.83  If all 4 factors present 83% chance worse CT  If none present 2% chance worse CT  Mean/median GCS = 14.7  Mean/median age = 51  Percent anti-coagulated = 10 | **Study recruitment: Low risk**  Identified from trauma registry - dependent on how accurate this is  Standard model of care for all patients  **Attrition: Low risk**  Appears only inpatient outcomes  **Prognostic factor measurement: Mod risk**  Assessment of time to CT - not clear biological mechanism, how this affects outcome or how measured  **Outcome measures: Mod risk**  Takes reports from attending at face value.  Does not report deaths as a primary outcome but included in table - not clear what the cause of deaths is.  **Confounding factors: High risk**  Not isolated head trauma and no selection out of comorbid patients - does not appear deaths related to head injury but clear  **Statistical techniques: Mod risk**  Selective reporting of outcomes in regression model  **General comments**  Time to initial CT highly significant - slightly odd for this study population - not examined any other study.  No explanation for deaths given in article. |
| Fabbri et al.  2013  Italy | Multi-center  32 Italian hospitals -both specialist and general  2009  Inclusion criteria:   - Any GCS - 18+ years - Head abbreviated AIS ≥1 - No indication for neurosurgery within 7 days - Marshall category 2-4 - Within 24 h of injury   Excluded:   - Need immediate neurosurgery - GCS 3, fixed dilated pupils - Unclear history of mechanism - Hypotension <90 systolic - Penetrating Injuries - Discharge against medical advice | Retrospective multicenter cohort study  Aim:  To assess whether pre-injury anti-platelet use lead to worse outcome in patients with intra-cranial injuries detected by CT imaging | Worse repeat CT defined as increase point on Marshall criteria within 24 h  Neurosurgery within 7 days  GOS at 6 months | Age  Sex  Mechanism  Coagulation  GCS  Anti-platelet medications  Type of injury on CT  Marshall classification | Study of all GCS patients but present data for GCS14-15:  1123/1558 patients GCS14-15  Anti-platelet therapy increased the risk of a worse CT:  When ≤2 lesions  RR 1.86, 95% CI 1.06-3.30, *p* = 0.032  When 3+ lesions  RR 3.34, 95% CI 1.74-6.40, *p* = 0.003  87/1123  Worse characteristic on CT  Mean/median age = 65 | **Study recruitment: Mod risk**  The article is not clear about how patients were identified and data extracted  Also patients requiring emergency surgery within 7 days based on initial CT excluded - may select out higher-risk groups - in practice excluded Marshall 5/6 patients, which is reasonable  **Attrition: Low risk**  No loss to follow-up and standard care for all patients to be reviewed at 6 months  **Prognostic factor measurement: Low risk**  Scans all re-reported  **Outcome measures: Low risk**  Good outcome end points  **Confounding factors: Mod risk**  Not isolated head trauma and state no need to control for comorbidities as shown not to affect head injury outcome  **Statistical techniques: Low risk**  Appropriate and well presented  **General comments:**  Good study  Fabbri previously shared data - ?request GCS13-15 subset |
| Shih et al.  Taiwan  2016 | Tertiary referral Teaching hospital  Taiwan  No time frame given  Inclusion criteria:   - Acute TBI and intra-cranial hemorrhage (epidural, subdural, intra-cerebral, or SAH) - Adult - age range 15-75 years in study   Excluded:   - Penetrating injury - GCS <13 - Immediate neurosurgery - Chronic bleed   All patients reviewed by neurosurgeon who determined whether for immediate neurosurgery or conservative management | Retrospective cohort study  Aim:  Determine the potential risk factors of delayed neurosurgical intervention in mTBI with intra-cranial hemorrhage  Stepwise logistic regression to identify variables that predicted failure of conservative treatment | Neurological deterioration - GCS drop 2+ points, seizures, signs raised ICP  Repeat CT if deterioration - whether worse  Neurosurgical intervention - including craniotomy, craniectomy | Sex  Age  Mechanism of injury  GCS  ISS  Laboratory results including clotting  CT results as reviewed by investigator | 340 patients met inclusion criteria  13/340 neurosurgical outcomes  25/340 neurological decline  7/118 mixed lesions neurosurgery  34/340 worse CT  3/340 died  Univariate analysis: delayed neurosurgery versus non-neurosurgery  Median age, *p* = 0.082  Male/female, *p* = 0.573, OR 0.648, 95% CI 0.196-2.149  GCS, *p* = 0.189  Anti-platelet and/or warfarin therapy, *p* = 0.403, OR 2.188, 95% CI 0.263-18.222  Statin therapy, *p* = 1.000  Hypotension, 0 4, *p* = 1.000  WBC count (1000/mL) *p* = 0.023  RBC count (1000/mL) *p* = 0.401  Hemoglobin, *p* = 0.606  Coagulopathy, *p* = 1.000  Hypertension, *p* = 0.526, OR 0.484, 95% CI 0.105-2.228  Diabetes mellitus, *p* = 1.000, OR 1.028, 95% CI 0.221-4.780 (!?)0  Old cerebral vascular accident = 1.000  Coronary artery diseases, *p* = 1.000  Arrhythmia, *p* = 1.000  Liver cirrhosis, *p* = 1.000  Chronic renal disease, *p* = 1.000  Renal failure, *p* = 1.000  ISS score, median, *p* = 0.005  Single intra-cranial hemorrhage, *p* = 0.149  Multiple intra-cranial hemorrhage, *p* = 0.149  EDH, *p* ≤ 0.001, OR 9.923, 95% CI 3.105-31.708  SDH, *p* = 1.000, OR 0.906, 95% CI 0.298-2.753  IPH, *p* = 0.366, OR 1.812, 95% CI 0.594-5.526  SAH, *p* = 0.044, OR 0.251, 95% CI 0.068-929  IVH, *p* = 0.111 OR, 13.542, 95% CI 1.147-159.876  Midline shift, *p* ≤ 0.001, OR 19.813, 95% CI 5.495-71.435  Skull fracture, *p* ≤ 0.001, OR 21.750, 95% CI 4.707-100.510  Pneumocranium, *p* = 0.621  Volume of EDH, *p* ≤ 0.001  Volume of SDH, *p* = 0.092  Volume of IPH, *p* = 0.657  Stepwise logistic regression: model included WBC count, midline shift, skull fracture, large volume EDH, and higher ISS - significant predictors of delayed neurosurgery.  Volume of extra-dural hemorrhage associated with delayed neurosurgery.  Increase volume EDH 1 cubic cm increased risk of neurosurgery by 16% (*p* = 0.022, OR 1.190, 95% CI 1.041-1.362)  AUC volume EDH = 0.917 (95% CI 0.797-1.00)  Mean/median GCS = 14.7  Mean/median age = 50 | **Study recruitment: Low risk**  No uniform criteria for which patients undergo immediate neurosurgery - just selected by neurosurgeon  **Attrition: Low risk**  Only inpatient measure  **Prognostic factor measurement: Low risk**  Scans all re-reported  **Outcome measures: Mod risk**  Only inpatient measures - potential for discharge and deterioration  **Confounding factors: Mod risk**  Not isolated head trauma  **Statistical techniques: Mod risk**  Mod risk selective reporting of significant prognostic factors. Does not report whole model.  Also some apparent mistakes in uni-variate analysis  **General comments**  Does not report outcomes by single lesion type |
| Bardes et al.  2016  USA | Level 1 trauma center  West Virginia  USA  2009-2011  All mTBI patients with bleeds admitted to general surgical ICU with a neurosurgical consultation  Inclusion criteria:   - Blunt TBI - Age >18 years - GCS 13-15 - ISS <25   Excluded:   - Penetrating injury - GCS <13   States in results all patients had evidence of intra-cranial hemorrhage on bleed - doesn’t define what this includes | Retrospective cohort study  Aim:  Identify low-risk mTBI patients with intra-cranial bleeds who do not require admission to ICU | Documented neurological decline  Medical intervention  Neurosurgical intervention | Admissions GCS  GCS 6, 12, and 24 h  Type of bleed  Bleed progression on CT  Aspirin  Clopidogrel  Warfarin  Admission Coag  ISS | 389 patients met inclusion criteria  5.1% (20) in hospital mortality  53/389 patients neurological decline  376/389 scheduled repeat CT  69/376 worse CT  35/389 craniotomy  46/389 patients required medical or neurosurgical intervention  Uni-variate comparison patients with decline vs. no neurological decline  GCS <15, *p* = 0.002  SDH ,*p* = 0.0025  Age ≥55, *p* = 0.001  Use warfarin, *p* = 0.039  ISS, *p* = 0.22  AIS, *p* = 0.12  SAH, *p* = 0.15  EDH, *p* = 0.18  ICB, *p* = 0.051  Aspirin, *p* = 0.54  Clopidogrel, *p* = 0.17  PT, *p* = 0.042  aPPT, *p* = 0.0028  Admision INR, *p* = 0.42  Decision tree subgroup analysis:  No GCS15 patient ≤55 underwent neurological decline = low-risk group  Mean/median GCS = 14.8  Mean/median age = 63  Percent anti-coagulated = 12 | **Study recruitment: Low risk**  Representative sample of population of interest  Limitations of retrospective data collection  **Attrition: Low risk**  Only inpatient measure  **Prognostic factor measurement: Low risk**  Scans not re-reported  **Outcome measures: Mod risk**  Only inpatient measures - potential for discharge and deterioration  **Confounding factors: Mod risk**  Not isolated head trauma or control for comorbidities  Does use ISS to exclude severe polytrauma  **Statistical techniques: Mod risk**  Mod risk selective reporting of significant prognostic factors.  Does not present decision tree analysis transparently |
| Sharifuddin et al.  2012  Malaysia | Patients admitted under neurosurgeons 2008-2009, specialist center  Inclusion criteria:   - GCS13-15 - ≥12 years - Positive initial head CT - Isolated blunt head injury - Presented within 24 h of initial injury   Excluded:   - Previous history of head injury - On anti-coagulation therapy (aspirin, heparin, or warfarin) - Polytrauma - Major comorbidity - Suspected drug or alcohol intoxication - Neurological impairment trauma - Immediate neurosurgery - Admitted ICU for close observation | Prospective observational study  Aim:  To evaluate whether the repeat head CT is useful in providing information that leads to any neurosurgical intervention | Repeat CT at 24-48 h as categorized:  Unchanged (no change could be assessed based on the size of the injury)  Improving (resolution or improvement based on the size of the injury)  Worsened (increase in size or evidence of new intra-cranial lesion).  Surgical interventions: craniotomy, intracranial pressure monitor placement or intubation. | Sex  Age ≥65 years  Ethnic groups  Mechanism of injury: MVA/Fall/Other  Admission GCS  Associated symptoms  Post-traumatic amnesia  Headache  Vomiting  Dizziness  Type of injury identified | 279 patients met the inclusion criteria  Neurological decline 66 patients (23.7%)  Worse CT in 58 patients (20.8%).  31 (11.1%) patients neurosurgical outcome.  3 deaths  Uni-variate comparison patients with progression on CT and without:  Male. *p* = 0.189  Age ≥65, *p* < 0.001  Ethnic groups, *p* = 0.624  Mechanism of injury  MVA vs. others, *p* = 0.333  GCS <15, *p* = 0.003  Post-traumatic amnesia, *p* = 0.069  Headache, *p* = 0.019  Vomiting, *p* = 0.441  Dizziness, *p* = 0.262  Multiple lesion, *p* = 0.001  Base of skull fracture, *p* = 0.865  Convexity fracture, *p* = 0.842  Hb (g/litre) on admission, *p* 0.009  INR on admission, *p* = 3 0.388  Stepwise multiple logistic regression model  Risk factors for progression on CT:  Age ≥65, *p* < 0.001, 95% CI (0.098-0.364)  Multiple lesions on initial CT, *p* = 0.018, 95% CI (0.239-0.877)  GCS score <15,   *p* = 0.016, 95% CI (1.164-4.333)  44/144 multiple lesion worse CT  Mean/median GCS = 14.6  Mean/median age = 39  Percent anti-coagulated = 0 | **Study recruitment: Low risk**  Retrospective case note review - depends on accuracy of notes.  Not clear if all patients with ICH admitted under neurosurgeon - potential for selection of high-risk population. Note age 12+ does not strictly meet inclusion criteria.  **Attrition: Low risk**  Outcomes only during hospital admission - no loss to follow-up  **Prognostic factor measurement: Mod risk**  The mechanism of injury - doesn’t discriminate between high- and low-risk mechanisms.  CT interpreted once by attending radiologist or neurosurgeon. No quality control.  **Outcome measures: Low risk**  As reported outcomes of worse CT, neurosurgery, or death as an inpatient low risk for bias. However, no follow-up outcome measures for delayed deterioration.  **Confounding factors: Mod risk**  Possibility of anti-coagulants. Not recorded.  **Statistical techniques: Mod risk**  Stats do not present what the risk measure is - presumably an OR. Also selective reporting of significant results.  Only for progression on CT - dubious value |
| Sumritpradit et al.  2016  Bangkok  Thailand | Patients admitted to an acute care unit surgery 2009-2013  Inclusion criteria:   - Admission <72 h - Age ≥16 years - Positive initial head CT - Non-surgical initial management - Includes all GCS scores but presents data for GCS13-15 patients - Patients underwent repeat CT imaging - determined after neurosurgical review | Retrospective cohort study  Aim:  To determine the value of repeat CT imaging in TBI for risk stratification of patients | Neurological deterioration: reduced consciousness, limb weakness, lateralizing signs, severe headache, vomiting, and dizziness  Neurosurgery | Age  Sex  Comorbidities  Medications  Initial GCS  AIS  Medications  CT findings | 145 patients matched inclusion criteria.  98/145 GCS13-15  74/98 routine repeated CT scans  (36/98 worse)  (1/74 neurosurgical)  24/98 clinically deteriorated and underwent CT imaging (7/28 neurosurgery)  Overall  8/98 GCS13-15 patients neurosurgery  24/98 some clinical deterioration - prompting repeat CT  GCS13-15  Uni-variate comparison patients underwent neurosurgery and did not  Age >50, *p* = 0.478  Mean age, *p* = 0.295  Male, *p* = 0.706  Traffic injury, *p* = 0.256  Diabetes mellitus, *p* = 0.354  Hypertension, *p* = 0.135  Ischemic heart disease, *p* = 0.070  Cerebrovascular disease, *p* = 0.592  Aspirin, *p* = 1.000  Warfarin, *p* = 1.000  Clopidogrel, *p* = 0.017  ISS, mean, *p* = 0.405  ISS >19, *p* = 0.282  Brain AIS, mean, *p* = 0.080  AIS >4, *p* = 0.073  SBP, *p* = 0.240  Heart rate on admission, mean, *p* = 0.095  Epidural hematoma, *p* = 1.000  Subdural hematoma, *p* = 0.136  Subarachnoid hemorrhage, *p* = 0.464  Hemorrhagic contusion, *p* = 0.715  Intraventricular hemorrhage, *p* = 1.000  Diffuse axonal injury, *p* = 1.000  Skull fracture, *p* = 1.000  Base of skull fracture, *p* = 0.409  Midline shift >2 mm, *p* = 0.003  Duration from injury to 1st CT, *p* = 0.603  Odds ratios associated with these factors reported separately:  Subdural hematoma, OR 5.3, 95%CI (0.63-45.33), *p* = 0.136  Hypertension,  OR 4.1, 95% CI (0.78-21.46), *p* = 0.135  AIS >4, OR 4.0, 95%CI (0.91-17.55), *p* = 0.073  Ischemic heart disease, OR 4.8, 95% CI (0.99-23.19), *p* = 0.070  Clopidogrel, OR 10.2, 95 CI (1.87-55.38, *p* = 0.017  Midline shift >2 mm, OR 11.9, 95% CI (2.50-57.20), *p* = 0.003  Neurological deterioration resulting in CT, OR 30.0, 95% CI (3.46-280.83), *p* < 0.001  Mean/median age = 57  Percent anti-coagulated = 4 | **Study recruitment: High risk**  Only recruited patients for whom neurosurgeons had planned a repeat CT scan (293/442 patients with injuries no repeat CT vs. 149/442 for repeat CT)  Selection bias of higher-risk group then all GCS13-15 patients with CT-detected injuries  **Attrition: Low risk**  Outcomes only during hospital admission - no loss to follow-up  **Prognostic factor measurement: Mod risk**  No outline of how CT scans reported and risk stratified  **Outcome measures: Low risk**  As reported outcomes of worse CT, neurosurgery, or death as an inpatient low risk for bias. However, no follow-up outcome measures for delayed deterioration.  **Confounding factors: Mod risk**  Does not state how patient with other injuries dealt with  **Statistical techniques: Low risk**  Presents simple uni-variate analysis between neurosurgical and non-neurosurgical patients  Is a higher-risk population due to selection for repeat CT imaging - possibly unable to include in any meta-analysis. |
| Sifri et al.  2006  New Jersey  USA | Level 1 trauma centre  New Jersey  USA  2002-2003 12 months  Inclusion criteria:   - Initial GCS13-15 - Intra-cranial bleed - intra-cerebral, extra-dural, subdural subarachnoid, or contusion   Excluded:   - Previous brain surgery or cerebral pathology or chronic neurological condition like dementia - Concurrent spinal injury - Anti-coagulated or existing clotting disorder - Patients who underwent immediate or planned neurosurgery due to first CT   Patients who only underwent 1 CT | Prospective cohort study  Aim:  Prospectively assess the value of a repeat CT in patients with mTBI and intra-cranial hemorrhage and normal neurological examination  Repeat CT within 24 h | Neurosurgery following second scan  Admission to ICU or administration of mannitol following second scan  In hospital mortality  GOS at discharge  Discharge destination | Abnormal neurological examination prior to repeat CT (GCS<15 or severe headache/vomiting/gross motor or sensory deficits)  Sex  Age  GCS  Mechanism  Type of injury identified by CT | 161 patients GCS13-15 with intra-cranial bleed  10 excluded due to comorbidities.  5 required immediate neurosurgery  16 did not undergo repeat imaging  130 in study population  99 normal neurology at time of repeat CT; 31 abnormal neurology at time of repeat CT  0/99 neurosurgery  1/99 death (unrelated to intra-cranial injury)  13% 99 CT scans worse  2/31 neurosurgery  5/31 deaths  14/31 repeat CTs worse  Abnormal neurological exam predicts changes repeat CT, OR 5.28, CI 2.08-13.4, *p* = 0.002  Mean/median GCS = 14.6  Mean/median age = 45  Percent anti-coagulated = 0 | **Study recruitment: Mod risk**  Only patients with repeat CT - likely to be a higher-risk group  **Attrition: Low risk**  Only inpatient measures  **Prognostic factor measurement: Mod risk**  Does not try and grade severity of CT findings as predictor  Loose definition for abnormal neurology - sometimes prompted repeat CT and no uniformed time when all CT scans performed.  **Outcome measures: Mod risk**  Only inpatient-related outcome measures.  **Confounding factors: Mod risk**  Cohort includes patients with multiple injuries and abnormal observations.  **Statistical techniques: Low risk**  Minimal statistical analysis |
| Bee et al.  2009  Tennessee  USA | Level 1 trauma center  USA  2005-2007  Identified from trauma registry  All patients admitted to ICU under neurosurgeon and received a repeat CT scan  Inclusion criteria:   - mTBI - Blunt trauma to head - GCS14-15 - Intra-cranial injury CT head   Excluded:   - Facial or skull fractures - Immediate neurosurgery - Other injuries requiring ICU admission   Data only presented for adults (15-94 years) | Retrospective cohort study  Aim:  Assess whether repeat CT imaging and ICU admission necessary in mTBI with intra-cranial injury | Worse CT  Clinical examination change  Neurosurgical intervention | Age  Sex  Admission observations  AIS  ISS  Admission GCS | 207 patients met inclusion criteria  58/207 worse CT or neurology requiring intervention (4 neurology only)  31/77 patients multiple/mixed lesions worse CT  18/207 neurosurgery  2 deaths (1 due to stoke, other following craniotomy)  5/18 neurosurgical = subdurals with no clinical change but worse CT  Uni-variate comparison: worsening CT or worsening neurology requiring an intervention vs. no deterioration (58 vs. 149)  Average age worse 47 (47.2 ± 19.8). No worse 45 (45.5 ± 18.7), *p* = 0.56  Average admission SBP worse 152 (152.3 ± 28.3). No worse 143 (143.1± 25.9), *p* = 0.03  Average admission pulse worse 87 (86.9 ± 15.3). No worse 88 (88.5± 16.1), *p* = 0.556  Average HAIS worse 4.2 (4.21 ± 0.55). No worse 3.8 (3.84 ± 0.54), *p* < 0.0001  Average ISS worse 22.3 (22.3 ± 6.25). No worse 19.6 (19.6 ± 6.9), *p* = 0.018  Mean/median age = 46 | **Study recruitment: Low risk**  Dependent on accuracy of trauma registry  **Attrition: Low risk**  Low risk - inpatient outcomes  **Prognostic factor measurement: Medium risk**  No re-reporting of CTS  **Outcome measures: Medium risk**  No outcome measures after discharge  **Confounding factors: Medium risk**  No control for comorbidities  **Statistical techniques: Low risk**  Higher rates of adverse outcome than other studies |
| Darby MSc Thesis  2015  USA | Level 1 trauma center  California  USA  2007-2011  Patients identified on a hospital trauma registry  Inclusion criteria:   - Initial GCS13-15 - Blunt head trauma - Positive CT scan - 2 or more CT scans - Age 18+ years   Excluded:   - Pregnant - Age <18 years - Penetrating injury | Retrospective cohort study  To assess whether GCS15 patients with intra-cranial hemorrhage who maintain a GCS of 15 benefit from routine CT imaging | Worse repeat CT imaging  Neurosurgical outcomes | Age/Age 65 +  Anti-coagulant medication  ISS  LOC  Skull fracture displaced/undisplaced  Neurological symptoms  Time interval between scans  GCS/deterioration in GCS | 658 patients GCS13-15 with positive CT scans  88 incomplete notes  201 only 1 CT scan  Study population 369 patients with at least 2 CT scans  111/369 GCS15 at presentation and throughout  0/111 neurosurgery  20.7% of 111 worse CT  0.9% mortality  258 GCS <15 at some point during hospital admission  37.6%, 258 worse CT  11/258 neurosurgery  2.7%, 258 deaths  Overall 11/369 neurosurgical interventions  Mean/median age = 53  Progression of injury:  Unstable GCS <15: unadjusted OR 2.21 (95% CI 1.33-3.68), adjusted 1.71 (95 % CI 1.00-2.91), *p* = 0.05  ISS: unadjusted 1.04 (95% CI 1.01-1.07), adjusted 1.1 (0.99-1.05), *p* = 0.27  Age: unadjusted 1.01 (95% CI 1-10.2), adjusted 1.01 (0.99-1.02), *p* = 0.08  Anti-coagulation: unadjusted 1.02 (95% CI 0.59-1.77), adjusted 0.76 (0.40-1.47), *p* = 0.42  Risk for neurosurgery:  Unstable GCS: unadjusted 4.16 (0.51-33.63), adjusted 2.98 (0.35-25.18), *p* = 0.32  ISS: unadjusted 1.04 (1.01-1.07), adjusted 1.05 (0.99-1.12), *p* = 0.10  Age: Unadjusted 1.01 (1.00-1.02), adjusted 1.11 (0.96-1.28) | **Study recruitment: High risk**  Approximately 1/3 of patients with injuries detected by CT imaging not included either because incomplete or only 1 CT scan.  Patients on which multiple scan conducted likely to be higher risk.  **Attrition: Low risk**  Low risk - inpatient outcomes  **Prognostic factor measurement: Medium risk**  No re-reporting of CTS  Does not include CT findings as a prognostic factor.  **Outcome measures: Medium risk**  No outcome measures after discharge  **Confounding factors: Medium risk**  No control for comorbidities  **Statistical techniques: Mod risk**  Performs different analysis for neurosurgical outcomes compared with worsening CT scans. |
| Fabbri et al.  2008  Italy | District general hospital, rural Italy  Prospective recruitment 1999-2006  Inclusion criteria:   - Admission GCS score ≥9 - Age >10 years - Initial head CT scan positive for any type of trauma - Initial non-operative management   Excluded:   - Persistent hypotension caused by additional injuries - Patients requiring immediate surgery - Penetrating injuries - Patients who have been intubated | Prospective cohort study  Aim:  Evaluate the effects on outcome of a model based on observation in a neurosurgical unit vs. observation in a peripheral hospital with neurosurgical expertise via a teleradiology system and an NSU transfer time of 30-60 min | Follow-up GOS at 6 months (includes mortality)  Neurosurgical intervention within 7 days | Age  Coagulation status  Charlson Co-morbidity Index  Injury Severity Score  GCS  CT scan results - Marshall category  Type of Injury | *N* = 718, GCS13-15, patient age >12 years  Anonymized; individual patient made available by authors and used for analysis. |  |

|  | | | | | | |
| --- | --- | --- | --- | --- | --- | --- |
| *Articles deriving and validating the BIG criteria (*n *= 3; not included in meta-analysis)* | | | | | | |
| *Reference* | *Population* | *Study design* | *Outcome measures* | *Prognostic factors assessed* | *Results* | *Quality appraisal* |
| Joseph et al.  2014  USA  Study 1: defining the BIG criteria | Level 1 trauma center  2009-2011  Inclusion criteria:   - All TBI patients with CT findings = skull fracture/ICH   Exclusion criteria:   - Transfer or patients requiring emergent surgical intervention   Categorization of these patients into 3 criteria - derived through local consensus  BIG 1 (discharge after 6 h observation from ED):   - GCS13-15, normal pupils and no focal neurological deficit - Not intoxicated - Not anti-coagulated or anti-platelets - Single ICH <5 mm and no skull fracture single IPH   BIG 2 (admit to hosp. not neurosurgeon)   - GCS13-15, normal pupils and no focal neurological deficit - Can be intoxicated - Non-displaced skull fracture - Bleed 5-7 mm - 2 intra-cerebral bleeds 3-7 mm - Not anti-coagulated or antiplatelets   BIG 3 (repeat CT and admit under neurosurgeon HDU)   - GCS<13 or abnormal pupils or focal neurological deficit - Taking anti-coagulant or anti-platelets - Multiple types of injury on CT - Bleeds >7 mm - Displaced skull fractures - Intubated patients | Retrospective cohort study  Aim:  Define guidelines for base patients’ history, examination, and initial CT head findings regarding which patients require observation in ED, RHCT, or neurosurgical consultation.  Local consensus for categories | Neurosurgical intervention  Progression of CT findings on a repeated scan  Neurological deterioration if BIG 1 or 2 - GCS <12, abnormal focal neurology, or abnormal pupils | Anti-coagulation  Anti-platelets  OBS on admission to ED  GCS  Intoxication  CT head scans all reviewed by a single investigator to give size of bleed and associated findings. | 1232 patients TBI with positive CT scan  121 = BIG 1  313 = BIG 2  798 = BIG 3  888/1232 underwent repeat CT  13% (159) patients neurosurgical outcome - all in BIG 3 category  No BIG 1 patients had neurological deterioration.  No Big 1 patients worsening CT  2.6% (9) BIG 2 patients worsening CT  2/313 BIG 2 patients deteriorated neurologically - transferred to neurosurgical care.  No BIG 2 patient needed neurosurgery.  BIG 3 patients:  21.6% worsening CT  3% neurosurgical intervention | **Study recruitment: Low risk bias**  Retrospective cohort review - reliant on accuracy of written notes  Cohort identified by case note review but no details of how this was done - possible selection bias. What constitutes emergent surgical intervention - how many from BIG 1/BIG 2 criteria excluded by this?  **Attrition: Low risk**  Inpatient outcomes only  **Prognostic factor measurement: Mod risk**  Radiology report double-checked by one person only. Definition of neurological deterioration is defined differently as altered mental state and focal deficit and GCS <13 in different places.  **Outcome measures: Mod risk**  No routine follow-up of all patients - must re-attend at same hospital to register  **Confounding Factors: Low risk**  Age affects outcome and size of bleed.  **Statistical techniques: N/A** |
| Joseph et al.  2014  USA  Study 2 validating the BIG criteria  Identified Search Strategy | Level 1 Trauma centre  USA  March 2012-Dec 2013  Inclusion criteria BIG 1 patients:   - GCS13-15, normal pupils, and no focal neurological deficit - Not intoxicated - No anti-coagulated or anti-platelets - Single ICH <5 mm and no skull fracture - Single IPH   Excluded:   - Patients transferred from other hospital - Intubated - Patients undergoing emergent neurosurgical intervention - Unexaminable patients | Prospective cohort study  Aim:  To evaluate the established BIG 1 category for managing patients with traumatic brain injury | Patients remained in ED for observation for 6 h. If no neurological deterioration - discharged.  Repeated neurological assessment every 2 h - if GCS <13, unequal pupils, or focal neurological deficit - neurological deterioration  Need for neurosurgical intervention  Need for repeat CT due to neurological deterioration  Hospital or ICU admission  In-hospital mortality  30-day re-admission | Prospectively recorded:  Age  Sex  Admission observations  Neurological assessment of GCS, examination, and pupils  Intoxication  Anti-platelet or anti-coagulation  Intubation  LOC  Initial CT findings by attending radiologist - confirmed by study radiologist | States 148 patients met criteria prospectively.  127/148 patients included and matched 127 patients with matched characteristics of demographics, medications, and CT findings before implementation of BIG criteria.  No patients underwent neurosurgery, had neurological deterioration, or died, both of the 127 prospectively recruited and those matched retrospectively.  Statistically significant reduction in hospital admissions, ICU admissions, and repeat CT imaging in prospective cohort post implementation of BIG criteria.  0 30-day re-admissions although 5 ED visits | **Study Recruitment: Mod risk**  States GCS13-15 and range presented as GCS13-15 but also excludes unexaminable patients and patients with altered mental state - appears cohort does not contain all GCS14 and 13 patients. Not clear about how the cohort was prospectively recruited.  **Attrition: Mod risk**  Disregards 21 of recruited cohort in analysis to match with retrospectively available patients.  **Prognostic factor measurement: Mod risk**  Reliability of case notes - may be incomplete  The definitions of bleed size are subjective.  Abnormal focal neurology is subjective and clinician dependent. CT scan re-reviewed by a single researcher - possible bias.  **Outcome measures: Mod risk**  Measures: no structured follow-up of every patient. Patients could have been discharged and died in the community - study would have missed this. States over 50% admitted but that all discharged from the ED in the abstract.  **Confounding factors: Mod risk**  Age not part of BIG 1 but could affect outcome and size of bleed  **Statistical techniques: N/A**  **General Points**  Small numbers of patients in this specific setup. Would support small CT findings low risk, but risk stratification very dependent on accuracy and consistency of radiology report. |
| Joseph et al.  2015  USA  Study 2: further validation of BIG criteria | Level 1 trauma center  Pre BIG TBI March 2011-Feb 2012  Post BIG July 2012-June 2013  Inclusion criteria:   - All patients with blunt trauma mechanism and ICH/skull fracture   Excluded:   - Transfers - Dead on arrival - Needed immediate neurosurgery   Presents subgroup analysis of BIG 1 patients  Inclusion criteria BIG 1 patients:   - GCS13-15, normal pupils, and no focal neurological deficit - Not intoxicated - No anti-coagulated or anti-platelets - Single ICH <5 mm and no skull fracture - Single IPH | Prospective cohort study  Compare outcomes in TBI before and after implementation of BIG criteria | Number of routine repeat CT head scans  Neurosurgical consultations  Progression of bleed on CT  Neurosurgical intervention during hospital admission (craniotomy, craniectomy, ICP monitoring)  ICU admission  30-day re-admission | Prospectively recorded:  Age  Sex  Admission observations  Neurological assessment of GCS, examination, and pupils.  Intoxication  Anti-platelet or anti-coagulation  Intubation  LOC  Initial CT findings by attending radiologist - confirmed by study radiologist | Pre BIG  87 BIG 1/415  0 neurosurgery  0 deaths  3 progression on CT  68 (78%) admitted  24 (27.5%) admitted ICU  76 (87.4%) neurosurg consultations  59 (67.8%) repeat CT  Post Big  83 BIG 1/381  0 neurosurgery  0 deaths  1 progression on CT  42 admitted (50.6%)  6 ICU admission (7.2%)  7 (8.4%) neurosurg consultation  6 (7.2%) repeat CT  Statistically significant (*p* < 0.001 admission hospital, ICU, repeat CT imaging, and neurosurgical consultation post introduction of BIG criteria) | **Study recruitment: Low risk**  States all patients with TBI prospectively recorded on data - not clear how patients identified and recruited.  Emergent neurosurgical patients excluded - no definition given.  **Attrition: Low risk**  Outcomes only as inpatients or if re-present  **Prognostic factor measurement: Mod risk**  CTs are reviewed by a member of study group - the cut offs are slightly subjective on CT measurement.  **Outcome measures: Mod risk**  Only measures as inpatient/re-presentation. Potential for discharge and deterioration.  **Confounding factors: Low risk**  Age  **Statistical techniques: Mod risk**  Presents data for all patients or BIG 1 patients - not all GCS13-15 patients |

AIS, _______; aPPT, activated partial thromboplastin time; AUC, area under the curve; BP, blood pressure; CCHR, Canadian CT head Rule; CI, confidence interval; CSF, cerebrospinal fluid; CT, computed tomography; CT-VE, _____________; CVA, cerebrovascular accident; CTS, ______; DOACs, direct oral anti-coagulants; DNACPR, do not attemp cardiopulmonary resuscitation; ED, emergency department; EDH, extra-dural hemorrhage; FBC, full blood count; FFP, fresh frozen plasma; GCS, Glasgow Coma Scale; GOS-E, Extended Glasgow Outcome Scale; HDU, high dependency unit; HR, heart rate; HTN, hypertension; ICB, intra-cranial bleed; ICD, International Classification of Diseases; ICH, intra-cerebral hemorrhage; ICP, intra-cranial pressure; ICU, intensive care unit; INR, international normalized ratio; IPH, intra-parenchymal hemorrhage; ISS, Injury Severity Score; ITP, idiopathic thrombocytopenic purpura; LMWH, low molecular weight heparin; LOC, loss of consciousness; LOS, length of stay; MHI, _________; MTBI, mild traumatic brain injury; MVA, motor vehicle accident; NSW, ______; OBS, _______; PT, ______; PTT, partial thromboplastin time; RBC, red blood cell; RCT, random controlled trial; RHGT, ______; ROC, receiver operator characteristic; RR, relative risk; SBP, ; SDH, subdural hematoma; TBI, traumatic brain injury; WBC, white blood cell.

Supplementary Table 3. Full Studies Retrieved and Excluded

| *No.* | *Study* | *Reason excluded* |
| --- | --- | --- |
| 1. | Anonymous et al.^31^  (Full study revealed duplicate of Corrigendum et al.^146^) | Unable to differentiate initial GCS13-15 patients |
| 2. | Bajsarowicz et al.^34^ | Abstract only |
| 3. | Bajsarowicz et al.^33^ | Unable to differentiate initial GCS13-15 patients |
| 4. | Baldawa et al.^35^ | Letter about included study |
| 5. | Basahm et al.^36^ | Unable to differentiate initial GCS13-15 patients |
| 6. | Carlson et al.^38^ | Included pediatric patients and patients with no injuries identified by CT imaging |
| 7. | Chen et al.^39^ | Uses lumbar puncture to diagnose brain injury |
| 8. | Choudhry et al.^41^ | Duplicate study^40^ |
| 9. | Flaherty et al.^43^ | Abstract only |
| 10. | Gore et al.^44^ | Abstract only |
| 11. | Iaccarino et al.^45^ | Unable to differentiate initial GCS13-15 patients |
| 12. | Inamasu et al.^46^ | Unable to differentiate initial GCS13-15 patients |
| 13. | Jacobs et al.^47^ | Includes patients no injuries on CT imaging |
| 14. | Jiang et al.^48^ | Included patients of initial GCS <13  Not clear if all GCS13-15 patients have injuries present on CT imaging |
| 15. | Jiang et al.^49^ | Included patients of initial GCS <13  Not clear if all GCS13-15 patients have injuries present on CT imaging |
| 16. | Joseph et al.^50^ | Unable to differentiate initial GCS13-15 patients |
| 17. | Joseph et al.^51^ | Unable to differentiate initial GCS13-15 patients |
| 18. | Joseph et al.^53^ | Unable to differentiate initial GCS13-15 patients |
| 19. | Kim et al.^56^ | Unable to differentiate initial GCS13-15 patients |
| 20. | Kreitzer et al.^58^ | Abstract only (full study included^86^) |
| 21. | McCutcheon et al.^61^ | Unable to differentiate initial GCS13-15 patients |
| 22. | Nishijima et al.^64^ | Abstract only and associated article included patients of initial GCS <13 |
| 23. | Nishijima et al.^67^ | Unable to differentiate initial GCS13-15 patients |
| 24. | Nishijima et al.^68^ | Unable to differentiate initial GCS13-15 patients |
| 25. | Penn et al.^70^ | Abstract only (full study included^37^) |
| 26. | Rubino et al.^72^ | Outpatient setting |
| 27. | Orringer et al.^79^ | Unable to differentiate initial GCS13-15 patients |
| 28. | Yuan et al.^80^ | Unable to differentiate initial GCS13-15 patients |
| 29. | Zare et al.^81^ | Includes pediatric population |
| 30. | Zhao et al.^82^ | Not clear about inclusion criteria and definition of non-operative - no response from authors when contacted |
| 31. | Park et al.^83^ | Unable to differentiate initial GCS13-15 patients |
| 32. | Schuster et al.^84^ | Unable to differentiate initial GCS13-15 patients |
| 33. | Smith et al.^85^ | Unable to differentiate initial GCS13-15 patients |
| 34. | Choudhry et al^88^ | Abstract only (full study included^40^) |
| 35. | Tong et al.^147^ | Unable to differentiate initial GCS13-15 patients |
| 36. | Yadav et al.^91^ | Unable to differentiate initial GCS13-15 patients and included children |
| 37. | Cohen et al.^92^ | Includes patients with no injury on initial CT |
| 38. | Stein et al.^105^ | Theoretical study - no data |
| 39. | Borovich et al.^110^ | Case reports |
| 40. | Knuckey et al.^111^ | Pre-1996 |
| 41. | Chen et al.^112^ | Pre-1996 |
| 42. | Mertol et al.^113^ | Case reports pre-1996 |
| 43. | Brown et al.^115^ | Unable to differentiate initial GCS13-15 patients |
| 44. | Fainardi et al.^117^ | Unable to differentiate initial GCS13-15 patients |
| 45. | Karasu et al.^118^ | Unable to differentiate initial GCS13-15 patients and includes children |
| 46. | Türedi et al.^120^ | Includes patients with no injury on initial CT |
| 47. | Connon et al.^121^ | Unable to differentiate initial GCS13-15 patients |
| 48. | Chang et al.^148^ | Unable to differentiate initial GCS13-15 patients |
| 49. | Chao et al.^123^ | Unable to differentiate initial GCS13-15 patients |
| 50. | Sullivan et al.^124^ | Unable to differentiate initial GCS13-15 patients |
| 51. | Innocenti et al.^126^ | Includes patients with no injury on initial CT |
| 52. | Muszynski et al.^127^ | Includes children |
| 53. | Patel et al.^128^ | Unable to differentiate initial GCS13-15 patients |
| 54. | Lingsma et al.^129^ | Includes patients with no injury on initial CT |
| 55. | Wong et al.^131^ | Case studies and pre-1996 |
| 56. | Offner et al.^132^ | Unable to differentiate initial GCS13-15 patients |
| 57. | Wong et al.^133^ | Duplicate of no. 55 |
| 58. | Bhau et al.^134^ | Unable to differentiate initial GCS13-15 patients |
| 59. | Chen et al.^39^ | Includes children and patients without CT-identified injuries |
| 60. | Gaetani et al.^135^ | Unable to differentiate initial GCS13-15 patients |
| 61. | Greene et al.^136^ | Unable to differentiate initial GCS13-15 patients |
| 62. | Son et al.^137^ | Unable to differentiate initial GCS13-15 patients |
| 63. | Pradeep et al.^138^ | Unable to differentiate initial GCS13-15 patients |
| 64. | Alahmadi et al.^149^ | Unable to differentiate initial GCS13-15 patients |
| 65. | Chieregato et al.^116^ | Includes children |
| 66. | Kehoe et al.^95^ | Unable to differentiate initial GCS13-15 patients |
| 67. | Lesko et al.^96^ | Unable to differentiate initial GCS13-15 patients |
| 68. | Lawrence et al.^94^ | Includes children |
| 69. | Roka et al. 2008^119^ | Includes children |

CT, computed tomography; GCS, Glasgow Coma Scale.

Supplementary Table 4. Characteristics of Included Studies

| *No.* | *Study* | *Type* | *Size* | *Outcomes* | *Estimate of*  *outcome of interest* | *Uni-variate of analysis of any prognostic factor* | *Multi-variable*  *model of several prognostic factors* |
| --- | --- | --- | --- | --- | --- | --- | --- |
| 1 | Sifri et al. 2006^75^ | Prospective  cohort | 130 | Death  Neurosurgery  Progression CT | ✔ | ✔ |  |
| 2 | Brown et al. 2007^114^ | Prospective cohort | 142 | Death  Deterioration  Neurosurgery  Progression CT | ✔ |  |  |
| 3 | Fabbri et al. 2008^139^ | Prospective cohort | 723 | Death  Neurosurgery | ✔ | ✔ |  |
| 4 | AbdelFattah et al. 2012^28^ | Prospective cohort | 145 | Death  Deterioration  Progression CT | ✔ |  |  |
| 5 | Sharifuddin et al. 2012^74^ | Prospective cohort | 279 | Death  Deterioration  Neurosurgery  Progression CT | ✔ | ✔ | ✔ |
| 6 | Ding et al. 2012^90^ | Prospective trial | 32 | Neurosurgery  Progression CT | ✔ |  |  |
| 7 | Nishijima et al. 2014^66^ | Prospective cohort | 600 | Deterioration  Neurosurgery | ✔ | ✔ | ✔ |
| 8 | Sifri et al. 2004^102^ | Retrospective cohort | 202 | Death  Deterioration  Neurosurgery  Progression CT | ✔ |  |  |
| 9 | Velmahos et al. 2006^77^ | Retrospective cohort | 154 | Deterioration  Neurosurgery  Progression CT | ✔ | ✔ | ✔ |
| 10 | Huynh et al. 2006^97^ | Retrospective  Cohort | 56 | Deterioration  Neurosurgery  Progression CT | ✔ |  |  |
| 11 | Bee et al. 2009^99^ | Retrospective cohort | 207 | Death  Neurosurgery | ✔ | ✔ |  |
| 12 | Klein et al. 2010^57^ | Retrospective cohort | 323 | Death  Neurosurgery | ✔ |  |  |
| 13 | Schaller et al. 2010^8^ | Retrospective Cohort | 110 | Death  Deterioration  Neurosurgery | ✔ |  |  |
| 14 | Nasir et al. 2011^106^ | Retrospective cross-sectional | 275 | Neurosurgery  Progression CT | ✔ |  |  |
| 15 | Sifri et al. 2011^125^ | Retrospective cohort | 107 | Deterioration  Neurosurgery  Progression CT | ✔ |  |  |
| 16 | Levy et al. 2011^59^ | Retrospective cohort  SAH only | 117 | Death  Neurosurgery  Progression CT | ✔ |  |  |
| 17 | Washington et al. 2012^78^ | Retrospective cohort | 321 | Deterioration  Neurosurgery  Progression CT | ✔ | ✔ | ✔ |
| 18 | Homnick et al. 2012^104^ | Retrospective cohort | 341 | Death  Deterioration  Neurosurgery  Progression CT | ✔ |  |  |
| 19 | Nayak et al. 2013^62^ | Retrospective cohort | 321 | Death  Neurosurgery  Progression CT | ✔ |  |  |
| 20 | Borczuk et al. 2013^37^ | Retrospective cohort | 404 | Deterioration  Neurosurgery | ✔ | ✔ | ✔ |
| 21 | Almenawer et al. 2013^18^ | Retrospective cohort study and meta-analysis | 445 | Neurosurgery  Progression CT | ✔ |  |  |
| 22 | Joseph et al. 2013^52^ | Retrospective cohort | 270 | Death  Neurosurgery | ✔ |  |  |
| 23 | Thorston et al. 2012^6^ | Retrospective cohort | 360 | Neurosurgery  Progression CT | ✔ | ✔ | ✔ |
| 24 | Choudhry et al. 2013^41^ | Retrospective cohort | 757 | Death  Deterioration  Progression CT | ✔ | ✔ | ✔ |
| 25 | Deepika et al. 2013^42^ | Retrospective cohort  SAH only | 34 | Unable to extract |  |  |  |
| 26 | Fabbri et al. 2013^87^ | Retrospective cohort | 1123 | Progression CT | ✔ | ✔ |  |
| 27 | Boris et al. 2013^107^ | Retrospective cohort | 68 | Deterioration  Neurosurgery  Progression CT | ✔ |  |  |
| 28 | Thomas et al. 2010^7^ | Retrospective cohort | 457 | Deterioration  Neurosurgery | ✔ |  |  |
| 29 | Nishijima et al. 2013^63^ | Retrospective cohort | 1412 | Deterioration  Neurosurgery | ✔ |  |  |
| 30 | Quigley et al. 2013^71^ | Retrospective cohort  SAH only | 478 | Neurosurgery  Progression CT | ✔ |  | ✔ |
| 31 | Levy et al. 2014^60^ | Retrospective cohort | 76 | Deterioration  Neurosurgery | ✔ |  |  |
| 32 | Overton et al. 2014^69^ | Retrospective cohort | 171 | Deterioration | ✔ |  | ✔ |
| 33 | Phelan et al. 2014^103^ | Retrospective cohort  SAH only | 77 | Death  Deterioration  Neurosurgery  Progression CT | ✔ |  |  |
| 34 | Kreitzer et al. 2014^86^ | Retrospective cohort | 323 | Death  Neurosurgery | ✔ |  |  |
| 35 | Kim et al. 2014^55^ | Retrospective cohort  Subdurals only | 98 | Neurosurgery  Progression CT | ✔ | ✔ | ✔ |
| 36 | Sweeney et al. 2015^98^ | Retrospective cohort | 50,493 | Neurosurgery | ✔ | ✔ | ✔ |
| 37 | Nishijima et al. 2015^65^ | Retrospective cohort | 151 | Deterioration | ✔ |  |  |
| 38 | Darby et al. 2015^130^ | Retrospective cohort | 369 | Death  Neurosurgery  Progression CT | ✔ |  | ✔ |
| 39 | Beynon et al. 2015^93^ | Retrospective cohort | 70 | Death  Neurosurgery | ✔ |  |  |
| 40 | Joseph et al. 2015^54^ | Retrospective cohort | 876 | Neurosurgery  Progression CT | ✔ | ✔ | ✔ |
| 41 | Ditty et al. 2015^32^ | Retrospective cohort  SAH/ICB only | 500 | Death  Neurosurgery  Progression CT | ✔ |  |  |
| 42 | Anandalwar et al. 2016^30^ | Retrospective cohort | 142 | Deterioration  Neurosurgery | ✔ |  |  |
| 43 | Bardes et al. 2016^101^ | Retrospective cohort | 389 | Death  Deterioration  Neurosurgery  Progression CT | ✔ | ✔ | ✔ |
| 44 | Shih et al. 2016^100^ | Retrospective cohort | 340 | Deterioration  Neurosurgery  Progression CT | ✔ | ✔ | ✔ |
| 45 | Schwed et al. 2016^73^ | Retrospective cohort | 201 | Deterioration  Neurosurgery | ✔ | ✔ | ✔ |
| 46 | Sumritpradit et al. 2016^76^ | Retrospective cohort | 98 | Deterioration  Neurosurgery  Progression CT | ✔ | ✔ |  |
| 47 | Pruitt et al. 2016^108^ | Retrospective cohort | 1053 | Deterioration  Neurosurgery | ✔ |  |  |
| 48 | Jospeph et al.^9,27,109^ | Three articles outlining the Brain Injury Guideline risk stratification tool and a combination of retrospective and prospective data following its implementation | | | | | |
| 49 |  |  |  |  |  |  |  |
| 50 |  |  |  |  |  |  |  |

CT, computed tomography; ICB, intra-cranial bleed; SAH, subarachnoid hemorrhage.

Supplementary Table 5. Risk Factors Assessed

| *Risk factor* | | | *Assessed number of studies* | *Uni-variate* | *Multi-variate* | *Recursive partitioning* |
| --- | --- | --- | --- | --- | --- | --- |
| *1. Age* | | *Continuous* | **10**^6,55,69,71,73,76,77,98-100,130^ | **7**^6,55,73,76,77,99,100,130^ | **4**^69,71,98,130^ |  |
|  | | *≥65* | **6**^37,54,66,74,77,78^ | **6**^37,54,66,74,77,78^ | **3**^54,74,77^ | **1**^66^ |
|  | | *≥60* | **1**^41^ | **1**^41^ | **1**^41^ |  |
|  | | *≥55* | **2**^73,101^ | 1^101^ | 1^73^ | **1**^101^ |
|  | | *≥50* | **1**^76^ | **1**^76^ |  |  |
| *2. Gender* | | | **10**^6,37,54,55,69,74,76,77,98,100^ | **9**^6,37,54,55,74,76,77,98,100^ | **2**^54,69^ |  |
| *3. Initial GCS* | *<15* | | **7**^37,41,66,73,74,77,101^ | **6**^37,41,66,73,74,101^ | **4**^37,73,74,77^ | **2**^66,101^ |
|  | *GCS* | | **7**^6,55,69,73,77,98,100^ | **4**^6,55,73,77,100^ | **2**^69,98^ |  |
|  | *GCS = 14* | | **1**^6^ |  | **1**^6^ |  |
|  | *GCS = 13* | | **1**^6^ |  | **1**^6^ |  |
| *4. CT findings* | *Midline shift CT/Mass effect* | | **5**^6,55,66,76,100^ | **4**^6,66,76,100^ | **4**^6,55,76,100^ | **1**^66^ |
|  | *Marshall classification* | | **2**^41,73^ | **2**^41,73^ |  |  |
|  | *SDH >10 mm* | | **1**^54^ | **1**^54^ | **1**^54^ |  |
|  | *EDH >10 mm* | | **1**^54^ | **1**^54^ | **1**^54^ |  |
|  | *ICH volume >10 mL* | | **1**^78^ | **1**^78^ | **1**^78^ |  |
|  | *Mean volume* | | **1**^55^ | **1**^55^ | **1**^55^ |  |
|  | *Maximal thickness* | | **1**^55^ |  | **1**^55^ |  |
|  | *Volume ED* | | **1**^100^ | **1**^100^ | **1**^100^ |  |
|  | *Volume SDH* | | **1**^100^ | **1**^100^ |  |  |
|  | *Volume ICB* | | **1**^100^ | **1**^100^ |  |  |
| *5. Type of isolated injury* | *Contusion* | | **1**^37,78^ | **1**^37,78^ |  |  |
|  | *SDH* | | **3**^37,73,98^ | **2^37,73^** | **1**^98^ |  |
|  | *EDH* | | **3**^37,73,98^ | **2**^37,73^ | **1**^98^ |  |
|  | *SAH* | | **3**^37,73,98^ | **2**^37,73^ | **2**^73,98^ |  |
|  | *Mixed* | | **1**^73,98^ | **1**^73^ | **1**^98^ |  |
|  | *ICB* | | **1**^73^ | **1**^73^ |  |  |
| *6. Presence of (includes mixed injuries)* | *Contusion* | | **3**^37,76^ | **3**^37,76^ |  |  |
|  | *SDH* | | **5**^6,37,76,100,101^ | **5**^6,37,76,100,101^ | **1**^37^ |  |
|  | *EDH* | | **5**^6,37,76,100,101^ | **5**^6,37,76,100,101^ |  |  |
|  | *SAH* | | **4**^6,37,76,100,101^ | **4**^6,37,76,100,101^ |  |  |
|  | *Fracture* | | **4**^6,74,76,100^ | **4**^6,74,76,100^ | **1**^100^ |  |
|  | *Displaced/depressed fracture* | | **2**^54,66^ | **2**^54,66^ | **1**^54^ |  |
|  | *Base of skull fracture* | | **2^74,76^** | **2**^74,76^ |  |  |
|  | *pneumocranium* | | **1**^100^ | **1**^100^ |  |  |
|  | *ICB* | | **3**^6,100,101^ | **3**^6,100,101^ |  |  |
|  | *IVH* | | **3**^6,76,100^ | **3**^6,76,100^ |  |  |
|  | *Diffuse axonal injury* | | **1**^76^ | **1**^76^ |  |  |
|  | *2+ lesions* | | **4**^6,74,77,100^ | **4**^6,74,77,100^ | **2**^74,77^ |  |
|  | *3+ lesions* | | **1**^6^ | **1**^6^ |  |  |
| *7. Subdural with* | *contusion* | | **1**^55^ | **1**^55^ | **1**^55^ |  |
|  | *SAH* | | **1**^55^ | **1**^55^ | **1**^55^ |  |
| *8. Non-isolated head Injury* | | | **1^66^** | **1^66^** |  | **1**^66^ |
| *9. BP* | | | **7**^54,73,76,77,98-100^ | **6**^54,73,76,77,99,100^ | **2**^73,98^ |  |
| *10. Pre-admission hypotension* | | | **1^66^** | **1^66^** |  |  |
| *11. HR* | | | **4**^54,73,98,99^ | **3**^54,73,99^ | **1**^98^ |  |
| *12. RR* | | | **1**^98^ | **1**^98^ |  |  |
| *13. Pre-injury hypoxia* | | | **1^66^** | **1^66^** |  |  |
| *14. Intoxication* | | | **2**^54,55^ | **2**^54,55^ |  |  |
| *15. Coagulopathy : including any anti-coagulant use* | | | **6**^6,41,55,77,98,100^ | **5**^6,41,55,77,100^ | **1**^98^ |  |
| *16. Warfarin use* | | | **3**^37,76,101^ | **3**^37,76,101^ |  |  |
| *20. Warfarin or anti-platelet* | | | **2**^78,100^ | **2**^78,100^ |  |  |
| *17. PT/INR* | | | **3**^6,74,101^ | **3**^6,74,101^ |  |  |
| *18. aPPT* | | | **1**^6,101^ | **2**^6,101^ |  |  |
| *19 Platelet count <100,000* | | | **1**^54^ | **1**^54^ | **1**^54^ |  |
| *20. Platelet count <50,000* | | | **1**^55^ | **1**^55^ |  |  |
| *21. Hb <10* | | | **1**^54^ | **1**^54^ |  |  |
| *22. Hb* | | | **2**^74,100^ | **2**^74,100^ |  |  |
| *23. WCC* | | | **1**^100^ | **1**^100^ | **1**^100^ |  |
| *24. Aspirin* | | | **3**^37,76,101^ | **3**^37,76,101^ |  |  |
| *25. Clopidogrel* | | | **3**^37,76,101^ | **3**^37,76,101^ |  |  |
| *25. Any anti-platelet* | | | **2**^55,66,87^ | **1**^55,66^ | **1**^87^ |  |
| *26. ISS* | | | **11**^6,69,71,73,76,77,98-101,130^ | **9**^6,41,73,76,77,99-101,130^ | **7**^6,69,71,73,98,100,130^ |  |
| *27. (H)AIS* | | | **5**^41,73,76,99,101^ | **5**^41,73,76,99,101^ | **1**^73^ |  |
| *28. LOC* | | | **1**^54^ | **1**^54^ | **1**^54^ |  |
| *29. Mechanism of injury*  *(unqualified)* | | | **2**^54,55^ | **2**^54,55^ |  |  |
| *30. Non-fall from standing* | | | **1**^66^ | **1**^66^ |  |  |
| *31. Fall* | | | **2**^37,77^ | **2**^37,77^ |  |  |
| *32. Assault* | | | **1**^37^ | **1**^37^ |  |  |
| *33. RTC* | | | **4**^37,74,76,77^ | **4**^37,74,76,77^ |  |  |
| *34. Pedestrian struck* | | | **1**^37^ | **1**^37^ |  |  |
| *35. Bicycle struck* | | | **1**^37^ | **1**^37^ |  |  |
| *36. Lactate* | | | **1**^54^ | **1**^54^ | **1**^54^ |  |
| *37. Base deficit* | | | **1**^54^ | **1**^54^ | **1**^54^ |  |
| *38. Comorbidities* | | *HTN* | **3**^37,76,100^ | **3**^37,76,100^ |  |  |
|  | | *Diabetes* | **2**^76,100^ | **2**^76,100^ |  |  |
|  | | *Old CVA* | **2**^76,100^ | **2**^76,100^ |  |  |
|  | | *IHD* | **2**^76,100^ | **2**^76,100^ |  |  |
|  | | *Arrhythmia* | **1**^100^ | **1**^100^ |  |  |
|  | | *Liver disease* | **1**^100^ | **1**^100^ |  |  |
|  | | *CKD* | **1**^100^ | **1**^100^ |  |  |
|  | | *AKI* | **1**^100^ | **1**^100^ |  |  |
|  | | *Any high risk* | **1^66^** | **1^66^** |  |  |
| *39. Smoking* | | | **1**^55^ | **1**^55^ |  |  |
| *40. Time to first CT* | | | **2**^73,76^ | **2**^73,76^ |  |  |
| *41. Statin therapy* | | | **1**^100^ | **1**^100^ |  |  |

AIS, ________; AKI, ______; aPPT, activated partial thromboplastin time; BP, blood pressure; CKD, _________; CT, computed tomography; CVA, cerebrovascular accident; ED, emergency department; EDH, extra-dural hemorrhage; GCS, Glasgow Comas Scale; Hb, ______; HR, heart rate; HTN, hypertension; ICB, intra-cranial bleed; ICH, intra-cerebral hemorrhage ; IHD, ______; INR, international normalized ratio ; ISS, Injury Severity Score; LOC, loss of consciousness; PT, ______;RR, relative risk; RTC, random controlled trial; SAH, subarachnoid hemorrhage; SDH, subdural hematoma, WCC, white cell count.

Supplementary Table 6. Forest Plots of Within Study Risk Factors’ Effect on the Risk of Neurosurgery or Clinical Deterioration

**Meta-analysis of effect of initial GCS = 15 on risk of clinical deterioration/neurosurgery**

**
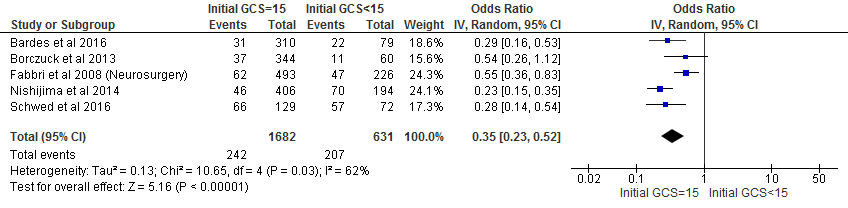
**

**Meta-analysis effect of isolated subarachnoid hemorrhage vs. any other injury on clinical deterioration/neurosurgery**


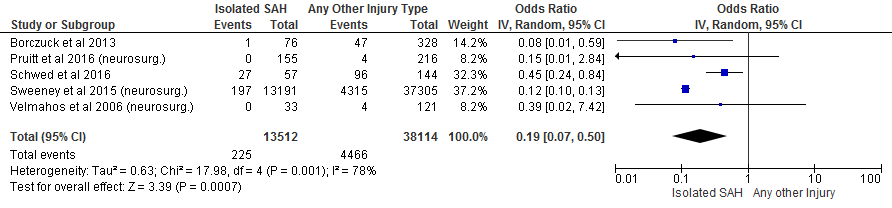


**Meta-analysis effect of isolated extra-dural vs. any other injury on clinical deterioration/neurosurgery**


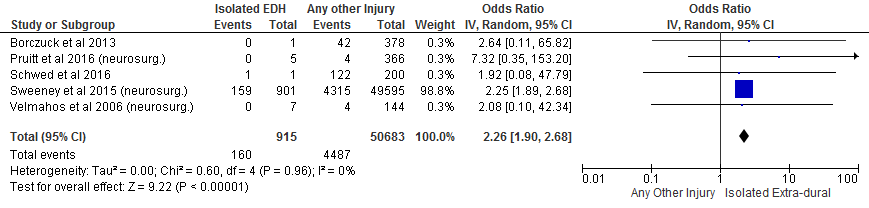


**Meta-analysis effect of isolated subdural vs. any other injury on clinical deterioration/neurosurgery
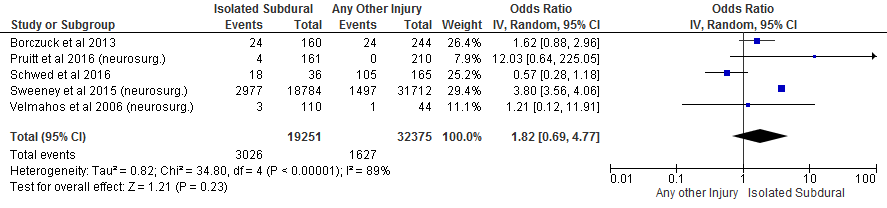
**

**Meta-analysis effect of isolated contusion vs. any other injury on clinical deterioration/neurosurgery**

**
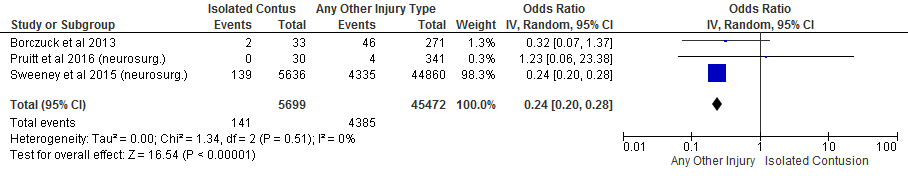
**

**Meta-analysis of effect of coagulopathy use on clinical deterioration/neurosurgery**


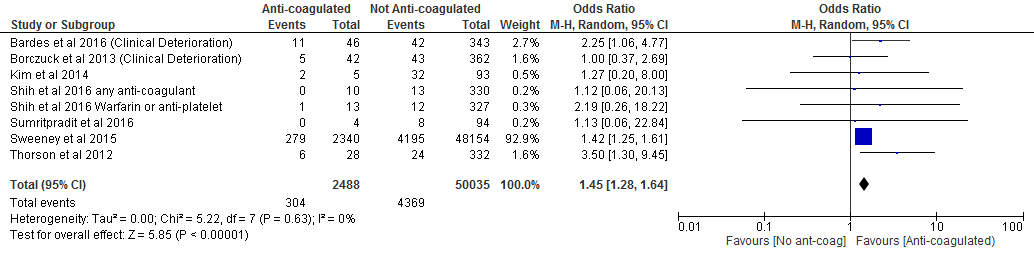


**Meta-analysis of effect of aspirin/anti-platelet use on clinical deterioration/neurosurgery**

**
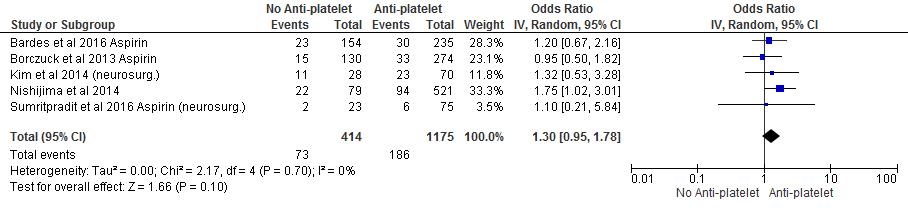
**

**Meta-analysis effect of clopidogrel/anti-platelet use on clinical deterioration/ neurosurgery**


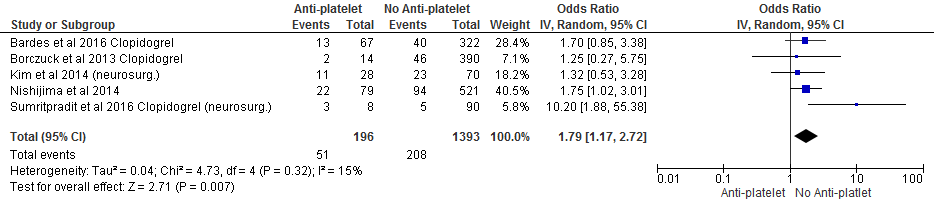


**SUPPLEMENTARY FIG. 1**. Pooled risk of clinical deterioration stratified by the injury type identified by initial CT imaging.


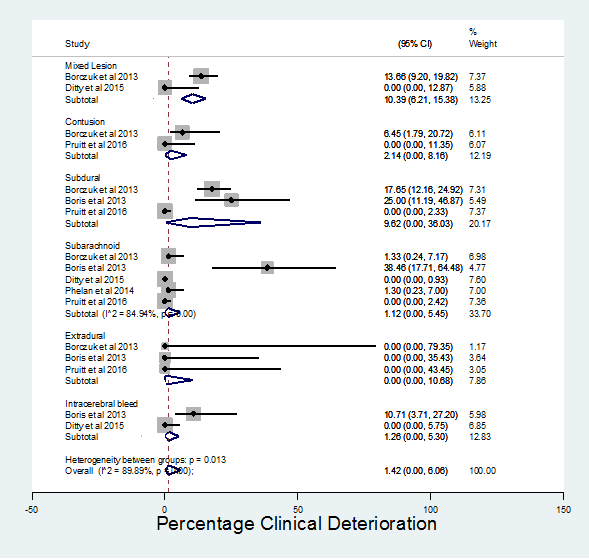

Supplement: Supplemental data [file Supp_Data.docx]
